# Supplementary material for: Communication from Learned to Innate Olfactory Processing Centers Is Required for Memory Retrieval in Drosophila
Source: Neuron. 2018 Nov 7;100(3):651–668.e8. doi: 10.1016/j.neuron.2018.08.037 (PMC6226615; doi:10.1016/j.neuron.2018.08.037)
Supplement: Document S1. Figures S1–S8 [file mmc1.pdf]

Neuron, Volume *100*

Supplemental Information

Communication from Learned to Innate Olfactory Processing Centers Is Required for Memory Retrieval in *Drosophila*

Michael-John Dolan, Ghislain Belliart-Guérin, Alexander Shakeel Bates, Shahar Frechter, Aurélie Lampin-Saint-Amaux, Yoshinori Aso, Ruairí J.V. Roberts, Philipp Schlegel, Allan Wong, Adnan Hammad, Davi Bock, Gerald M. Rubin, Thomas Preat, Pierre-Yves Plaçais, and Gregory S.X.E. Jefferis

Figure S1: Further anatomical analysis of 71D08-LexA and 37G11-GAL4. Related to Figure 1.

(A) Partial Z-projection of R71D08-LexA driving GFP. Expression of MBON-ɑ2sc is observed in the dorsal LH and along the vertical lobe of the MB. Representative image of n=3 stacks. (B-C) Immunohistochemistry of PD2a1/b1 to determine major neurotransmitter. (B) Colocalization of GABA (magenta) and R37G11-GAL driving GFP (green) expression indicates that PD2a1/b1 neurons are not GABAergic. (C) Colocalization of VGlut (magenta) and split-GAL4 line LH989 driving mVenus (green) expression indicates that PD2a1/b1 neurons are not glutamatergic (yellow arrows label sample bouton). For B-C the left panel is a composite while the middle and right panels represent single channels. (D) Immunohistochemistry of LH989 driving RFP in PD2a1/b1 neurons (green) and GFP genetically driven in cholinergic neurons by $Cha^{MI04508}$-LexA::QFAD. The cluster of PD2a1/b1 cell bodies is labelled with an orange circle. The scale bar for A and D is 30µm and 5µm for B-C. For B-D, each image is a representative slice from n=4 brains.

Figure S2: Anatomical and behavioural analysis of two other cell-types identified in the overlap screen. Related to Figure 1.

(A) A segmented image of AV6a1. (B) A segmented image of the identified SIP cell-type. (C) Z-projection of double labelling between AV6a1 dendrites and MBON-ɑ2sc. MBON-ɑ2sc presynapses are labelled with Brp(d3)::mCherry (magenta) while AV6a1 is labelled with membrane-bound GFP (in green). (D) Z-projection of double labelling between AV6a1 dendrites and MBON-ɑ2sc. The MBON-ɑ2sc presynapses are labelled with Brp(d3)::mCherry (magenta) while the SIP-1 neuron is labelled with membrane-bound GFP (in green). Note that this LexA line contains both MBON-ɑ2sc (dorsal) and MBON-α′3ap (ventral) axons (E) Unsegmented Z-projection of identified AV6a1 GAL4 line, Insite0089-GAL4, driving CD8::GFP (Green) with an nc82 counterstain (magenta). Representative stack from n=3 brains. This brain is registered to the JFRC2 template brain. (F) Unsegmented Z-projection of identified SIP-1 GAL4 line, R58G03-GAL4, driving CD8::GFP (Green) with an nc82 counterstain (magenta). This brain is registered to the JFRC2 template brain. Data from Virtual Fly Brain (http://www.virtualflybrain.org/site/vfb_site/home.htm) (G) Flies expressing Shi^{ts} driven by Insite0089-GAL4, and their genotypic controls, were trained with a single-cycle protocol at permissive temperature and tested 3h later at restrictive temperature. There was no significant difference in memory performance between the three groups ($n = 17$, $F_{(2,50)} = 2.29$, $p = 0.11$). (H)

Flies expressing Shi^{ts} driven by R58G03-GAL4, and their genotypic controls, were trained with a single-cycle protocol at permissive temperature and tested 3h later at restrictive temperature. There was no significant difference in memory performance between the three groups (n = 14, $F_{(2,41)} = 1.61$, p = 0.21). Data are presented as mean±SEM. Scale bar is 30µm for A-B and E-F, 5µm for C-D.

Figure S3: Permissive temperature controls for 37G11-GAL4 memory retrieval experiments. Related to Figure 1.
When assayed at the permissive temperature, flies expressing Shi^{ts} through R37G11-GAL4 driver performed similarly to their controls for immediate memory (A: n = 8, $F_{(2,23)} = 3.52$, p = 0.048, no difference between groups in pairwise comparisons), 3h-memory (B: n = 16, $F_{(2,47)} =4.23$, p = 0.02, no difference between +/UAS-Shi^{ts} and 37G11>UAS-Shi^{ts} groups in pairwise comparisons), and long-term memory (C: n = 9–10, $F_{(2,28)} = 0.53$, p = 0.59). Data are presented as mean±SEM.

Figure S4: Permissive temperature controls for split-GAL4 memory retrieval experiments. Related to Figure 2.
When assayed at the permissive temperature, flies expressing Shi^{ts} through LH989 and LH991 performed similarly to their controls for immediate memory after 1x training (A: LH991, n = 12, $F_{(2,35)} = 0.14$, p = 0.87; B: LH989, n = 12–13, $F_{(2,36)} = 3.66$, p = 0.037, no difference between 37G11/+ and 37G11>UAS-Shi^{ts} groups in pairwise comparisons), for 3h-memory after 1x training (C: LH991, n = 15, $F_{(2,44)} = 0.28$, p = 0.76; D: LH989, n = 10, $F_{(2,29)} = 1.79$, p = 0.19), and for long-term memory after spaced training (E: LH991, n = 17, $F_{(2,47)} = 0.27$, p = 0.76; F: LH989, n = 7–9, $F_{(2,24)} = 2.10$, p = 0.15). Data are presented as mean±SEM.

Figure S5: Single-cell analysis of PD2a1/b1 GAL4 and split-GAL4 lines. Effectors in landing site VK5 and Electron microscopy reconstruction strategy. Related to Figure 2-4.
(A) An example a MultiColor FlipOut (MCFO) experiment. A single neuron is labelled in green while the counterstain is nc82 (magenta). The counterstain is registered to the JFRC2013 template brain (B) Skeletonized representations of all 22 single-neurons derived from MCFO registered to JFRC2013 (grey). (C-D) More detailed view of all 22 skeletons from an anterior (C) and dorsal (D) view. For C-D, only the LH (red) and calyx (blue) neuropil regions are labelled. Scale bar is 30µm. (E) Confocal z-projection of R71D08-LexA driving expression of

TdTomato (green) in landing site VK5. Expression recapitulates that of the R71D08 enhancer including MBON-ɑ2sc and MBON-α′3ap. Counterstain is nc82 (magenta). Representative stack of n=3 brains. (F) Cross-section of the PD2a1/b1 tract in the electron microscopy dataset. Neurons confirmed to be PD2a1/b1 are labelled in light (PD2a1) and dark green (PD2b1). Possible primary neurites in the PD2 tract are labelled with a purple dot (178). The x,y,z position of the tract in the ssTEM whole brain volume is 482684, 206960, 151200 nm, section 4320. (G) Dorsal view of PD2a1 (top) and PD2b1 (bottom) skeletons from MCFO data (blue) and EM reconstructions (green), shown with the LH (green), CA (purple), SIP (yellow) and SMP (orange). Black line bisecting PD2 primary neurite tract indicate position of EM section in (F). Slight offset between light and EM is thought to be a product of registration error (H'-H'') NBLAST clustering PD2a1 and PD2b1 arbors from EM reconstructed, FlyCircuit and MCFO data using only their axonal compartments (H') or dendritic compartments (H'') Skeletons are colored by the group to which they belong in the dendrogram. In (H) a1 and b1 neurons cluster apart. EM neurons are more similar to each other likely because they are from the same brain whereas the MCFO and FlyCircuit data are from different brains, and have been co-registered. In (H) the main two subdivisions denote the two routes PD2a1/b1 axons take around the MB (purple) vertical lobe. Labels name include the split-Gal4 line that generated the MCFO, or the Gal4 line driven by a neurotransmitter synthesis/processing protein used to generate the FlyCircuit flip-out data, or indicate an EM neuron in Figure 4B''.

Figure S6: Summary Electron Microscopy data for MBON-ɑ2sc and PD2a1/b1. Related to Figure 4.

(A) Bar chart showing the neuropils targeted by the ipsilateral 'fly's right' MBON-ɑ2sc axon, showing the postsynapses, presynapses and cable that the MBON has in each neuropil. Inset, presynapses across the MBON axon shown coloured by neuropil location. (B) The neuropils targeted by whole 'fly's right' PD2a1/b1 neurons and the PD2a1/b1 postsynapses, presynapses and cable length in each. Inset, postsynapses and presynapses across the PD2a1/b1 neurons shown coloured by neuropil location. (C) Stripchart shows the cable length in µms for PD2a1/b1 dendrites, PD2b1 dendrites and PDa1/b1 axons. Stacked bar chart shows the proportion of cable and postsynapses that were found on microtubule-containing backbone and microtubule free 'twigs'. Inset, visualisation of backbone (red) and twigs (green) in PD2a1#1. (D) Number of presynapses and postsynapses plotted for the presumptive axon and dendrite of individual PD2a1 and PD2b1 neurons. The different categories and region of the cell are represented by distinct

colors (PD2a1 or PD2b1) and shapes (Calyx, LH or whole compartments). Grey crosses indicate the mean. LH=lateral horn, MB_CA_R = mushroom body calyx, SIP=superior intermediate protocerebrum, SLP=superior lateral protocerebrum, SMP=superior medial protocerebrum, CRE = crepine, SCL = superior clamp, _R = fly's right (ipsilateral), _L = fly's left (contralateral).

Figure S7: Schematics for live calcium imaging training paradigms. Related to Figure 5. Schematics of the 3 hour conditioning protocols used for *in vivo* imaging of odor responses after training with Oct (red) as CS+ (A) or Mch (blue) as CS+ (B). Left represents the actual training paradigm where electric shock (orange spikes) is presented alongside the CS+. Right panel represents the control unpaired protocol where electric shock is presented two minutes before olfactory stimulation. (C) Each of the training paradigm in A-B is considered a single unit which is used to space train flies to generate LTM for imaging 24 hours after training (left panel). The training paradigm described in A or B (depending on CS+) is repeated 5 times with an intertrial interval (ITI) of 15 minutes. For the unpaired control at 24 hours (right panel), the unpaired protocols from A or B (depending on which odor is CS+) is repeated 5 times with an intertrial interval (ITI) of 15 minutes.

Figure S8: Permissive experiments for innate attraction experiments and genotype controls for MBON-ɑ2sc silencing during calcium imaging. Related to Figure 6 and 7. (A-D) Control experiments where animals did not have the LexAop-Shibire^{ts1} transgene to control for effects of the temperature shifts on olfactory coding. (A) Response of PD2a1/b1 axons to Oct, recorded by calcium imaging without MBON-ɑ2sc silencing. On left, time traces of normalized GCaMP3 fluorescence (see methods) are shown at permissive (blue) and restrictive (red) temperature in response to Oct stimulation (light blue bar). On right, the integral of the absolute odor responses for each individual fly at the permissive (blue) and restrictive (red) temperatures are plotted, which no change in response to Oct after MBON-ɑ2sc silencing (n=6, paired t-test=0.1). (B) Response of PD2a1/b1 axons to Mch, recorded by calcium imaging without MBON-ɑ2sc silencing. On left, time traces of normalized GCaMP3 fluorescence (see methods) are shown at permissive (blue) and restrictive (red) temperature in response to Mch stimulation (light blue bar). On right, the integral of the absolute odor responses for each individual fly at the permissive (blue) and restrictive (red) temperatures are plotted, which no change in response to Mch after MBON-ɑ2sc silencing (n=6, paired t-test=0.25). (C) Response of PD2a1/b1 axons to ethyl acetate, recorded by calcium imaging without MBON-ɑ2sc silencing.

On left, time traces of normalized GCaMP3 fluorescence (see methods) are shown at permissive (blue) and restrictive (red) temperature in response to ethyl acetate stimulation (light blue bar). On right, the integral of the absolute odor responses for each individual fly at the permissive (blue) and restrictive (red) temperatures are plotted, which no change in response to ethyl acetate after MBON-ɑ2sc silencing (n=6, paired t-test=0.98). (D) Response of PD2a1/b1 axons to vinegar, recorded by calcium imaging without MBON-ɑ2sc silencing. On left, time traces of normalized GCaMP3 fluorescence (see methods) are shown at permissive (blue) and restrictive (red) temperature in response to vinegar stimulation (light blue bar). On right, the integral of the absolute odor responses for each individual fly at the permissive (blue) and restrictive (red) temperatures are plotted, which no change in response to vinegar after MBON-ɑ2sc silencing (n=6, paired t-test=0.53). (E) Flies expressing Shi^{ts} driven by either LH989 or LH991 showed no impairment to apple cider vinegar relative to their genotype controls at the permissive temperature (n=9-10, $F_{(4,47)}$=0.42, p=0.79). (F) Flies expressing Shi^{ts} driven by either LH989 or LH991 showed no impairment to ethyl acetate relative to their genotype controls at the permissive temperature (n=8-11, $F_{(4,45)}$=0.41, p=0.80). Legend shows the shading representing each genotype.

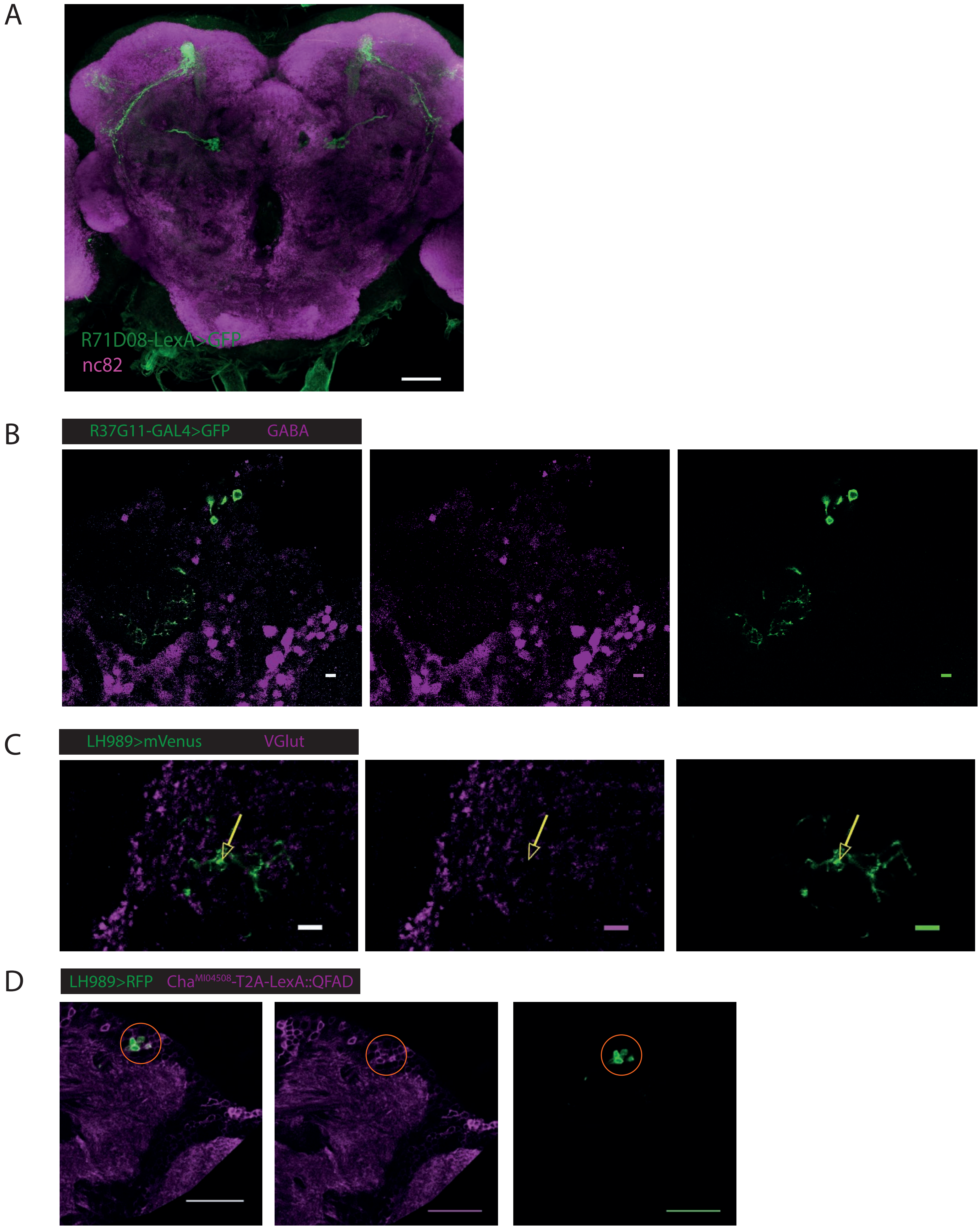
A
R71D08-LexA>GFP
nc82
B
R37G11-GAL4>GFP
GABA
C
LH989>mVenus
VGlut
D
LH989>RFP
Cha^MI04508-T2A-LexA::QFAD

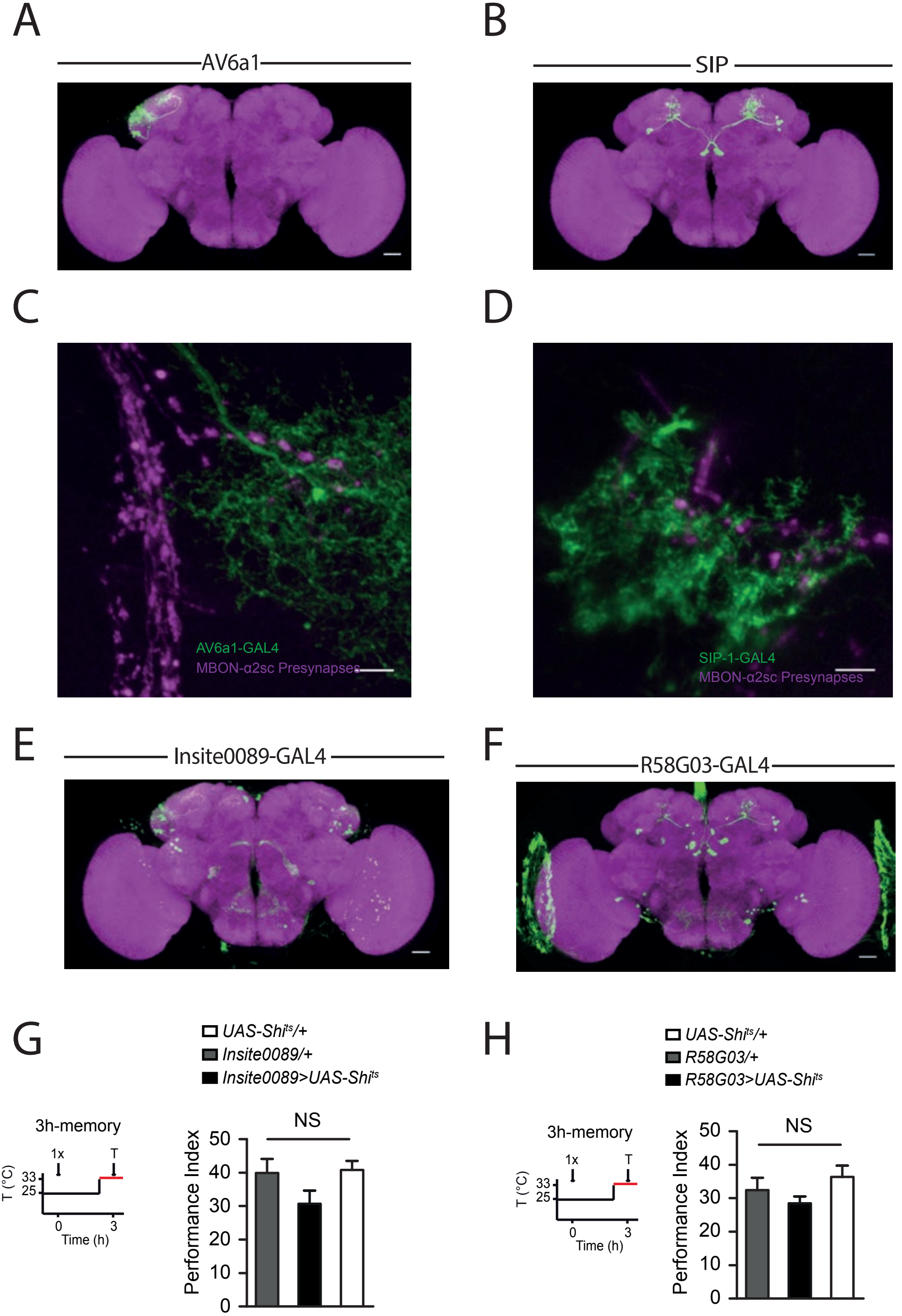

A
AV6a1
B
SIP
C
AV6a1-GAL4
MBON-α2sc Presynapses
D
SIP-1-GAL4
MBON-α2sc Presynapses
E
Insite0089-GAL4
F
R58G03-GAL4
G
UAS-Shi^ts/+
Insite0089/+
Insite0089>UAS-Shi^ts
3h-memory
1x
T
T (°C)
33
25
0
3
Time (h)
NS
Performance Index
50
40
30
20
10
0
H
UAS-Shi^ts/+
R58G03/+
R58G03>UAS-Shi^ts
3h-memory
1x
T
T (°C)
33
25
0
3
Time (h)
NS
Performance Index
50
40
30
20
10
0

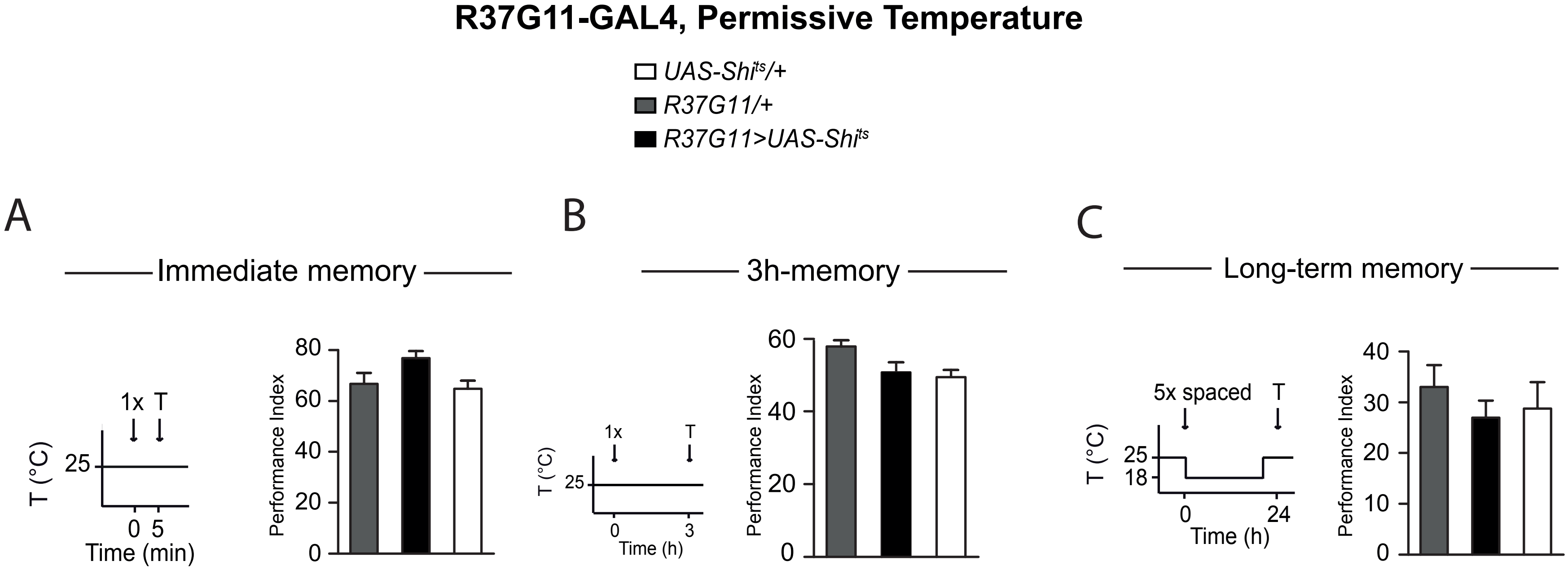
R37G11-GAL4, Permissive Temperature
UAS-Shi^ts/+
R37G11/+
R37G11>UAS-Shi^ts
A
Immediate memory
1x T
T (°C)
25
0 5
Time (min)
Performance Index
0
20
40
60
80
B
3h-memory
1x T
T (°C)
25
0 3
Time (h)
Performance Index
0
20
40
60
C
Long-term memory
5x spaced T
T (°C)
25
18
0 24
Time (h)
Performance Index
0
10
20
30
40

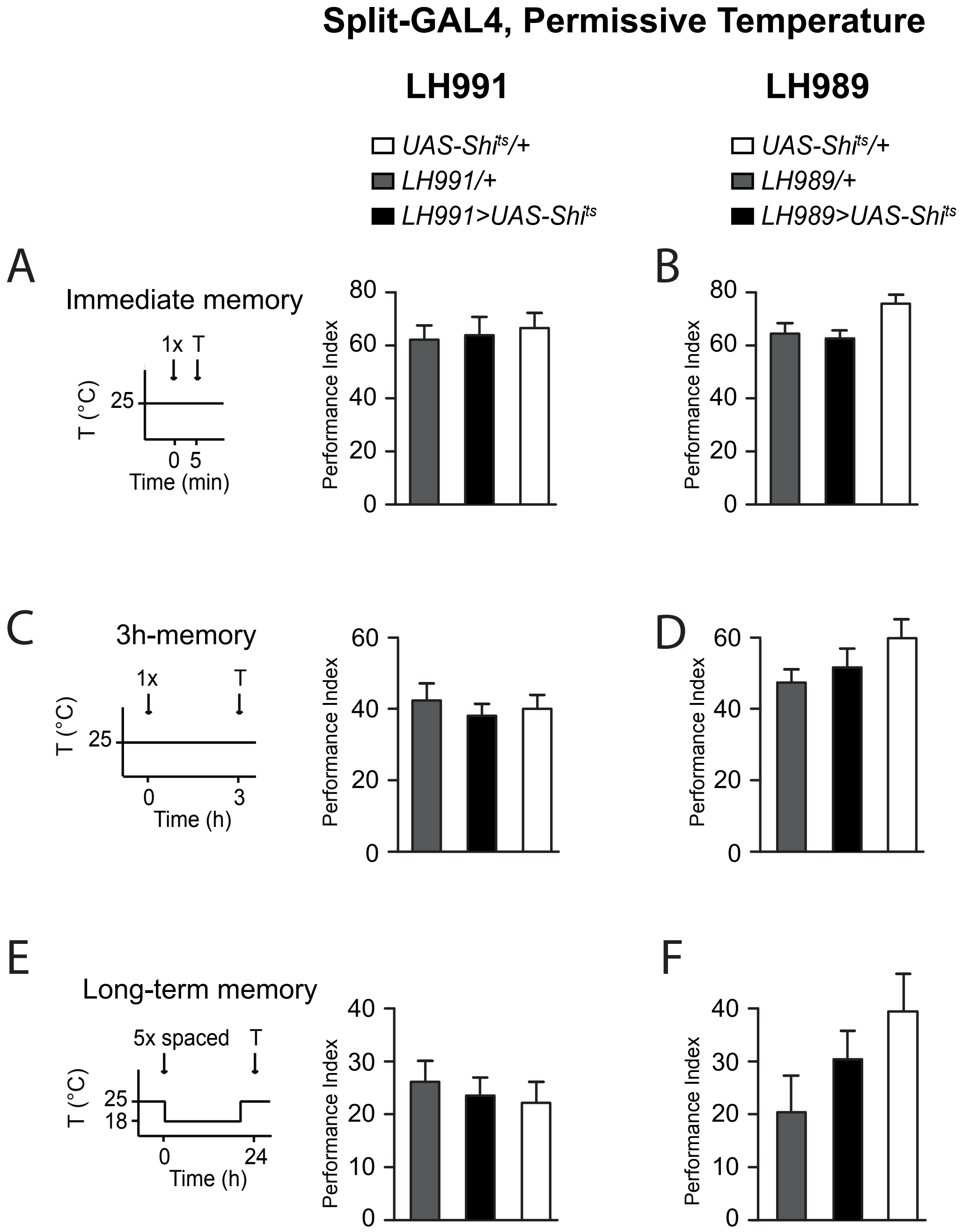
Split-GAL4, Permissive Temperature
LH991
LH989
UAS-Shi^ts/+
LH991/+
LH991>UAS-Shi^ts
UAS-Shi^ts/+
LH989/+
LH989>UAS-Shi^ts
A
Immediate memory
1x T
T (°C)
25
0 5
Time (min)
Performance Index
0 20 40 60 80
B
C
3h-memory
1x T
T (°C)
25
0 3
Time (h)
0 20 40 60
D
E
Long-term memory
5x spaced T
T (°C)
25
18
0 24
Time (h)
0 10 20 30 40
F

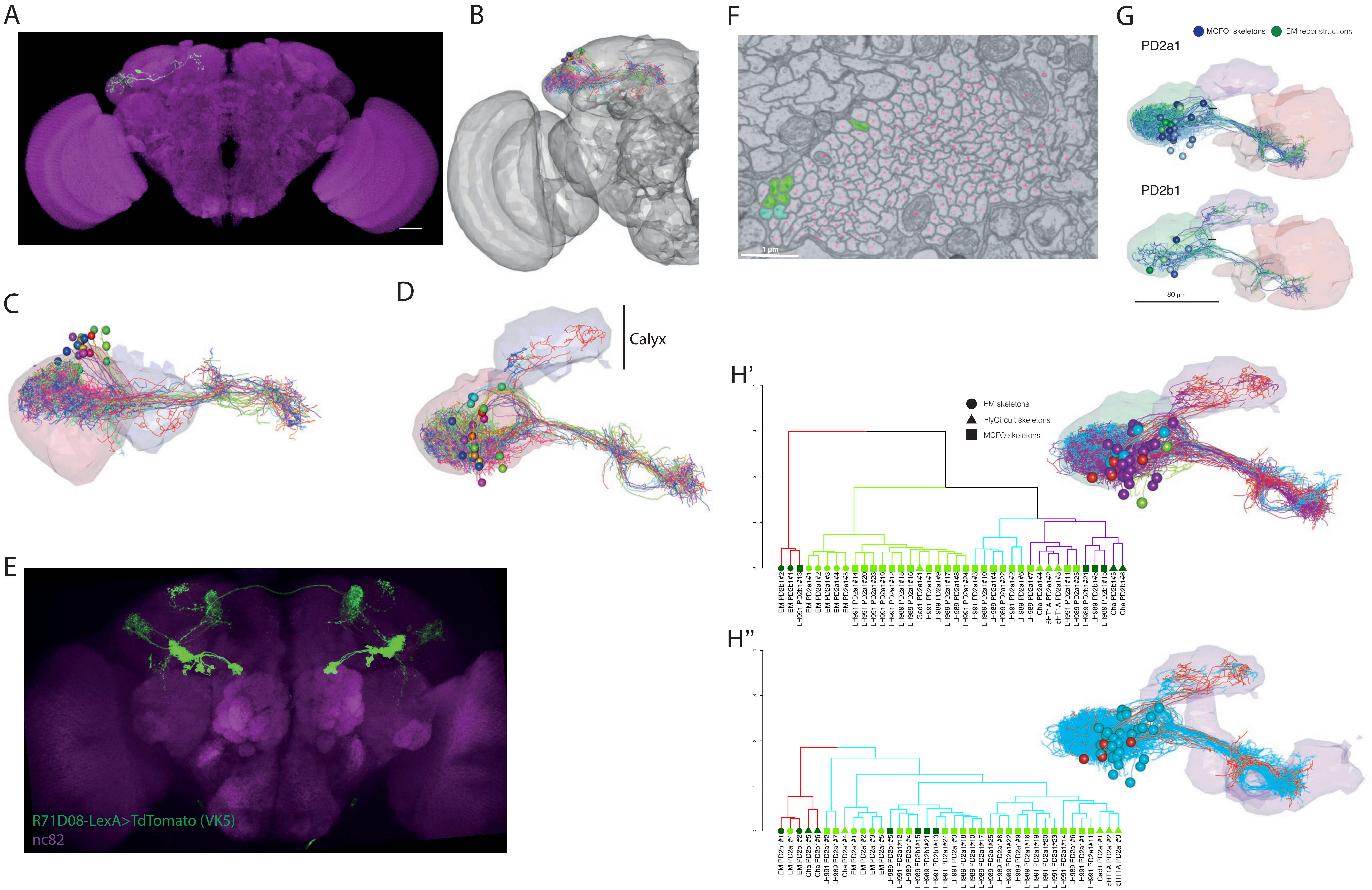

A
B
C
D
Calyx
E
R71D08-LexA>TdTomato (VK5)
nc82
F
1 µm
G
MCFO skeletons
EM reconstructions
PD2a1
PD2b1
80 µm
H'
EM skeletons
FlyCircuit skeletons
MCFO skeletons
0
1
2
3
4
EM PD2b1#2
EM PD2b1#1
LH991 PD2b1#13
EM PD2a1#1
EM PD2a1#2
EM PD2a1#3
EM PD2a1#4
EM PD2a1#5
LH991 PD2a1#14
LH991 PD2a1#20
LH991 PD2a1#23
LH991 PD2a1#19
LH991 PD2a1#12
LH989 PD2a1#18
LH989 PD2a1#16
Gad1 PD2a1#1
LH991 PD2a1#1
LH989 PD2a1#9
LH989 PD2a1#17
LH989 PD2a1#8
LH991 PD2a1#24
LH991 PD2a1#3
LH989 PD2a1#10
LH989 PD2a1#4
LH989 PD2a1#22
LH991 PD2a1#2
LH989 PD2a1#6
LH989 PD2a1#7
Cha PD2a1#4
5HT1A PD2a1#2
5HT1A PD2a1#3
LH991 PD2a1#11
LH989 PD2a1#25
LH989 PD2b1#21
LH989 PD2b1#5
LH989 PD2b1#15
Cha PD2b1#5
Cha PD2b1#6
H''
0
1
2
3
4
EM PD2b1#1
EM PD2a1#4
EM PD2b1#2
Cha PD2b1#5
Cha PD2b1#6
LH991 PD2a1#2
LH989 PD2a1#7
Cha PD2a1#4
EM PD2a1#1
EM PD2a1#2
EM PD2a1#3
EM PD2a1#5
LH989 PD2b1#5
LH991 PD2a1#12
LH989 PD2a1#4
LH989 PD2b1#15
LH989 PD2b1#21
LH991 PD2b1#13
LH991 PD2a1#24
LH991 PD2a1#3
LH989 PD2a1#18
LH989 PD2a1#10
LH989 PD2a1#17
LH989 PD2a1#25
LH989 PD2a1#8
LH989 PD2a1#22
LH989 PD2a1#9
LH989 PD2a1#16
LH991 PD2a1#19
LH991 PD2a1#20
LH991 PD2a1#23
LH991 PD2a1#14
LH989 PD2a1#6
LH991 PD2a1#1
LH991 PD2a1#11
Gad1 PD2a1#1
5HT1A PD2a1#2
5HT1A PD2a1#3

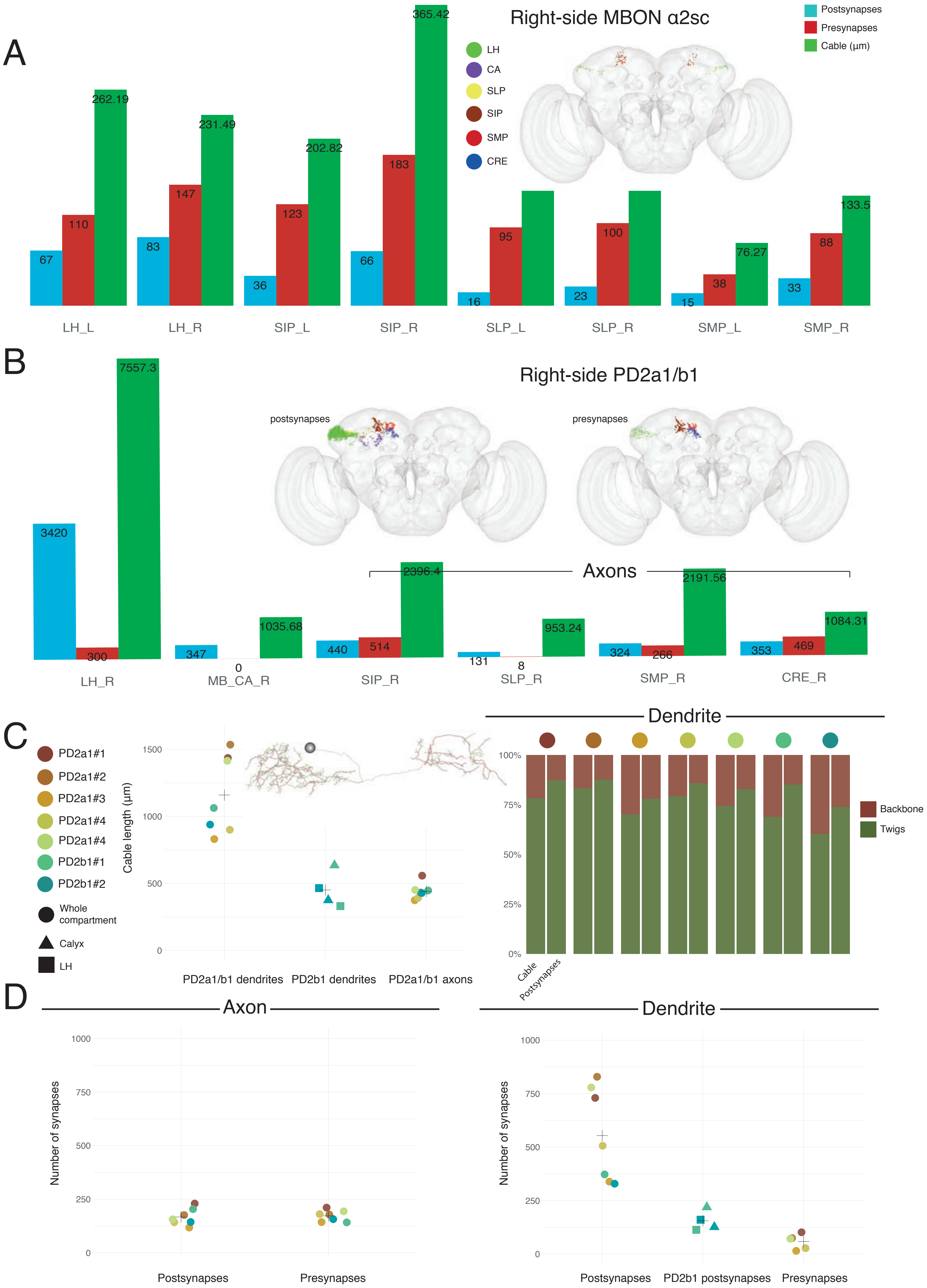

A
Right-side MBON α2sc
Postsynapses
Presynapses
Cable (μm)
LH
CA
SLP
SIP
SMP
CRE
67
110
262.19
83
147
231.49
36
123
202.82
66
183
365.42
16
95
23
100
15
38
76.27
33
88
133.5
LH_L
LH_R
SIP_L
SIP_R
SLP_L
SLP_R
SMP_L
SMP_R
B
Right-side PD2a1/b1
postsynapses
presynapses
Axons
3420
300
7557.3
347
0
1035.68
440
514
2396.4
131
8
953.24
324
266
2191.56
353
469
1084.31
LH_R
MB_CA_R
SIP_R
SLP_R
SMP_R
CRE_R
C
PD2a1#1
PD2a1#2
PD2a1#3
PD2a1#4
PD2a1#4
PD2b1#1
PD2b1#2
Whole compartment
Calyx
LH
Cable length (μm)
1500
1000
500
0
PD2a1/b1 dendrites
PD2b1 dendrites
PD2a1/b1 axons
Dendrite
100%
75%
50%
25%
0%
Backbone
Twigs
Cable
Postsynapses
D
Axon
Number of synapses
1000
750
500
250
0
Postsynapses
Presynapses
Dendrite
Number of synapses
1000
750
500
250
0
Postsynapses
PD2b1 postsynapses
Presynapses

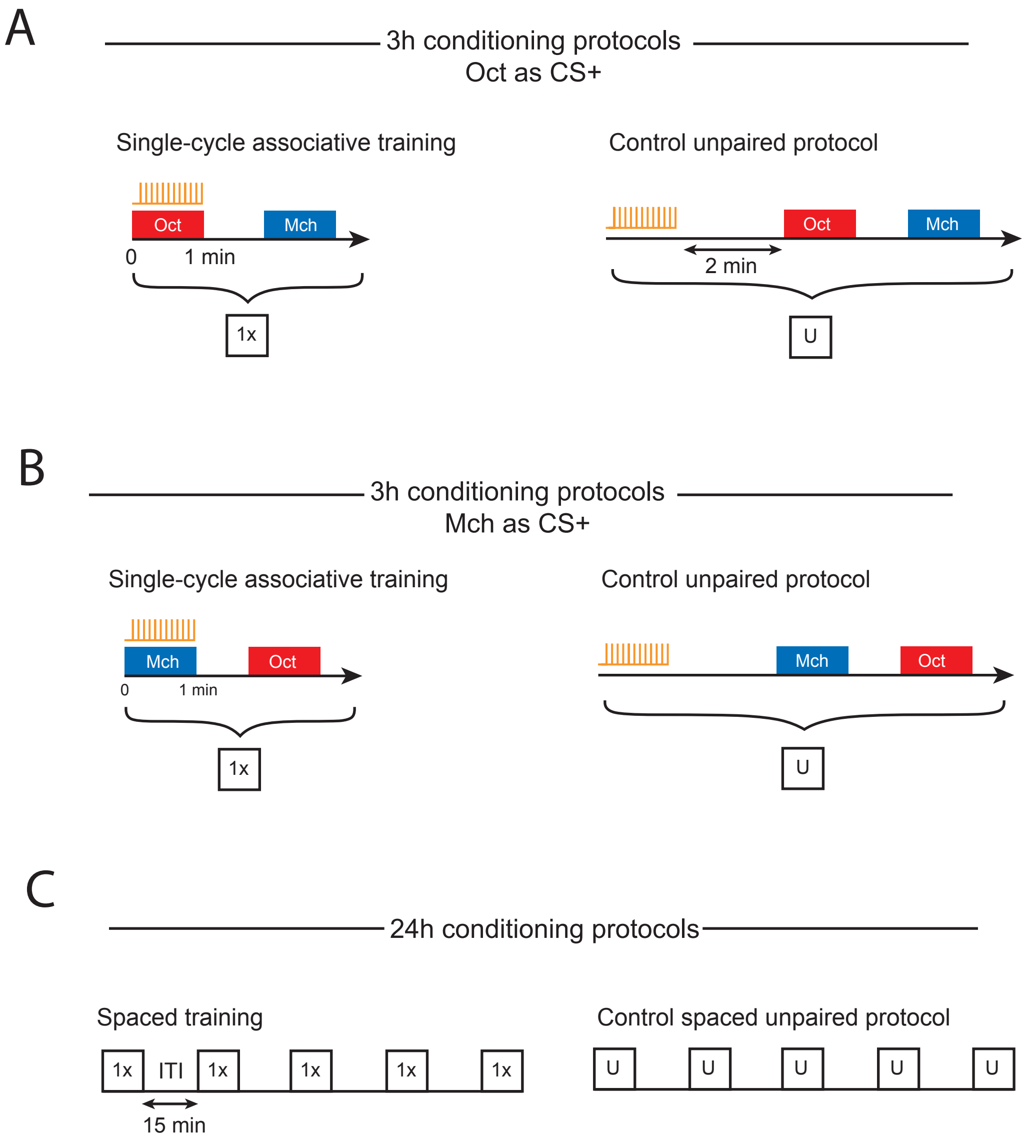
A
3h conditioning protocols
Oct as CS+
Single-cycle associative training
Oct
Mch
0
1 min
1x
Control unpaired protocol
Oct
Mch
2 min
U
B
3h conditioning protocols
Mch as CS+
Single-cycle associative training
Mch
Oct
0
1 min
1x
Control unpaired protocol
Mch
Oct
U
C
24h conditioning protocols
Spaced training
1x
ITI
1x
1x
1x
1x
15 min
Control spaced unpaired protocol
U
U
U
U
U

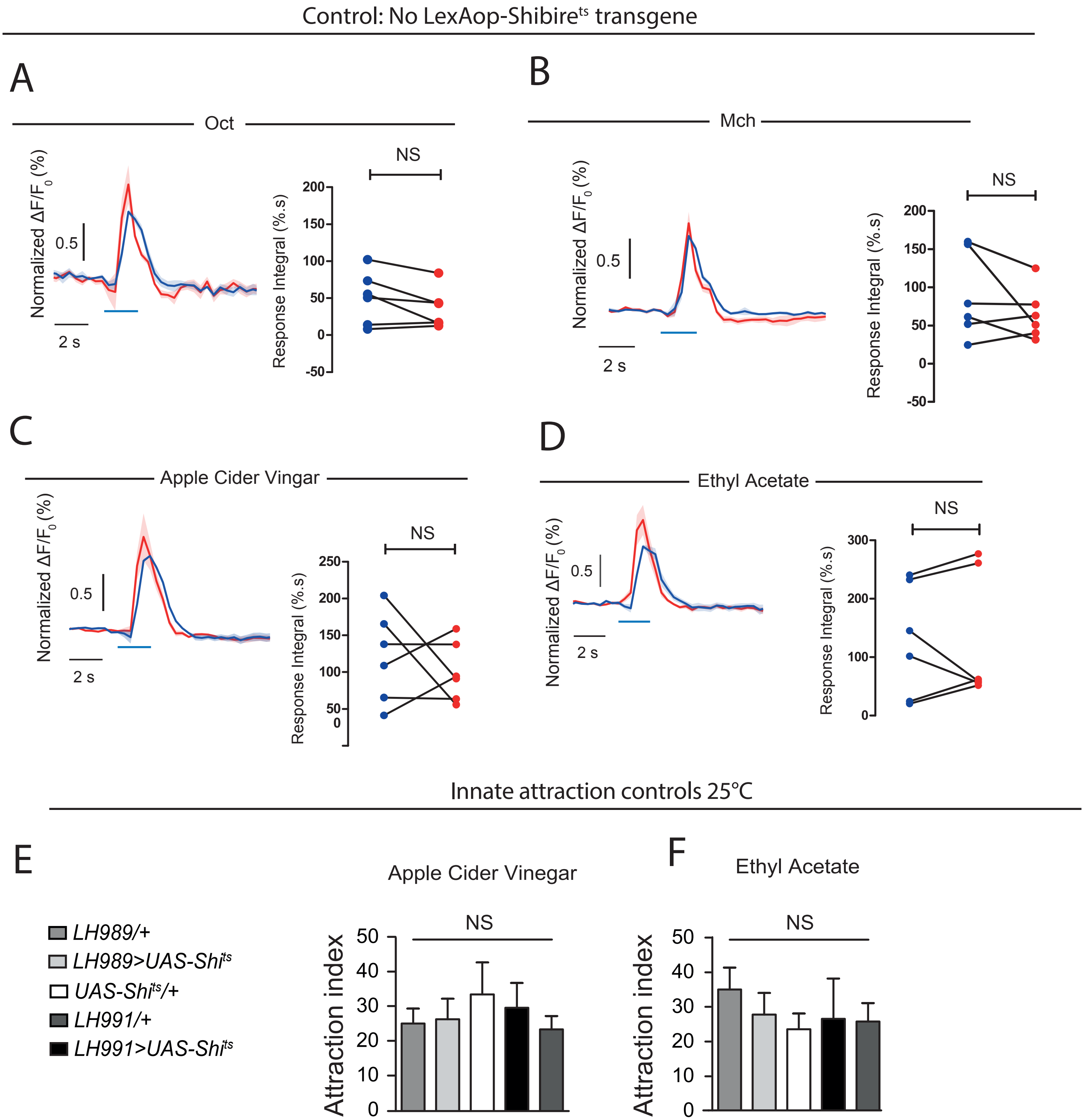

Control: No LexAop-Shibire^ts transgene
A
Oct
Normalized ΔF/F0 (%)
0.5
2 s
Response Integral (%.s)
200
150
100
50
0
-50
NS
B
Mch
Normalized ΔF/F0 (%)
0.5
2 s
Response Integral (%.s)
200
150
100
50
0
-50
NS
C
Apple Cider Vingar
Normalized ΔF/F0 (%)
0.5
2 s
Response Integral (%.s)
250
200
150
100
50
0
NS
D
Ethyl Acetate
Normalized ΔF/F0 (%)
0.5
2 s
Response Integral (%.s)
300
200
100
0
NS
Innate attraction controls 25°C
E
Apple Cider Vinegar
LH989/+
LH989>UAS-Shi^ts
UAS-Shi^ts/+
LH991/+
LH991>UAS-Shi^ts
Attraction index
50
40
30
20
10
0
NS
F
Ethyl Acetate
Attraction index
50
40
30
20
10
0
NS
