# Supplementary material for: Communication from Learned to Innate Olfactory Processing Centers Is Required for Memory Retrieval in Drosophila
Source: Neuron. 2018 Nov 7;100(3):651–668.e8. doi: 10.1016/j.neuron.2018.08.037 (PMC6226615; doi:10.1016/j.neuron.2018.08.037)
Supplement: Document S2. Article plus Supplemental Information [file mmc6.pdf]

Article

Neuron

Communication from Learned to Innate Olfactory Processing Centers Is Required for Memory Retrieval in *Drosophila*

Highlights

- Specific *Drosophila* lateral horn neurons mediate innate attraction to food odors
- The same neurons receive plastic odor information from the mushroom body
- Recall after associative learning depends on reduced drive to lateral horn neurons
- Connectomics circuit for integration of learned and innate odor representations

Authors

Michael-John Dolan, Ghislain Belliart-Guérin, Alexander Shakeel Bates, ..., Thomas Preat, Pierre-Yves Plaçais, Gregory S.X.E. Jefferis

Correspondence

pierre-yves.placais@espci.fr (P.-Y.P.), jefferis@mrc-lmb.cam.ac.uk (G.S.X.E.J.)

In Brief

Sensory stimuli can engage both learned and innate behaviors. Dolan et al. identify neurons in *Drosophila* that directly integrate unlearned and plastic odor representations; they are required for innate approach to food odors but also learned aversive recall.

Dolan et al., 2018, Neuron *100*, 651–668
November 7, 2018 © 2018 MRC Laboratory of Molecular Biology.
Published by Elsevier Inc.
https://doi.org/10.1016/j.neuron.2018.08.037

Check for updates

CellPress

CellPress

Communication from Learned to Innate Olfactory Processing Centers Is Required for Memory Retrieval in *Drosophila*

Michael-John Dolan,[1,2,5] Ghislain Belliart-Guérin,[3,5] Alexander Shakeel Bates,[1] Shahar Frechter,[1] Aurélie Lampin-Saint-Amaux,[3] Yoshinori Aso,[2] Ruairí J.V. Roberts,[4] Philipp Schlegel,[1,4] Allan Wong,[2] Adnan Hammad,[1] Davi Bock,[2] Gerald M. Rubin,[2] Thomas Preat,[3] Pierre-Yves Plaçais,[3,6,*] and Gregory S.X.E. Jefferis[1,4,6,7,*]
[1]Division of Neurobiology, MRC Laboratory of Molecular Biology, Cambridge CB2 0QH, UK
[2]Janelia Research Campus, Howard Hughes Medical Institute, Ashburn, VA, USA
[3]Genes and Dynamics of Memory Systems, Brain Plasticity Unit, CNRS, ESPCI Paris, PSL Research University, 75005 Paris, France
[4]Department of Zoology, University of Cambridge, Cambridge CB2 3EJ, UK
[5]These authors contributed equally
[6]Senior author
[7]Lead Contact
*Correspondence: pierre-yves.placais@espci.fr (P.-Y.P.), jefferis@mrc-lmb.cam.ac.uk (G.S.X.E.J.)
https://doi.org/10.1016/j.neuron.2018.08.037

SUMMARY

The behavioral response to a sensory stimulus may depend on both learned and innate neuronal representations. How these circuits interact to produce appropriate behavior is unknown. In *Drosophila*, the lateral horn (LH) and mushroom body (MB) are thought to mediate innate and learned olfactory behavior, respectively, although LH function has not been tested directly. Here we identify two LH cell types (PD2a1 and PD2b1) that receive input from an MB output neuron required for recall of aversive olfactory memories. These neurons are required for aversive memory retrieval and modulated by training. Connectomics data demonstrate that PD2a1 and PD2b1 neurons also receive direct input from food odor-encoding neurons. Consistent with this, PD2a1 and PD2b1 are also necessary for unlearned attraction to some odors, indicating that these neurons have a dual behavioral role. This provides a circuit mechanism by which learned and innate olfactory information can interact in identified neurons to produce appropriate behavior.

INTRODUCTION

The action of natural selection on evolutionary timescales endows animal species with behavioral responses to stimuli of particular ethological relevance. In addition, most animals show adaptive responses based on learning during their lifetime. Learning may modify an unlearned response. However, it remains unknown how memory recall interacts with innate sensory representations to produce the most appropriate behavior. This study explores this general issue using the *Drosophila* olfactory system. Olfaction is a shallow sense (in terms of neural processing) with a privileged connection to memory systems in many species (Su et al., 2009). Genetic tractability and numeric simplicity make the *Drosophila* brain an ideal model to study this interaction at a neural circuit level, whereas the similarity in organization of peripheral olfactory circuits makes it possible that neurobiological principles may also be shared deeper in the brain between insects and mammals (Su et al., 2009).

In *Drosophila*, olfactory sensory neurons project to specific glomeruli in the antennal lobe (Masse et al., 2009). Following local computations, excitatory uniglomerular projection neurons (PNs) make divergent connections to two higher processing regions, the lateral horn (LH) and the mushroom body (MB) (Masse et al., 2009), in addition to other antennal lobe (AL) outputs (Strutz et al., 2014; Tanaka et al., 2012). The prevailing model of olfactory processing proposes a clear functional division between these regions: the MB is required for learning, consolidation, and retrieval of olfactory memories, whereas the LH is thought to mediate innate behavior (Keene and Waddell, 2007; Masse et al., 2009). Many studies have confirmed the necessity of the MB for associative memory, where a reward or punishment (the unconditioned stimulus [US]) is associated with one odor (the conditioned stimulus [CS+]), but not with a second odor (CS−) (Keene and Waddell, 2007). The role of the LH in innate behavior has been inferred from experiments that silenced the MB and observed innate olfactory responses (Heimbeck et al., 2001; Parnas et al., 2013). However, no studies to date have directly examined the behavioral functions of LH neurons in olfaction.

Mapping studies show that PNs from different glomeruli have stereotyped axonal projections in the LH (Jefferis et al., 2007; Marin et al., 2002; Wong et al., 2002), consistent with a role in innate olfactory behaviors. Anatomical and physiological analyses have shown a role for specific *Drosophila* LH neurons in processing pheromone cues relevant to sex-specific behaviors such as courtship and aggression (Jefferis et al., 2007; Kohl et al., 2013; Liang et al., 2013; Ruta et al., 2010). Recent results

This is an open access article under the CC BY license (http://creativecommons.org/licenses/by/4.0/).

have shown that some LH neurons can also show stereotyped responses to general olfactory stimuli (Fişek and Wilson, 2014; Strutz et al., 2014) and are stereotypically connected to input PNs (Fişek and Wilson, 2014). In addition, new large-scale data have confirmed response stereotypy and showed that different LH neurons have wide variations in odor tuning and may encode odor categories (Fişek and Wilson, 2014; Frechter et al., 2018).

In contrast to the LH, MB neurons are extremely well characterized (Aso et al., 2014a). The dendrites of intrinsic MB neurons (Kenyon cells) are localized to a region called the calyx, where they sample incoming PN axons in an apparently random manner (Caron et al., 2013). Kenyon cells have parallel, axonal fibers that form five different lobes, with three distinct branching patterns that define as many Kenyon cell types (Aso et al., 2014a). Anatomical analysis has subdivided the lobes into 15 compartments, each innervated by specific dopaminergic input neurons (DANs) and MB output neurons (MBONs) (Aso et al., 2014a). These compartments are anatomically and physiologically distinct (Cohn et al., 2015; Hige et al., 2015a), although each Kenyon cell axon synapses in all compartments of each lobe (Cohn et al., 2015).

Odors are sparsely represented in the Kenyon cell assembly, so only a subset of axon terminals will release neurotransmitters upon olfactory stimulation (Honegger et al., 2011). Electric shock, the US during aversive learning, activates a subset of DANs so that, when US and CS+ are coincident, the subset of olfaction-driven Kenyon cells also receives dopaminergic input within specific compartments. This coincident input produces compartment-specific synaptic plasticity (Bouzaiane et al., 2015; Cohn et al., 2015; Hige et al., 2015a; Liu et al., 2012; Owald et al., 2015), changing the response of that compartment's MBON to the CS+. MBONs function in valence behaviors, and a modified response to the trained odor may bias the fly's behavior toward avoidance or attraction depending on the compartment (Aso et al., 2014b; Owald et al., 2015). One of these output neurons, MBON-α2sc (also known as MB-V2α), projects from the MB to several brain regions, including the LH (Hige et al., 2015a; Séjourné et al., 2011). Optogenetic stimulation of the entire V2 cluster (MBON-α2sc, MBON-α′3m, and MBON-α′3ap) drives approach behavior, but activation of MBON-α2sc alone does not lead to any change in valence behavior (Aso et al., 2014b). Previous work has demonstrated that MBON-α2sc is required for the retrieval of aversive olfactory memories across short, medium, and long timescales (Hige et al., 2015a; Séjourné et al., 2011) although not necessary for the recall of appetitive memories (Séjourné et al., 2011). Recordings from MBON-α2sc demonstrated that it is broadly odor-responsive (Hige et al., 2015b) but depresses its response to CS+ after training (Hige et al., 2015a; Séjourné et al., 2011). This depression to the trained odor response is thought to spread to unknown downstream neural circuits mediating aversive olfactory memory retrieval (Aso et al., 2014b; Hige et al., 2015a; Séjourné et al., 2011), in addition to an increased drive of negative valence MBONs (Aso et al., 2014b; Bouzaiane et al., 2015; Owald et al., 2015). Given the presumed role of the LH in innate olfaction, the function of the MB to LH projection of MBON-α2sc is unclear. Is memory information transmitted to the LH, and if so, is this communication required for retrieval of the aversive memory?

In this study, we examine the behavioral function of this connection between the presumed innate and learned olfactory processing centers. We use computational anatomy and microscopy to identify two LH output neuron cell types (PD2a1 and PD2b1) postsynaptic to MBON-α2sc. We use whole-brain electron microscopy connectomics (Zheng et al., 2018) to verify this synaptic connectivity and then test the function of these cell types in behavior. Contrary to the model described above, where the LH mediates only innate olfactory behavior, PD2a1 and PD2b1 are necessary for memory retrieval. We generate new split-GAL4 lines (Luan et al., 2006; Pfeiffer et al., 2010) specifically targeting these neurons to confirm their necessity for memory recall. Calcium imaging shows that PD2a1 and PD2b1 olfactory responses are depressed after training, similar to the MBON. Additional connectomics work finds direct olfactory PN input onto PD2a1 and PD2b1 dendrites, identifying these cells as responsive to food or appetitive odors. We then demonstrate that PD2a1 and PD2b1 neurons are necessary for innate olfactory attraction for several odors. This work provides a model for the interaction of innate and learned sensory information.

RESULTS

Identifying LH Neurons Postsynaptic to MBON-α2sc

To understand the role of information flow from the MB and LH, we first sought to identify postsynaptic neurons in the LH that receive input from MBON-α2sc. We developed a computational pipeline to find MBON-α2sc postsynaptic candidates. We used *in silico* overlap of GAL4 expression patterns to identify candidate postsynaptic partners of MBON-α2sc. Using image registration (Jefferis et al., 2007), we created a mask of the MBON-α2sc axonal terminals expressing a presynaptically localized marker (Christiansen et al., 2011). We then calculated pixel overlap of the mask with registered images of published GAL4 lines (Gohl et al., 2011; Jenett et al., 2012). We ranked lines by a relative "overlap score" for each brain that compared the GFP signal within the MB peduncle to exclude lines with MB Kenyon cell expression, which could complicate behavioral analysis. Scores for approximately 3,500 GAL4 lines (Figure 1A) were mostly close to zero or negative (having little or no LH overlap but strong peduncle expression). We focused on the top ~100 lines (97th percentile). After excluding lines labeling MBON-α2sc, the top hits identified 5 cell types putatively postsynaptic to MBON-α2sc in the dorsal LH. Many lines were excluded because of broad expression, so there are likely other LH neurons that we could not analyze.

We next generated a LexA line to orthogonally control MBON-α2sc (Figure S1A). Double-labeling of MBON presynapses and various LH cell types furthered the number of candidates. Two cell types had potential synaptic sites identified by double labeling and high-resolution confocal microscopy: LH output neuron cell types posterior dorsal 2a1 and b1 (PD2a1 and PD2b1) (Figures 1B and 1C; see below for single-neuron data) and anterior ventral 6a1 (AV6a1) (Figures S2A and S2C). These names are based on a hierarchical nomenclature for over 150 LH cell types (Frechter et al., 2018). We also repeated this analysis for MBON axonal processes in the superior intermediate protocerebrum

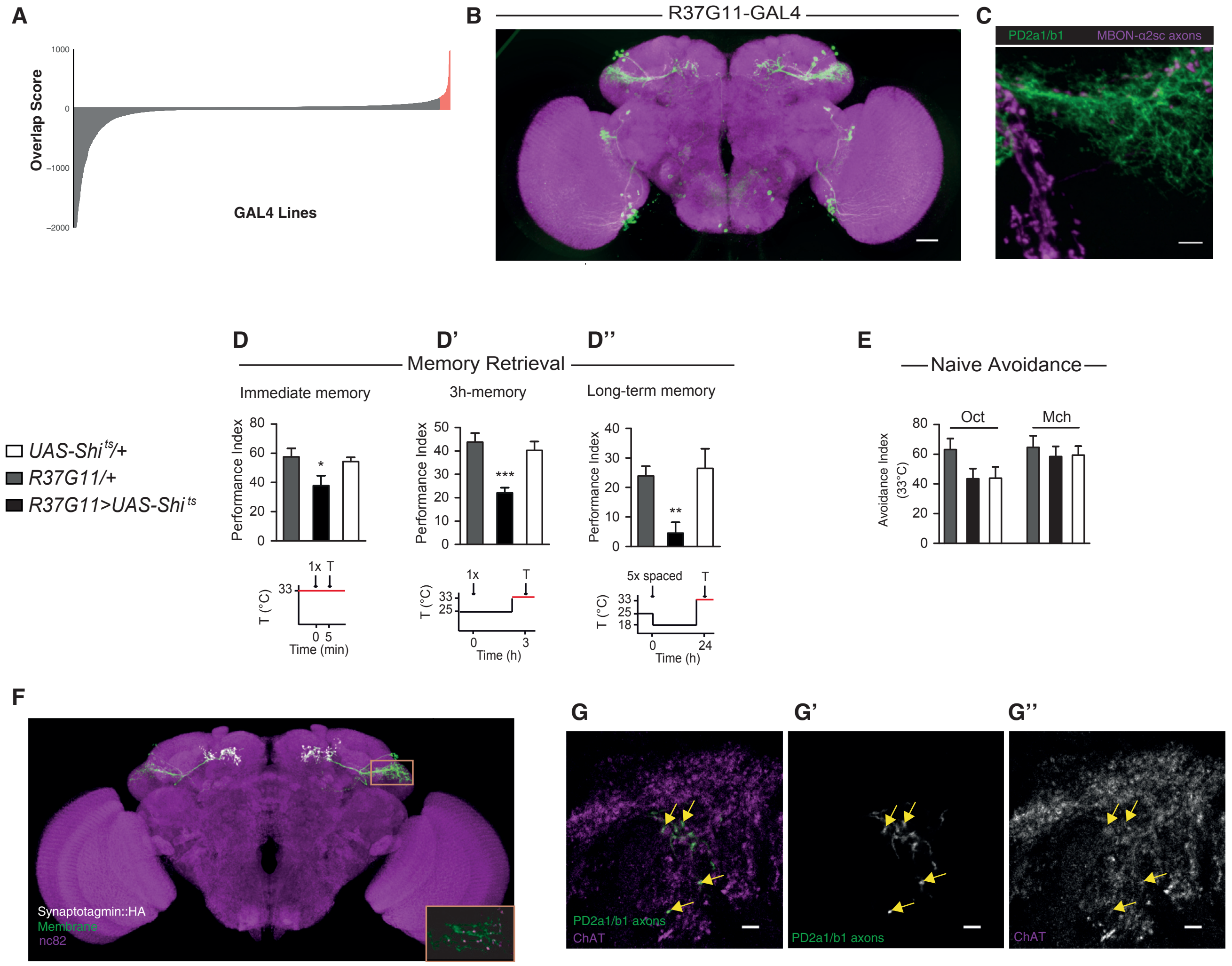

Figure 1. PD2a1 and PD2b1 Are Postsynaptic to MBON-α2sc and Necessary for Memory Retrieval

(A) Distribution of LH overlap scores for MBON-α2sc axon mask versus 3,500 GAL4 lines. Scores > 97 percentile are labeled in red, y axis clipped <−2,000.

(B) Sparsest GAL4 line labeling cell type PD2a1 and PD2b1, R37G11-GAL4 (image from https://www.janelia.org/gal4-gen1). Scale bar, 30 μm.

(C) z-projection of double labeling. MBON axons are labeled in magenta, and PD2a1 and PD2b1 are labeled with membrane-bound GFP (in green). This LexA line contains both MBON-α2sc (dorsal) and MBON-α′3ap (ventral). Scale bar, 5 μm. The image is representative of n = 4.

(D–D") Flies with R37G11-GAL4 driving Shi^{ts} and genotypic controls were trained and tested with the illustrated protocols (restrictive temperature indicated in red). Silencing PD2a1 and PD2b1 neurons impaired immediate memory after single-cycle training (D; n = 12–13, $F_{(2,36)} = 3.79$, p = 0.033), 3-hr memory after single-cycle training (D'; n = 9, $F_{(2,26)} = 12.07$, p = 0.0002), and long-term memory after spaced training (D"; n = 9, $F_{(2,26)} = 6.28$, p = 0.0064).

(E) Flies expressing Shi^{ts} driven by the 37G11-GAL4 driver showed normal olfactory avoidance to octanol (Oct) and methylcyclohexanol (Mch) compared with their controls at the restrictive temperature (Oct, n = 14, $F_{(2,41)} = 2.41$, p = 0.10; Mch, n = 14, $F_{(2,41)} = 0.23$, p = 0.79). Data are presented as mean ± SEM.

(F) Confocal z-projection of PD2a1 and PD2b1 driving both membrane-bound GFP (green) and Synaptotagmin-HA (gray). PD2a1 and PD2b1 has been manually segmented. The orange rectangle represents the inset. Inset: a single slice of PD2a1 and PD2b1 dendrites showing punctate Synaptotagmin-HA, indicating dendritic presynapses. The image is representative of n = 5.

(G–G"). ChAT immunohistochemistry demonstrating that PD2a1 and PD2b1 neurons are cholinergic. The images show a representative slice (n = 4 stacks). Scale bars, 5 μm.

See also Figures S1–S3.

(SIP), identifying only one candidate postsynaptic cell type, SIP-1 (Figures S2B and S2D).

PD2a1 and PD2b1 Are Necessary for Memory Retrieval

We identified the sparsest GAL4 lines for the three selected cell types identified and screened for memory retrieval defects when the neurons were silenced in an aversive olfaction-associative conditioning paradigm. LH cell types expressed the temperature-sensitive silencer $shibire^{ts1}$ (Kitamoto, 2001), which inhibits neuronal signaling at high temperatures (33°C, the restrictive temperature). By raising the temperature during a memory test 3 hr after aversive olfactory conditioning, we could silence these

neurons to probe their role in memory recall (Séjourné et al., 2011).

Silencing the AV6a1 and SIP cell type GAL4 lines had no detectable effect on memory (Figures S2G and S2H). However, silencing PD2a1 and PD2b1 neurons with R37G11-GAL4 impaired 3-hr memory retrieval relative to genotype (Figure 1D') and temperature (Figure S3B) controls. We extended these analyses of PD2a1 and PD2b1 to include immediate and long-term memory, which also require MBON-α2sc (Bouzaiane et al., 2015; Séjourné et al., 2011). Silencing PD2a1 and PD2b1 neurons attenuated memory retrieval for both memory phases (Figures 1D and 1D'') versus controls (Figures S3A and S3C). Surprisingly, PD2a1 and PD2b1 inhibition had no effect on naive olfactory avoidance to the two training odors at the concentrations used in our memory assay (Figure 1E), so the observed phenotype was not due to defective innate olfactory processing, the proposed function of LH neurons. These results indicate that PD2a1 and PD2b1 activity is necessary during memory recall.

We confirmed that PD2a1 and PD2b1 are primarily an LH output cell type by expressing hemagglutinin (HA)-fused synaptotagmin (Syt::HA) to label presynapses (Robinson et al., 2002; Figure 1F). We also observed some presynapses in the presumptive LH dendrites (Figure 1F). We next determined their neurotransmitter profiles. PD2a1 and PD2b1 was ChAT-immunoreactive (Figures 1G–G'') but gamma-aminobutyric acid (GABA)- and *Drosophila* vesicular glutamate transporter (dVGlut)-negative (Figures S1B and S1C; Chen et al., 2017). These neurons, therefore, appear to be excitatory cholinergic LH outputs, a conclusion we confirmed using a genetic approach to label cholinergic neurons (Diao et al., 2015; Figure S1D).

Generation and Characterization of Cell-Type-Specific Split-GAL4 Lines

Although R37G11-GAL4 is relatively specific, it contained some other cell types that could confound our behavioral results. To confirm that PD2a1 and PD2b1 neurons are responsible for the memory retrieval deficit, we generated split-GAL4 lines (Luan et al., 2006; Pfeiffer et al., 2010) specific to PD2a1 and PD2b1 in the central brain (Figures 2A and 2B). We focused on two split-GAL4 lines, LH989 and LH991, that used the same R37G11 enhancer as the original GAL4 line, reasoning that they were most likely the same neurons. Both of these split-GAL4 lines also labeled neurons in the ventral nerve cord (VNC); however, these VNC cell types were different between lines (Figures 2A and 2B). We compared the number of PD2a1 and PD2b1 neurons labeled by each line; R37G11-GAL4 labeled 6.9 ± 0.6 cells, whereas LH989 and LH991 contained 5.25 ± 0.5 and 5.67 ± 0.8 neurons, respectively.

To confirm that PD2a1 and PD2b1 are involved in the retrieval of several memory phases, immediately after single-cycle training, on the middle-term timescale ($\sim$3 hr), and 24 hr after spaced training, we repeated our behavioral experiments with these sparse split-GAL4 lines. When flies were tested at the restrictive temperature to silence PD2a1 and PD2b1, memory performance was impaired under all three conditions compared with genotype controls (Figures 2C–2H). This ranged from mild attenuation immediately after training (Figures 2C and 2D) to full impairment for long term memory (LTM) retrieval (Figures 2G and 2H), similar to phenotypes silencing MBON-α2sc (Bouzaiane et al., 2015; Séjourné et al., 2011). This defect was due to neuronal silencing because identical flies at the permissive temperature had no memory recall deficits (Figure S4). Finally, we verified that silencing PD2a1 and PD2b1 neurons with split-GAL4 lines had no effect on innate olfactory avoidance for the two training odors (Figures 2I and 2J), confirming that this is a specific defect in memory recall. Output from cell type PD2a1 and PD2b1 are therefore necessary for retrieval of aversive olfactory memory, with the same characteristics as MBON-α2sc.

To understand the anatomy of PD2a1 and PD2b1 cells, we labeled single neurons in R37G11-GAL4 and the two split-GAL4 lines with MultiColor FlpOut (MCFO) (Nern et al., 2015; Figures S5A–S5C), isolating 22 single neurons from the PD2a1 and PD2b1 cell type. 3 of 22 labeled neurons also projected to the MB calyx (this projection is also visible in R37G11-GAL4, LH989, and LH991), whereas all other neurons appeared indistinguishable (Figures S5B–S5D). Therefore, these lines label two distinct cell types, PD2a1 (without calyx projections) and PD2b1 (with calyx projections). The calyx is the site of PN input to the MB, upstream of the site of associative olfactory memory, arguing against a role for this connection in our memory retrieval phenotype. Because we could not separately manipulate these two cell-types with our driver lines, we refer to them as PD2a1 and PD2b1. PD2a1 and PD2b1 neurons are morphologically similar to a large group of cells named ''type I'' (Fişek and Wilson, 2014).

MBON-α2sc Drives Activity in PD2a1 and PD2b1

Double labeling experiments suggested that MBON-α2sc is presynaptic to PD2a1 and PD2b1, but light microscopy does not have the resolution to confirm synaptic connectivity. We used GFP reconstitution across synaptic partners (GRASP) (Gordon and Scott, 2009) as a measure of the proximity of PD2a1 and PD2b1 dendrites and MBON axons. The experimental genotype displayed clear GFP reconstitution in the dorsal LH (Figure 3A), indicating that processes are close enough to form synapses; no signal was detected in control brains (Figures 3B and 3C).

Because MBON-α2sc is cholinergic (Aso et al., 2014a; Séjourné et al., 2011), we would expect that stimulating this neuron would drive activity in PD2a1 and PD2b1 if these neurons are connected. We expressed the heat-activated ion channel dTRPA1 (Hamada et al., 2008) in MBON-α2sc (Figures 3D–3F) while recording calcium transients in PD2a1 and PD2b1. We used R37G11-GAL4 to express GCaMP6f (Chen et al., 2013) and our R71D08-LexA line to drive dTRPA1 (Figure 3D). We imaged PD2a1 and PD2b1 axons *in vivo* to determine whether driving MBON-α2sc could induce calcium transients in PD2a1 and PD2b1. In a control experiment, we observed a small temperature-dependent increase in calcium in the absence of the LexAop2-dTRPA1 transgene, indicating that temperature alone weakly stimulates these neurons (Figures 3E and 3F). We also observed a small calcium increase in flies carrying only LexAop-dTRPA1 (Figures 3E and 3F). However, increasing temperature in flies expressing dTRPA1 in MBON-α2sc yielded a much larger calcium increase in calcium, indicating a functional connection (Figures 3E and 3F). We confirmed that dTRPA1 was

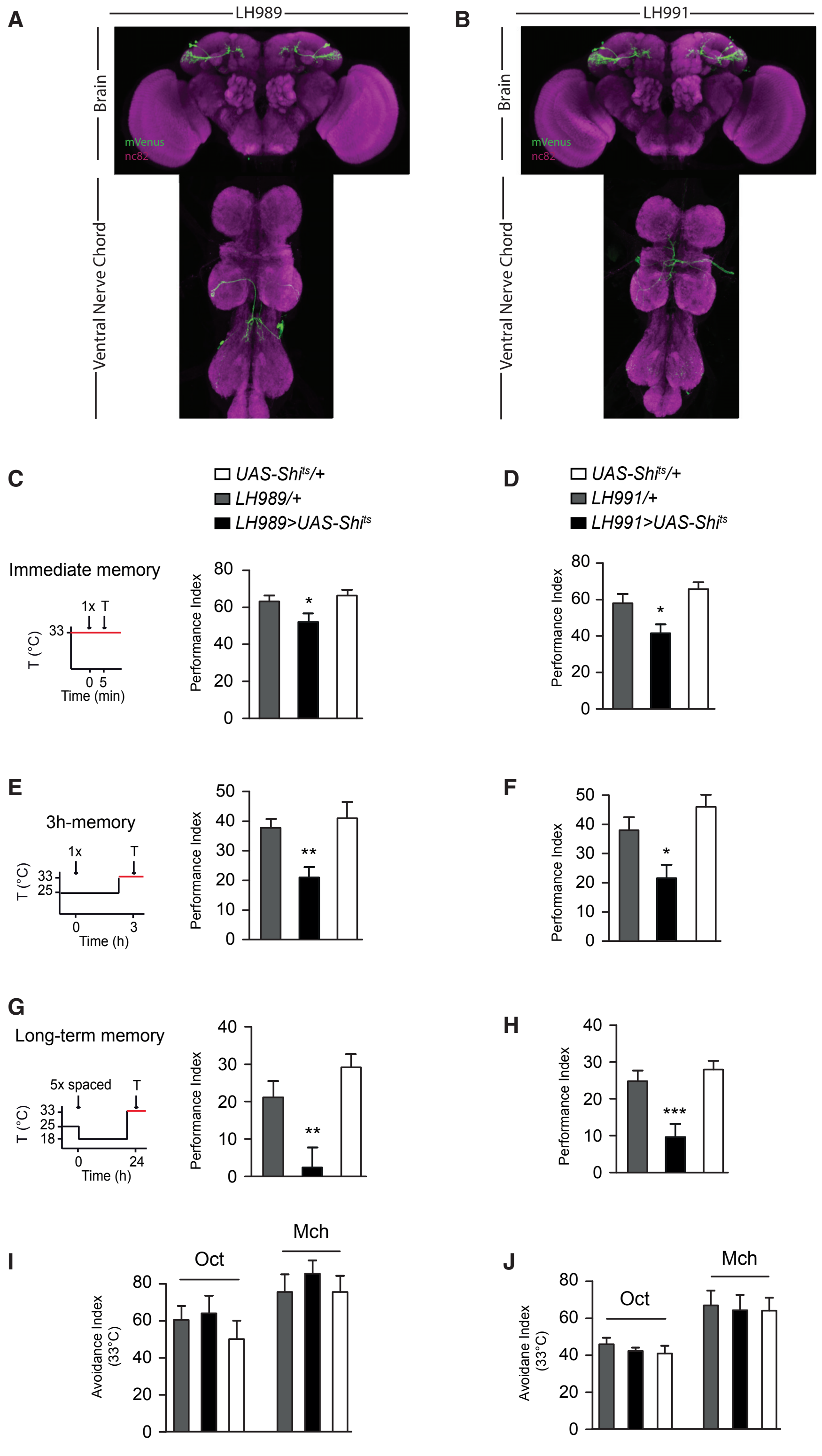

Figure 2. Specific Control with the Split-GAL4 System Confirms PD2a1 and PD2b1's Role in Memory Retrieval, but Not Innate Behavior

(A and B) Confocal z-projections of split-GAL4 lines targeting PD2a1 and PD2b1 neurons, LH989 (A) and LH991 (B). mVenus membrane stain, green; neuropil, magenta. Flies expressing Shi^{ts} by the split-GAL4 lines LH989 or LH991 were trained and tested according to the illustrated protocols along with genotypic controls (restrictive temperature in red).

(C and D) Silencing PD2a1 and PD2b1 neurons using LH989 (C; n = 14–15, $F_{(2,42)} = 4.13$, $p = 0.02$) or LH991 (D; n = 18, $F_{(2,53)} = 7.27$, $p = 0.0017$) impaired immediate memory after single-cycle training.

(E and F) Silencing PD2a1 and PD2b1 neurons during the retrieval phase 3 hr after single-cycle training using LH989 (E; n = 14, $F_{(2,42)} = 6.73$, $p = 0.0031$) or LH991 (F; n = 11–13, $F_{(2,35)} = 8.23$, $p = 0.0013$) caused a memory defect.

(G and H) Silencing PD2a1 and PD2b1 neurons during the retrieval phase 24 hr after spaced training using LH989 (G; n = 7–9, $F_{(2,23)} = 9.79$, $p = 0.0010$) or LH991 (H; n = 19–23, $F_{(2,72)} = 10.83$, $p < 0.0001$) abolished performance.

(I and J) Silencing PD2a1 and PD2b1 neurons using LH989 (I; Oct, n = 8–12, $F_{(2,29)} = 0.63$, $p = 0.54$; Mch, n = 10, $F_{(2,29)} = 0.44$, $p = 0.65$) or LH991 (J; Oct, n = 7–8, $F_{(2,22)} = 0.25$, $p = 0.78$; Mch, n = 7, $F_{(2,20)} = 0.068$, $p = 0.93$) had no effect on naive avoidance of Oct or Mch.

$^{*}p < 0.05$, $^{**}p < 0.01$, $^{***}p < 0.001$. Data are presented as mean ± SEM. See also Figures S4 and S5.

expressed in MBON-α2sc by expressing a LexAop2-TdTomato reporter in the same landing site as the LexAop2-dTRPA1 transgene (Figure S5E). These thermogenetic activation data, together with the double labeling and GRASP results, suggest that MBON-α2sc connects to the PD2a1 and PD2b1 LH cell type necessary for memory retrieval.

Synaptic Resolution Analysis of MBON-α2sc and PD2a1 and PD2b1 Connectivity

A GRASP signal indicates that PD2a1 and PD2b1 dendrites and MBON-α2sc axons are in close proximity but does not demonstrate the existence of synapses. We therefore leveraged a new whole female brain serial section electron microscopy (EM) volume (Saalfeld et al., 2009; Zheng et al., 2018) to study connectivity with synaptic resolution. We first identified the single MBON-α2sc with a soma and dendrite in the right hemisphere of this volume by tracing downstream of Kenyon cells in the MB α2 compartment. We then used NBLAST combined with light EM bridging registrations to match its backbone structure with light-level image data (Costa et al., 2016; Zheng et al., 2018; Figures 4A and 4A'). We repeated this procedure to identify the

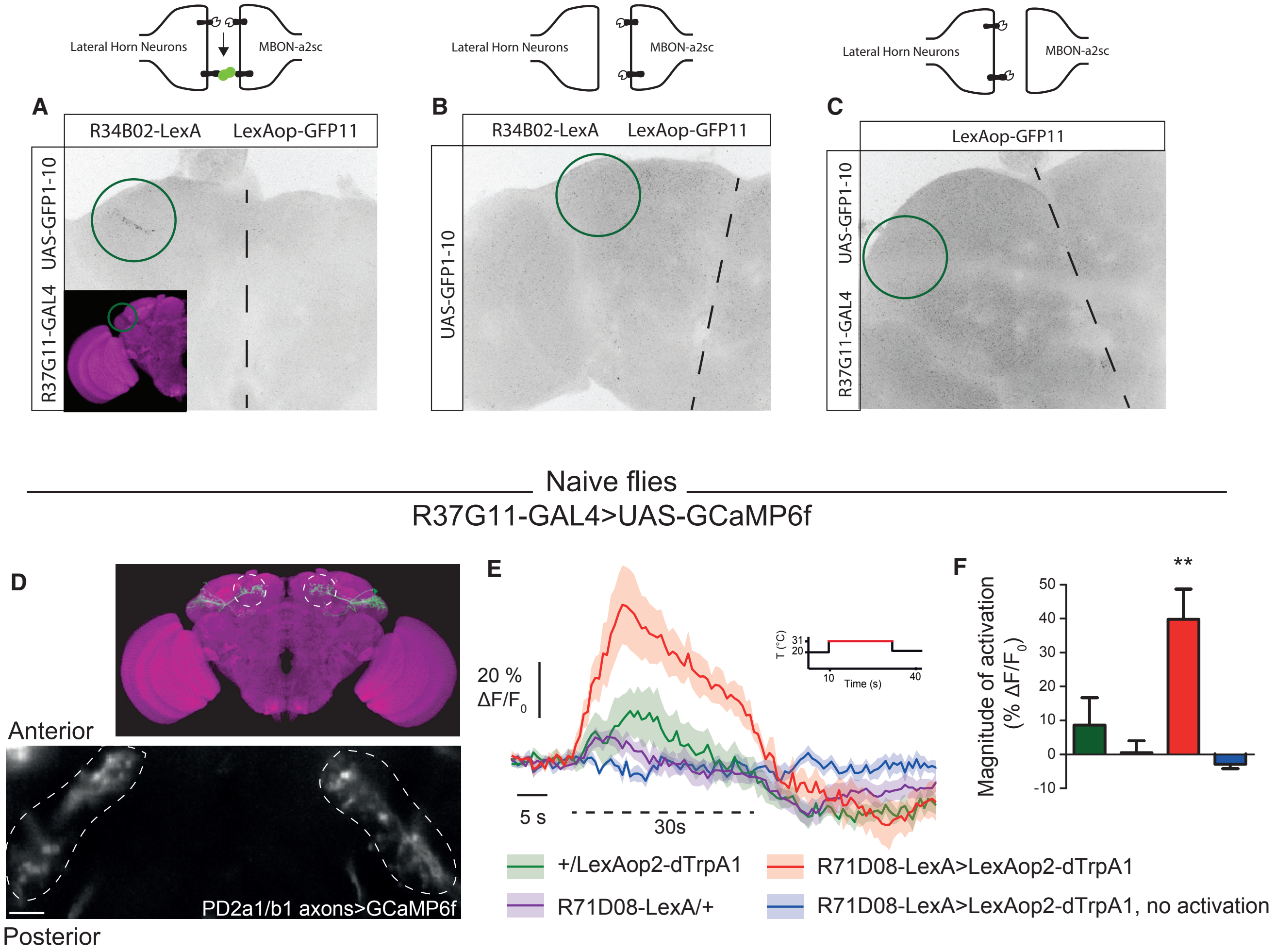

Figure 3. MBON-α2sc Is Functionally Connected to PD2a1 and PD2b1

(A–C) GRASP signal in the dorsal LH (green circles, dashed lines indicate midlines) for the experimental genotype (A) and two controls (B and C). Genotypes and controls are represented in the schematics above each figure. Images are representative of n = 3.

(D) GCaMP6f was expressed in PD2a1 and PD2b1 neurons with the R37G11-GAL4 driver (scale bar, 10 μm). Fluorescence was recorded *in vivo* from the axonal compartment of PD2a1 and PD2b1 neurons while the temperature was shifted from 20°C to 31°C (dashed line on F, except for the blue trace).

(E) The calcium increase of PD2a1 and PD2b1 neurons because of thermal activation of V2 MBONs (red trace) was stronger than that because of temperature shift only in the genotypic controls (green and purple traces).

(F) Quantification of calcium increase from the traces (n = 10 flies per condition, except 71D08-LexA/+ [n = 8], $F_{(3,37)} = 9.09$, $p = 0.0001$).

**$p < 0.01$. Data are presented as mean ± SEM. See also Figure S6.

contralateral (left) MBON-α2sc because their axons project bilaterally to both LHs.

We reconstructed the right LH axonal arbors to completion for both MBON-α2sc neurons, marking pre- and postsynapses, and annotating the connections each presynapse makes in the right LH (Figure 4A’). We identified 183 and 190 presynapses for the left and right MBON-α2sc, respectively, in the right LH (Figure 4A’). Each individual presynapse was polyadic, connecting to 7.8 ± 4.6 (mean ± SD) postsynaptic targets. We sampled 25% of these connections (Figure 4A’’, inset) and identified 70 large target arbors (>300 μm of neuronal cable; data not shown), each likely belonging to different neurons. We found that two of these target neurons had the distinctive morphology of the PD2a1 and PD2b1 cells. Based on these two candidate cells, we located the PD2 primary neurite tract (purple dots in Figure S5F) and coarsely reconstructed all neurons in this tract (Figure S5F) to identify a total of five PD2a1 (PD2a1#1–5) and two PD2b1 (PD2b#1–2) cells (Figures 4B and 4B’; STAR Methods). Comparison of MCFO and EM data confirmed the identity of PD2a1 and PD2b1 neurons (Figures 4B’ and S5G). This was corroborated by NBLAST cluster analysis, indicating no clear separation between EM, FlyCircuit (Chiang et al., 2011), and MCFO data (Figure S5H). PD2a1 dendritic arbors contained some presynapses in the LH but at lower density than their axons. For both PD2b1 neurons, the LH and calycal projections were exclusively post-synaptic (Figures 4B’ and

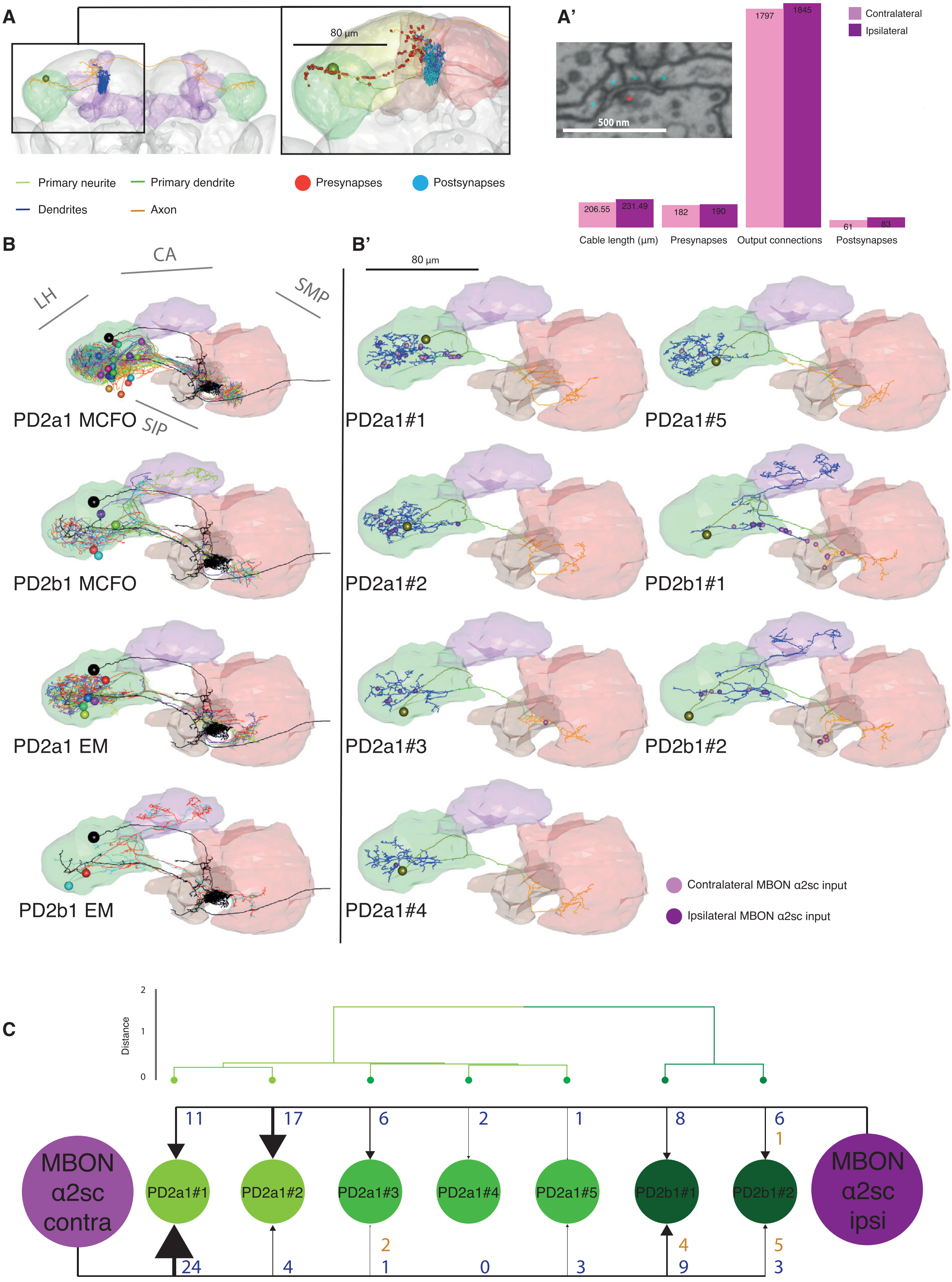

(legend on next page)

S6D). We confirmed the existence of these two types of neurons by clustering NBLAST scores derived from dendritic and axonal compartments, which yielded two distinct groups for PD2a1 and PD2b1 (Figure 4C). PD2a1 neurons could be further subdivided into two groups, one of which (PD2a1#1 and PD2a1#2) received greater MBON input per neuron (Figure 4C). Consistent with observations in the larva (Schneider-Mizell et al., 2016), the vast majority of postsynapses were found on microtubule-free lower-order branches (Figure S6C). Summary data for pre- and postsynaptic sites, in addition to cable length for MBON-α2sc and PD2a1 and PD2b1, is presented in Figure S6. PD2a1 and PD2b1 presynapses contained only clear-core vesicles, suggesting that they do not release catecholamine or peptide neurotransmitters (data not shown).

All PD2a1 and PD2b1 cells received input from the ipsilateral MBON-α2sc axon, and most received input from both MBONs (Figure 4C). In sum, these observations confirm that PD2a1 and PD2b1 neurons are a direct synaptic partner of MBON-α2sc in the LH.

PD2a1 and PD2b1 Neurons Have Decreased Responses to the CS+

After training, MBON-α2sc depresses its response to the CS (Hige et al., 2015a; Séjourné et al., 2011). We next examined whether PD2a1 and PD2b1 neurons downstream of MBON-α2sc also modulate their response to the CS+ odor. We expressed the GCaMP3 calcium indicator (Tian et al., 2009) in PD2a1 and PD2b1 (Figure 5A). In naive flies, PD2a1 and PD2b1 neurons responded to 3-octanol (Oct) and 4-methylcyclohexanol (Mch), the two odorants alternately used as CS+ in our behavioral experiments (Figures 1D, 1E, and 2).

We next looked for training-induced changes in odor responses, comparing PD2a1 and PD2b1 responses following either associative training or a control, unpaired protocol that matched the odor sequence of the associative training, but temporally separated electric shock and odor delivery (see Figure S7 for the protocol). We performed these experiments either 3 hr after single-cycle training (Figures S7A and S7B) or 24 hr after spaced training (Figure S7C), using either Oct or Mch as the CS+. We found that pairing CS+ and electric shock during single-cycle training resulted in a decreased CS+ response in PD2a1 and PD2b1 axons 3 hr later, either compared with unpaired controls (Figures 5D' and 5E') or the CS− response in the same fly (Figures 5D'' and 5E''). Similar results were observed 24 hr after spaced training (Figures 5F and 5G). These data suggest that PD2a1 and PD2b1 neurons receive memory-relevant information (the decreased CS+ response), resulting from depression at Kenyon cell to MBON-α2sc synapses.

PD2a1 and PD2b1 Also Receive Input from Uniglomerular PNs Encoding Attractive Odors

PD2a1 and PD2b1 dendrites in the LH are poised to receive input from PNs as well as MBON-α2sc. Antennal lobe PNs have been identified in the EM volume (Zheng et al., 2018), enabling us to identify the specific input from each AL glomerulus to PD2a1 and PD2b1 dendrites in the LH and calyx (Figure 6A'). We annotated LH presynapses for each uniglomerular excitatory mALT PN (n = 112 PNs, 51 glomeruli; R.J.V.R., P.S., A.S.B., D.B., G.S.X.E.J., and S. Lauritzen, unpublished data). Most PD2a1 and PD2b1 neurons received synaptic input from several glomeruli, chiefly DM1, DP1m, DM4, VA2, DP1l, and VM3 (Figure 6A), although some differences were observed across cells.

To better understand this connectivity matrix, we annotated the behavioral function of input PNs according to published studies. The dorsal LH, where PD2a1 and PD2b1 dendrites are located, has been associated with coding of food odors (Jefferis et al., 2007). Consistent with this, the top synaptically connected glomeruli (DM1, DP1m, DM4, and VA2) are responsive to appetitive and food odors (Badel et al., 2016; Knaden et al., 2012; Semmelhack and Wang, 2009; Figure 6A), indicating that PD2a1 and PD2b1 receives direct PN input mostly from appetitive olfactory channels. Furthermore, input to both DM1 and VA2 glomeruli is required for approach behavior to vinegar in hungry flies (Semmelhack and Wang, 2009).

PD2b1 cells have a dendritic branch in the MB calyx. We found that these dendrites' largest inputs are from the same top four glomeruli (DM1, DP1m, DM4, and VA2) that target PD2a1 and PD2b1 dendrites in the LH (Figure 6A). This is even true for PD2b1#1, a cell that receives negligible DP1m and DM4 input in the LH but many synapses from these PNs in the calyx (Figure 6A').

Uniglomerular PNs provide 36% of the total inputs to PD2a1 and PD2b1 dendrites in the LH, whereas MBON-α2sc contributes 2.5% on average (Figure 6A'). This varies across individual neurons, with some PD2a1 and PD2b1 neurons receiving up to 15% of their known excitatory input from MBON-α2sc

Figure 4. Electron Microscopy Reconstruction of PD2a1 and PD2b1

(A) Reconstruction of the right-side MBON-α2sc in a whole brain EM volume. The cell body is represented as a sphere, and the primary neurite (yellow-green), primary dendrite (green), dendrite (blue), and axon (orange) compartments are separately colored. Neuropils: LH in green, MB in purple. Inset: position of presynapses (red spheres) and postsynapses (cyan spheres) on the right-side MBON-α2sc. Neuropils: SLP in yellow, SIP in orange, SMP in red.

(A') Comparison of different metrics for the reconstructions of the contralateral and ipsilateral MBON-α2sc within the LH (green in A). Inset: example of a polyadic synapse with a single T-bar (red dot) and multiple postsynapses (blue dots), referred to as "output connections" in the bar chart. Scale bar, 500 nm.

(B) Dorsal view of co-registered PD2a1 and PD2b1 MCFO data (top two panels, respectively) and EM reconstructions (bottom two panels, respectively). Cells are individually colored. Ipsilateral MBON-α2sc is shown in black.

(B') Dorsal view of single PD2a1 and PD2b1 neurons reconstructed in the EM volume. Yellow-green spheres represent somata, whereas ipsilateral and contralateral MBON-α2sc synaptic connections are represented in dark and light purple, respectively.

(C) Schematic of synaptic connectivity from the two MBON-α2sc neurons onto each PD2a1 and PD2b1 cell. The PD2a1 and PD2b1 cells are clustered according to the NBLAST score of their axons and dendrites, identifying two main groups, PD2a1 and PD2b1. Numbers beside each arrow indicate the number of outgoing connections made onto PD2a1 and PD2b1 neurons dendro-dendritically (blue) and axo-axonically (orange). Contra, contralateral; ipsi, ipsilateral; LH, lateral horn; CA, mushroom body calyx; SIP, superior intermediate protocerebrum; SLP, superior lateral protocerebrum; SMP, superior medial protocerebrum.

See also Figures S7 and S8.

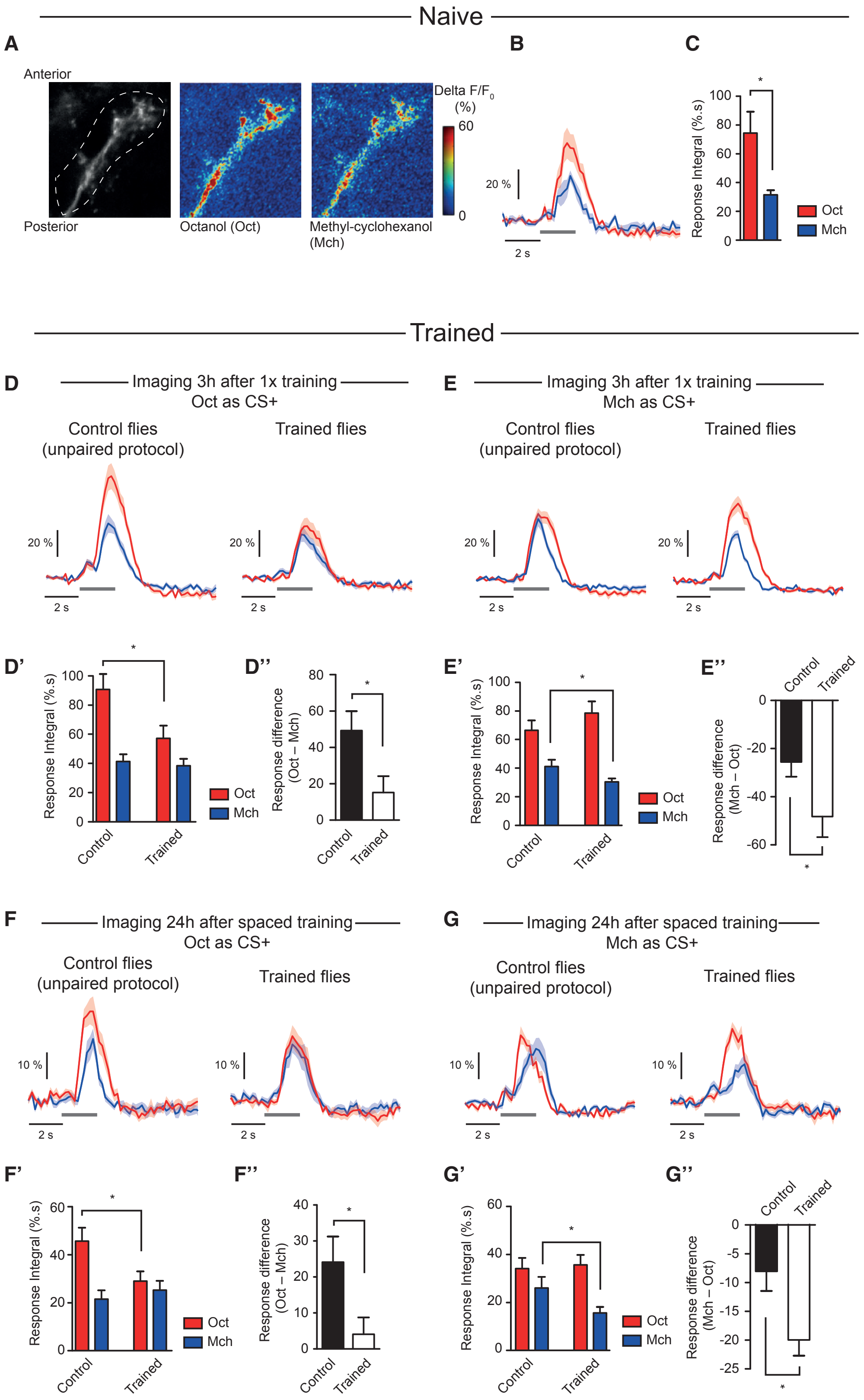

(legend on next page)

(Figure 6A'; see below). To compare the significance of direct MBON to LH output neuron (LHON) connectivity with other dendritic input, we traced every neuron upstream of PD2a1#1's 732 LH postsynapses to identification. All but 4 synapses could be matched to one of 165 partner neurons, which we divided into 6 major groups (Figure 6B).

We found that PNs and MBON-α2sc provided 26.5% and 4.6% of the dendritic input, respectively. The great majority of the remaining input originated from within the LH (either local neurons, 33%, or reciprocal synapses from LH output neurons, 18.4%). There was also a small group of inhibitory PN connections (1.9%). The remaining 15.6% of input was from previously undescribed neuronal classes originating from the rest of the protocerebrum; we do not know whether these are inhibitory or excitatory. From these results, we can conclude that MBON-α2sc provides between 9.8% and 14.7% of the direct excitation to this PD2a1 neuron and is the fourth largest input. Therefore, together, uniglomerular PNs and MBON-α2sc provide the large majority of the driving cholinergic input to PD2a1 and PD2b1.

PD2a1 and PD2b1 Integrate Input from MBON-α2sc and PNs during Olfactory Stimulation

Our anatomical data indicate that PD2a1 and PD2b1 integrate olfactory information from the very broadly tuned MBON-α2sc and PNs encoding food odors. To directly measure the olfactory tuning of PD2a1 and PD2b1 neurons, we performed whole-cell electrophysiology, which is more sensitive than calcium imaging. We targeted GFP-labeled PD2a1 and PD2b1 neurons for *in vivo* recording, followed by stimulation with a large battery of different odorants (Figure 6C; STAR Methods). As expected, we found that PD2a1 and PD2b1 neurons were broadly tuned, responding to almost all odors at the test concentrations (Figure 6C'). Response variability was not noticeably greater than other LH neurons (Frechter et al., 2018). Apple cider vinegar drove the highest response, consistent with strong DM1 and/or VA2 inputs identified by EM. Although most other strong responses were to appetitive odors, benzaldehyde, which is innately aversive, drove the second highest response. We do note that benzaldehyde is also sensed through a non-olfactory pathway (Keene et al., 2004) that could act via the LH or MB, complicating interpretation. The conditioning odors Mch and Oct, which are naively aversive (Séjourné et al., 2011; Tully et al., 1994), elicited intermediate responses.

One explanation for this broad PD2a1 and PD2b1 odor tuning is that PD2a1 and PD2b1 integrates direct PN input that is relatively tuned to food odors together with broad, odor non-specific input from MBON-α2sc. We know that artificial MBON-α2sc stimulation can drive PD2a1 and PD2b1 calcium responses (Figures 3D–3F); is this connection strong enough to have an effect on more naturalistic activity?

We designed an experiment to test the effect of MBON-α2sc on odor-evoked activity and to provide functional evidence that PD2a1 and PD2b1 indeed integrates both direct AL input from PNs as well as indirect input from the MB. We reversibly silenced MBON-α2sc neurotransmission with shibire^{ts1} while imaging PD2a1 and PD2b1 calcium odor responses *in vivo* (Figure 6D). Silencing MBON-α2sc strongly attenuated PD2a1 and PD2b1 responses to both Mch and Oct (Figures 6E and 6F) compared with genotype controls (Figures S8A and S8B), indicating that MBON depression can significantly reduce PD2a1 and PD2b1 responses to our training odors.

Because both Oct and Mch are innately aversive, we tested the effect of MBON-α2sc signaling on responses to apple cider vinegar (ACV). Silencing MBON-α2sc had no effect on PD2a1 and PD2b1 responses to apple cider vinegar (Figure 6G; see Figure S8C for the genotypic control). This is likely because apple cider vinegar very strongly activates the major PNs upstream of PD2a1 and PD2b1 neurons, reducing the effect of MBON-α2sc on PD2a1 and PD2b1 coding. We also tested two attractive monomolecular odorants, ethyl acetate and isoamyl acetate. We again found that silencing MBON-α2sc attenuated odor responses in PD2a1 and PD2b1 (Figures 6H and 6I; see Figure S8D for the genotypic control). These results confirm that PD2a1 and PD2b1 integrate input from PNs and MBON-α2sc. They also show that direct (PN) and indirect (MBON) pathways have different relative strengths for different odors.

PD2a1 and PD2b1 Neurons Are Required for Olfactory Approach Behavior

Our functional and behavioral data demonstrate that PD2a1 and PD2b1 are modulated by and necessary for aversive olfactory

Figure 5. PD2a1 and PD2b1 Decrease Response to the CS+ after Training

(A) GCaMP3 was expressed in PD2a1 and PD2b1 with R37G11-GAL4. Olfactory responses to Oct and Mch were recorded *in vivo* from the axonal compartment of PD2a1 and b1 neurons.

(B and C) In naive flies, the calcium increase in PD2a1 and PD2b1 neurons in response to Oct was larger than Mch (average traces from n = 6 flies; t test, p = 0.015; B, average time trace; C, bar chart of response integral).

(D–D'') Odor responses were recorded 3 hr after single-cycle training using Oct as CS+ (n = 19 flies) or after the corresponding unpaired control protocol (n = 20 flies) (Figure S9A).The integral of the odor responses (D'; t test, p = 0.023) and the calculation of the difference between Oct and Mch responses (D''; t test, p = 0.024) revealed a decreased response to the CS+ after the associative protocol.

(E–E'') Odor responses were recorded 3 hr after single-cycle training using Mch as CS+ (n = 22 flies) or after the corresponding unpaired control protocol (n = 21 flies) (Figure S9B). The integral of the odor responses (E'; p = 0.047) and the calculation of the difference between Mch and Oct responses (E''; t test, p = 0.041) revealed a decreased response to the CS+ after the associative protocol.

(F–F'') Odor responses were recorded 24 hr after spaced training using Oct as CS+ (n = 9 flies) or after the corresponding unpaired control protocol (n = 11 flies) (Figure S9C). The integral of the odor responses (F'; t test, p = 0.036) and the calculation of the difference between Oct and Mch responses (F''; t test, p = 0.035) revealed a decreased response to the CS+ after the associative protocol.

(G–G'') Odor responses were recorded 24 hr after spaced training using Mch as CS+ (n = 9 flies) or after the corresponding unpaired control protocol (n = 9 flies) (Figure S9C). The integral of the odor responses (G'; t test, p = 0.047) and the calculation of the difference between Mch and Oct responses (G''; t test, p = 0.010) revealed a decreased response to the CS+ after the associative protocol.

*p < 0.05. Data are presented as mean ± SEM. Gray bars indicate periods of olfactory stimulation. See also Figure S9.

CellPress

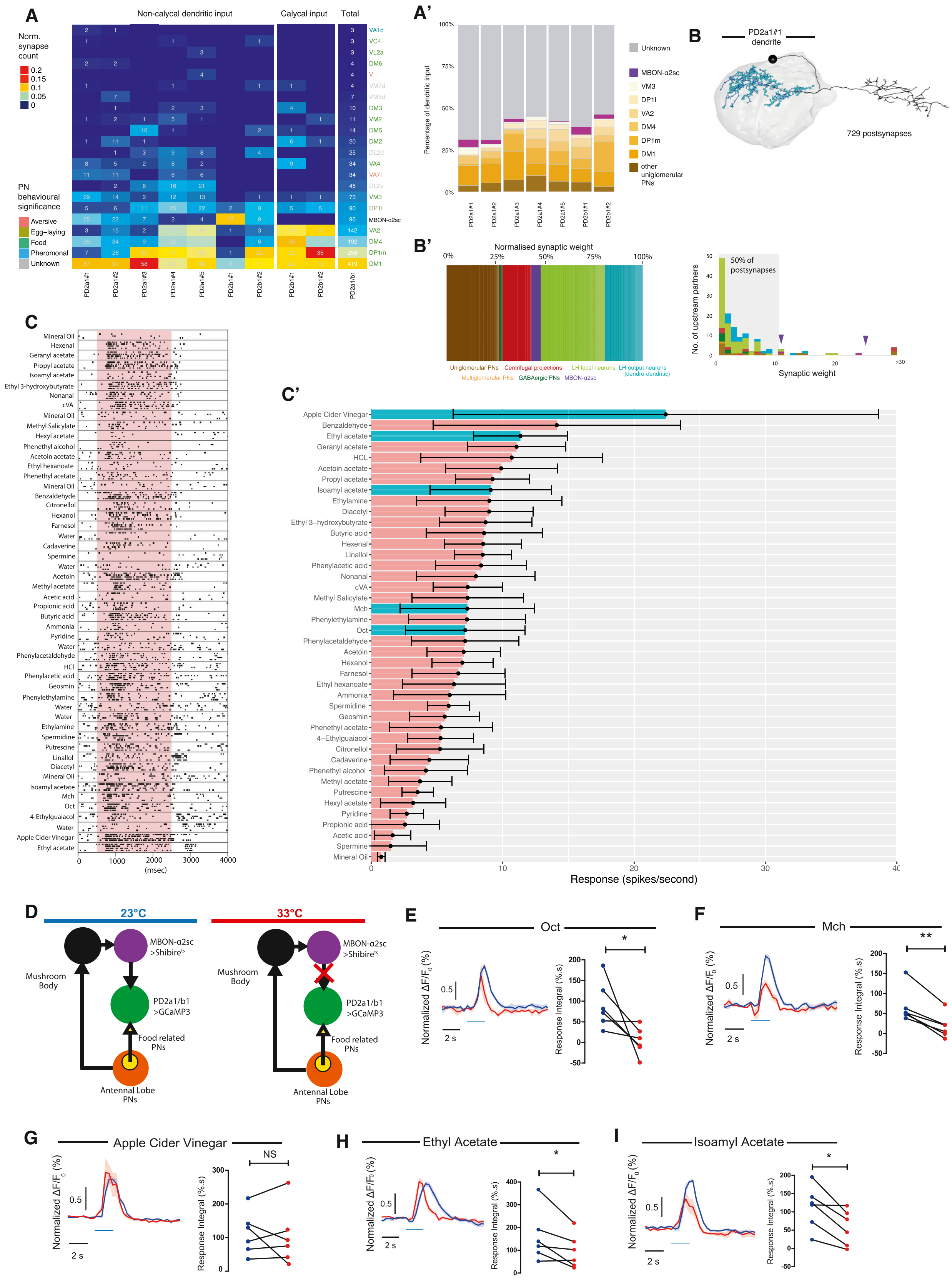

(legend on next page)

memory retrieval. However, our EM reconstruction and electrophysiological characterization revealed that these neurons respond strongly to apple cider vinegar, an appetitive odor. This suggests that PD2a1 and PD2b1 neurons may mediate innate olfactory attraction. To test whether these neurons are necessary for approach behavior, we silenced PD2a1 and PD2b1 neurons in naive, starved animals, for which apple cider vinegar is an appetitive stimulus (Semmelhack and Wang, 2009). Silencing PD2a1 and PD2b1 neurons completely abolished vinegar attraction compared with the genotype controls (Figure 7A). At the permissive temperature, no difference was observed in the behavior of experimental and control genotypes (Figure S8E). To determine whether PD2a1 and PD2b1 was necessary for approach to other odors, we used ethyl acetate and isoamyl acetate, both of which are monomolecular, attractive odors. We found that PD2a1 and PD2b1 neurotransmission was required for attraction to ethyl acetate (Figure 7B; see Figure S8F for permissive temperature controls) but dispensable for approach to isoamyl acetate (Figure 7C). This odor specificity is likely a combination of two factors. First, the PNs providing direct input to PD2a1 and PD2b1 appear more responsive to ethyl acetate than isoamyl acetate (Badel et al., 2016). Second, there are likely additional LH neurons that promote attraction, including neurons that receive PN inputs that are selective for isoamyl acetate over ethyl acetate.

A Model for Memory Retrieval by MBON-α2sc Modulation of PD2a1 and PD2b1

Our results indicate that PD2a1 and PD2b1 neurons play a dual role in olfaction; they are necessary for both aversive memory retrieval and innate olfactory attraction. We show (anatomically) that PD2a1 and PD2b1 receives direct appetitive odor information from the AL and provide anatomical and functional evidence for a pathway from the MB to the LH that is depressed after learning. Together, these data led us to propose a circuit model for memory retrieval in our assay (Figures 7D and 7E), based on integration of innate and learned sensory representations by PD2a1 and PD2b1 neurons.

In naive animals, PD2a1 and PD2b1 integrates innate and learned olfactory representations and interfaces with approach circuitry (Figure 7D). After aversive olfactory conditioning, MBON-α2sc depresses its response to the trained odor, which results in a reduced excitatory drive to PD2a1 and PD2b1 during CS+ sensation relative to naive animals (Figure 5). Because PD2a1 and PD2b1 are cholinergic (Figure 1G), this depression results in decreased stimulation of downstream partners of PD2a1 and PD2b1 that mediate approach. This depression reduces the attractive bias to the CS+, leading to net avoidance of the trained odor (Figure 7E). Our experiments used a T maze memory paradigm, where flies choose between two arms containing odors that are initially of similar valence; after training, a

Figure 6. PD2a1 and PD2b1 Receive Input from Appetitive PNs and Are Broadly Tuned

(A) Summary heatmap of antennal lobe glomeruli with uniglomerular, excitatory PN connectivity to individual PD2a1 and PD2b1 neurons as determined by EM reconstruction. The connectivity heatmap is separated by neuropil location: PD2a1 and PD2b1 LH dendrites, PD2b1 MB calyx dendrites and total across all PD2a1 and PD2b1 dendrites. Cell numbers represent the number of synapses, and heatmap coloring represents the synapse count normalized by the total number of postsynapses in that neuropil. Uniglomerular PNs with no connectivity are not shown. Uniglomerular PNs from connected glomeruli or MBON-α2sc are ordered by connection strength. PN names are colored by their behavioral significance based on published studies.

(A') The number of synaptic inputs for all PD2a1 and PD2b1 dendrites traced in this study. Input is either undefined (gray), uniglomerular PN (oranges), or MBON-α2sc (purple).

(B) Reconstruction of all presynaptic partners to PD2a1#1 in the EM volume. Shown is the PD2a1#1 EM-reconstructed skeleton with dendritic postsynapses highlighted in blue.

(B') Right: stacked bar chart showing the percentages of postsynapses contributed by different types of input neurons (different colors). Left: histogram showing the number of upstream postsynpatic partners against their synaptic weight (number of synapses onto PD2a1#1). The gray box highlights that 50% of PD2a1#1's postsynapses are spent on neurons that only input PD2a1#1 by less than 10 synaptic connections. MBON-α2sc is indicated by purple arrowheads.

(C) Electrophysiological recording raster plot from a representative PD2a1 neuron. The responses of each cell to the different odors are stacked, black squares represent action potentials, and there are 4 presentations of each odor. The red block represents the odor stimulation period.

(C') Tuning curve of PD2a1 and PD2b1 neurons. Responses are shown in hertz. Data are mean ± SEM; n = 7 cells, consisting of one PD2b1, one PD2a1 or PD2b1, and five PD2a1 neurons. Odors in the text are shown in cyan.

(D) Schematic for imaging experiments with MBON-α2sc silencing. Flies express Shi^{ts} in MBON-α2sc and GCaMP3 in PD2a1 and PD2b1 neurons for calcium imaging. At the permissive temperature (left), there is no effect on MBON-α2sc neurotransmission, and PD2a1 and PD2b1 neurons receive input from both MBON-α2sc and directly from the antennal lobe. MBON-α2sc is silenced at the restrictive temperature (right), although the PD2a1 and PD2b1 neurons still receive input from the antennal lobe.

(E) Response of PD2a1 and PD2b1 axons to Oct with or without MBON-α2sc silencing. Left: time traces of normalized GCaMP3 fluorescence (STAR Methods) are shown at permissive (blue) and restrictive (red) temperature in response to Oct stimulation (light blue bar). Right: the integral of the absolute odor responses for each fly at the permissive (blue) and restrictive (red) temperatures are plotted, which revealed decreased response to Oct after MBON-α2sc silencing (n = 6, paired t test = 0.044).

(F) Response of PD2a1 and PD2b1 axons to Mch with or without MBON-α2sc silencing. The layout of the data is the same as in (E). This revealed a decreased response to Mch after MBON-α2sc silencing (n = 6, paired t test = 0.0015).

(G) Response of PD2a1 and PD2b1 axons to vinegar with or without MBON-α2sc silencing. The layout of the data is the same as in (E). There was no change in response to vinegar after MBON-α2sc silencing (n = 6, paired t test = 0.67).

(H) Response of PD2a1 and PD2b1 axons to ethyl acetate with or without MBON-α2sc silencing. The layout of the data is the same as in (E). This revealed a decreased response to ethyl acetate after MBON-α2sc silencing (n = 6, paired t test = 0.039).

(I) Response of PD2a1 and PD2b1 axons to isoamyl acetate with or without MBON-α2sc silencing. This revealed a decreased response to isoamyl acetate after MBON-α2sc silencing (n = 6, paired t test = 0.012).

*$p < 0.05$, **$p < 0.01$. See also Figure S10.

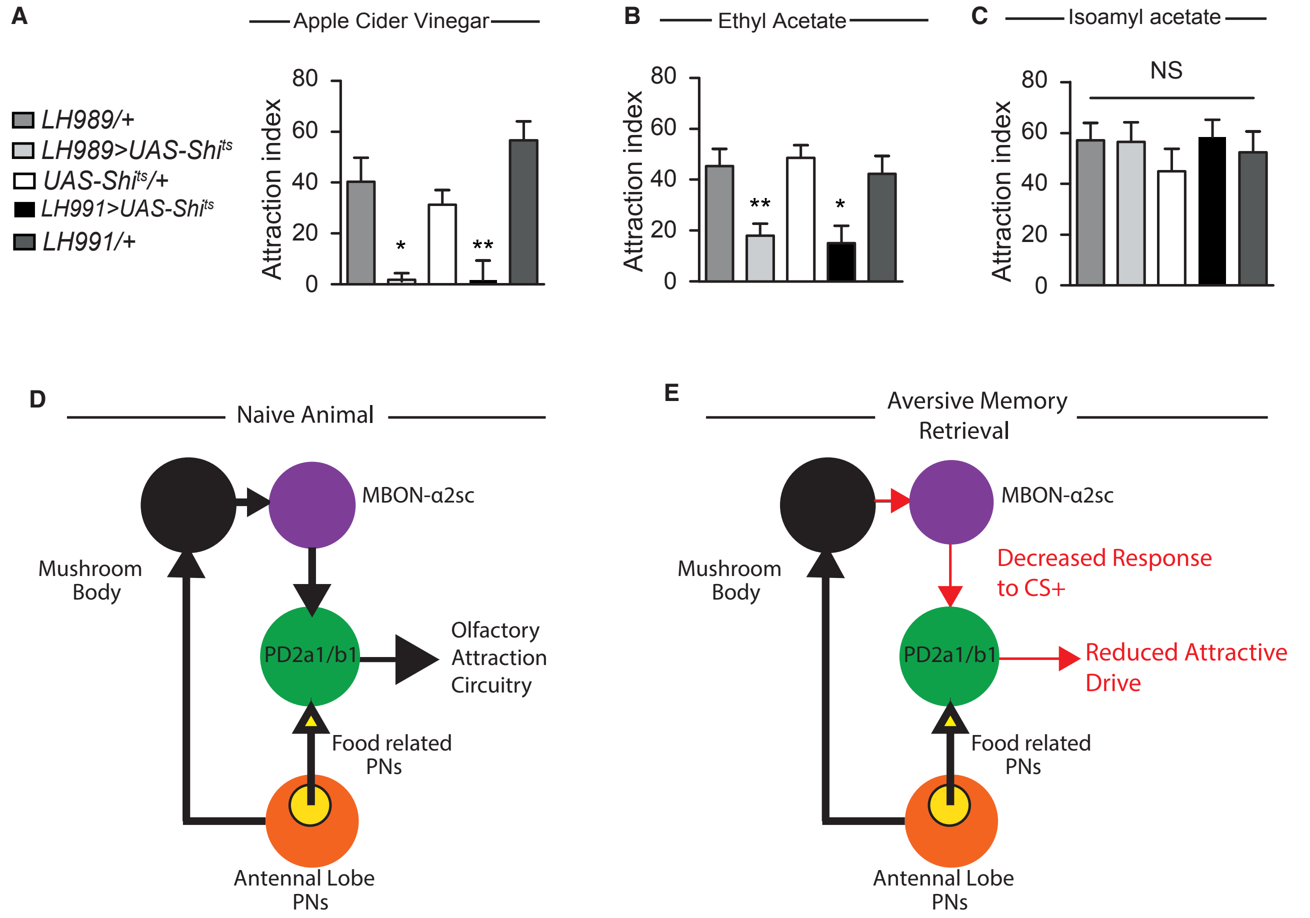

Figure 7. PD2a1 and PD2b1 Mediate Innate Olfactory Attraction, Leading to a Model of Aversive Memory Retrieval

(A) Flies expressing Shi^{ts} driven by either LH989 or LH991 showed impaired attraction to apple cider vinegar relative to their genotype controls at the restrictive temperature ($n = 9$, $F_{(4,44)} = 12.10$, $p < 0.0001$).

(B) Flies expressing Shi^{ts} driven by either LH989 or LH991 showed impaired attraction to ethyl acetate relative to their genotype controls at the restrictive temperature ($n = 13\text{-}16$, $F_{(4,73)} = 6.34$, $p = 0.0002$).

(C) Flies expressing Shi^{ts} driven by either LH989 or LH991 showed impaired attraction to isoamyl acetate relative to their genotype controls at the restrictive temperature ($n = 8\text{-}9$, $F_{(4,42)} = 0.53$, $p = 0.72$).

(D and E) Model for how PD2a1 and PD2b1 functions in naive and trained animals.

(D) In naive animals, PD2a1 and PD2b1 receives input from both the MB (black sphere, via broadly tuned MBON-α2sc) and directly from the AL food-related PNs (yellow sphere within AL). PD2a1 and PD2b1 activity is necessary for approach behavior to some olfactory stimuli.

(E) After conditioning, the response of MBON-α2sc to the CS+ is reduced via synaptic depression at the MB-to-MBON synapse. This results in a decreased response to the CS+ in PD2a1 and PD2b1. Because PD2a1 and PD2b1 are cholinergic and excitatory, this reduces the input onto downstream approach circuits, resulting in decreased attraction to the CS+ during memory recall.

*$p < 0.05$, **$p < 0.01$. See also Figure S10.

relatively small decrement in the appetitive drive in the CS+ arm should be sufficient to bias flies to choose the CS− arm.

PD2a1 and PD2b1 Interdigitates with DAN Dendrites and MBON Axons in MB Convergence Zones

To obtain some initial clues regarding how PD2a1 and PD2b1 neurons mediate olfactory attraction, we identified potential downstream targets of this LH cell type. Light and EM characterization of PD2a1 and PD2b1 axons suggested that they transmit information from the LH to the crepine (CRE), superior medial protocerebrum (SMP), and SIP (Figure S6). The CRE, SMP, and SIP have been identified as convergence zones for the dendrites of DANs and MBON axons (Aso et al., 2014a, 2014b; Owald et al., 2015). This raises the possibility that PD2a1 and PD2b1 may interact with input and output neurons of the MB assembly that drive valence behavior (Aso et al., 2014b).

We searched for potential contact sites by computational alignment of light microscopy data, generating a percentage overlap score of PD2 with DAN dendrites (Figure 8A) or MBON axons (Figure 8C). We investigated all neurons with more than 15% overlap in this coarse analysis, using double labeling with

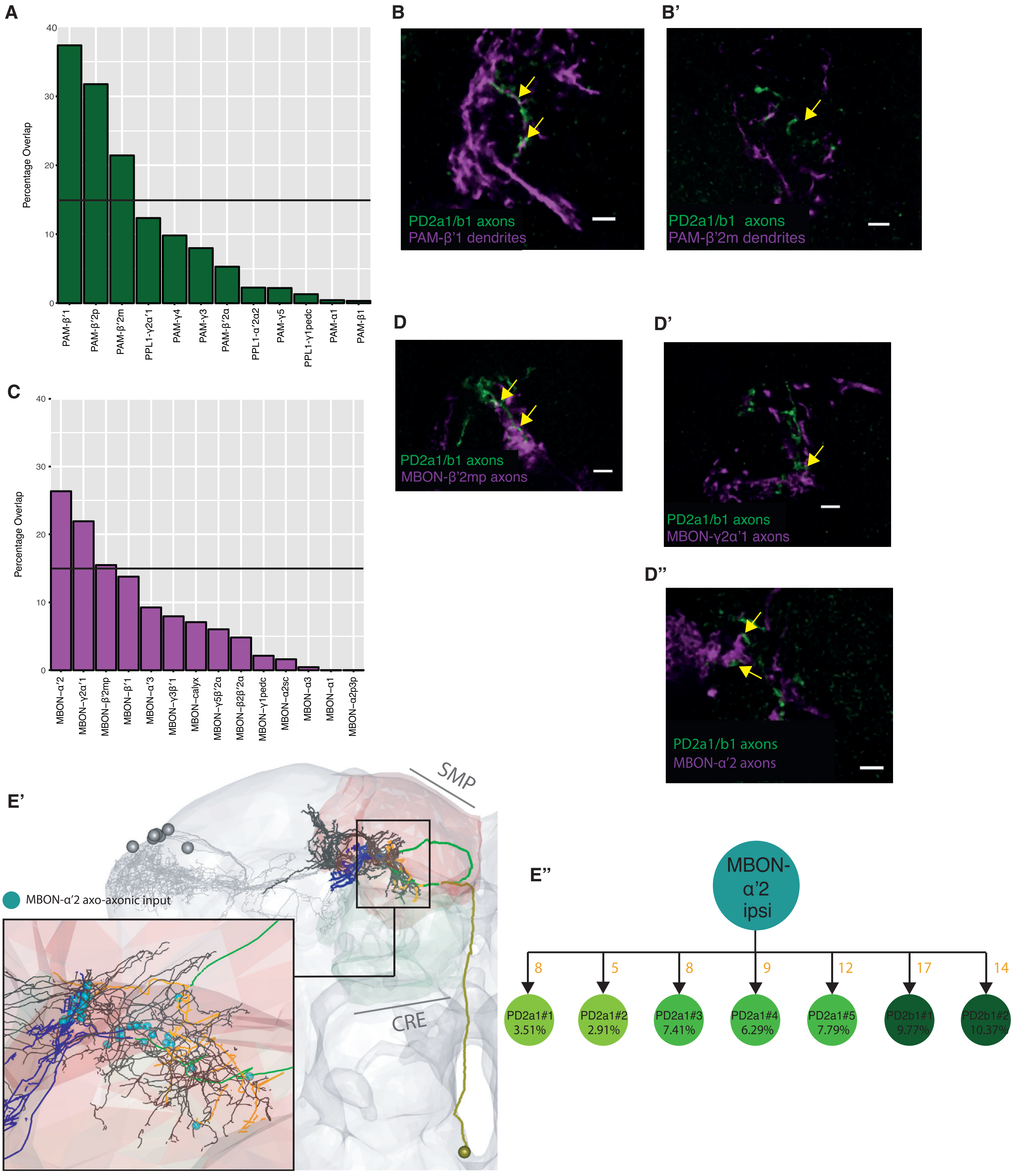

Figure 8. PD2a1 and PD2b1 Axons Interdigitate and Interact with DAN Dendrites and MBON Axons

(A) Histogram of light microscopy overlap between a mask of PD2a1 and PD2b1 axons and masks of the dendrites of DANs (along the x axis).

(B and B') Confocal imaging of double labeling between PD2a1 and PD2b1 axons (labeled with GFP, green) and DAN dendrites (labeled with RFP, magenta). PAM-β'1 dendrites (B) and PAM-β'2m dendrites (B').

(C) Histogram of light microscopy overlap between a mask of PD2a1 and PD2b1 axons and masks of the dendrites of most MBONs (along the x axis).

(legend continued on next page)

R37G11-LexA, expressing in PD2a1 and PD2b1 neurons (Figures 8A and 8C, black lines).

We examined three DANs using double labeling. Both paired anterior medial (PAM)-β′1 and PAM-β′2 m dendrites interdigitated and exhibited potential synaptic contacts with PD2a1 and PD2b1 axons (Figures 8B and B'). PAM-β′2p had dendrites proximal to PD2a1 and PD2b1 axons but did not interdigitate (data not shown). PD2a1 and PD2b1-to-DAN connectivity may allow coordination of compartment activity across the MB (Cohn et al., 2015). PAM-β′2 m together with PAM-β′2p can drive approach behavior when stimulated (Lewis et al., 2015). Double labeling of MBON axons and PD2a1 and PD2b1 axons revealed close co-projection for MBON-β'2mp, MBON-γ2α′1, and MBON-α′2 (Figures 8D–8D''), indicating common postsynaptic partners or, possibly, axo-axonic synapses. MBON-β'2mp receives input from the MB compartment innervated by PAM-β'2 m and plays a role in appetitive and aversive memory retrieval (Owald et al., 2015). MBON-γ2α′1 drives approach when stimulated (Aso et al., 2014b). Silencing MBON-α′2 throughout training and testing abolishes appetitive memories (Aso et al., 2014b).

Spatial convergence of PD2a1 and PD2b1 and MBON axons could imply the existence of common downstream targets and/or axo-axonic synaptic interactions. To test this and validate our light-level double labeling, we returned to EM. We reconstructed MBON-α′2, the MBON that gave the highest PD2a1 and PD2b1 axon overlap score for MBONs (Figure 8C). We discovered that MBON-α′2 makes axo-axonic connections onto PD2a1 and PD2b1 neurons (Figures 8E–E'), indicating that PD2a1 and PD2b1 output may be modulated by MBON-α′2. The close proximity between axonal arbors required to make multiple axo-axonic synapses means that PD2a1 and PD2b1 and MBON-α′2 are well placed to share downstream targets. These data show that PD2a1 and PD2b1 axons interact with or converge with MB-associated neurons that drive approach behavior and memory retrieval.

DISCUSSION

In this study, we set out to identify how innate and learned representations interact using the tractable *Drosophila* brain. Previous work had identified an olfactory learned-to-innate axonal projection of neurons necessary for memory retrieval (Séjourné et al., 2011). Although MBON-α2sc also projects to several downstream brain regions, we hypothesized the existence of LH neurons that integrate innate and learned olfactory codes.

Using light and EM, we identified PD2a1 and PD2b1, an LH cell type that integrates both hardwired input and plastic memory information from the MB. By combining this analysis with double labeling, GRASP, thermogenetic mapping, and, eventually, neuronal reconstruction from EM, we confirm that PD2a1 and PD2b1 are directly postsynaptic to MBON-α2sc. Delineation of upstream PN connectivity also revealed that PD2a1 and PD2b1 dendrites in the dorsal LH mostly receive input from PNs encoding food or appetitive odors (Knaden et al., 2012; Semmelhack and Wang, 2009); this includes uniglomerular PNs from the DM1 and VA2 glomeruli, which are necessary for attraction to vinegar (Semmelhack and Wang, 2009). This connectivity matched the tuning of PD2a1 and PD2b1 cells, which was broad but included strong responses to apple cider vinegar, an appetitive odor. This suggests that PD2a1 and PD2b1 integrate innate and learned information and then pass this calculation to downstream circuits. We confirmed this by demonstrating that MBON-α2sc contributes significantly to the olfactory response of PD2a1 and PD2b1 for most odors.

Mirroring these anatomical and functional results, we found that PD2a1 and PD2b1 neurons are necessary for both aversive memory recall and innate olfactory attraction. Using specific split-GAL4 control of PD2a1 and PD2b1 neurons in the brain, we found that PD2a1 and PD2b1 signaling is necessary for memory retrieval across all phases but dispensable for innate olfactory aversion to the training odors (which are innately aversive). However, when animals were presented with food-related odors, which robustly generates olfactory attraction, silencing the PD2a1 and PD2b1 neurons abolished approach to a subset of odors. For the first time, to our knowledge, we have directly interrogated the role of LH neurons in olfactory behavior in adult *Drosophila*, discovering an LH cell type that is both necessary for innate attraction and, contrary to the assumption that the LH solely mediates innate behavior, also required for memory retrieval. Although information from the LH and MB must converge at some point in the fly brain to produce behavior, it is surprising that this integration happens within the LH rather than downstream of both the LH and MB. Indeed, MBON-α2sc mostly projects to other brain regions where MB and LH neurons converge (Aso et al., 2014b; Séjourné et al., 2011). This early convergence may minimize redundant circuitry (see below). We stress, however, that this does not preclude a role for other LH cell types in innate avoidance.

We developed a model for how this MB-to-LH circuit mediates aversive olfactory memory retrieval in the T maze assay (Figures 7D and 7E). As previous work has demonstrated, aversive olfactory conditioning induces synaptic depression at the MB-to-MBON synapse, which is thought to mediate memory retrieval (Bouzaiane et al., 2015; Hige et al., 2015a; Séjourné et al., 2011). However, the downstream circuits mediating the memory retrieval were unknown. We confirmed that PD2a1 and PD2b1 also depresses its response to the CS+, indicating that LH neurons can be modulated by MB activity. PD2a1 and PD2b1 are necessary for attraction, so the reduced drive in response to the CS+ results in less drive onto the approach circuits downstream (we have shown that PD2a1 and PD2b1 neurons are

(D–D'') Confocal imaging of double labeling between PD2a1 and PD2b1 axons (labeled with GFP, green) and MBONs (labeled with RFP, magenta). Shown are MBON-β'2mp axons (D), MBON-γ2α'1 axons (D'), and MBON -α′2 axons (D'').

(E) Visualization of MBON-α′2 axons interdigitating with PD2a1 and PD2b1 axons (black) in the EM volume. Other PD2a1 and PD2b1 neurons are shown in gray. Inset: positions of axo-axonic connections from MBON-α′2 onto PD2a1 and PD2b1 neurons, shown as cyan spheres.

(E') Summary of ipsilateral MBON-α′2′s axo-axonic connectivity onto PD2a1 and PD2b1 cells. Double labeling images are examples from n = 3 brains. For double labeling, the scale bar represents 5 μm.

cholinergic) (Figure 7E). In accordance with the prevailing view of how memory retrieval modulates the MB-to-MBON circuit, this model suggests that aversive olfactory memory retrieval is a result of modulating hardwired attraction circuits in response to the CS+ rather than the recruitment of a dedicated aversion module. However, we note that, in the T maze, the memory test is between two odors of similar innate valence. It is possible that other memory paradigms may recruit distinct aversion circuits; this may be a reason why a second MBON pathway for aversive memory recall exists in the *Drosophila* brain (Owald et al., 2015).

The identity of neurons downstream of PD2a1 and PD2b1 and their relationship to motor behavior is currently unknown. However, we demonstrate that PD2a1 and PD2b1 axons converge with MBONs implicated in memory and olfactory attraction. Downstream neurons may therefore read out both MB and PD2a1 and PD2b1 codes to guide the animal's choice. Future connectomics and functional approaches should identify these downstream neurons and their relationship to learned and unlearned sensory representations of different valence.

What are the implications of this circuit arrangement for learned and innate behavior? First, early integration of learned and innate pathways likely economizes neuronal hardware. Second, direct integration of learned and innate stimulus representations provides a simple mechanism to resolve the potentially conflicting behavioral drives that might exist after learning. Furthermore, this integration happens at a stage when neuronal activity is clearly sensory in character; this may be simpler than carrying out parallel sensory motor transformations downstream of both the MB and LH. One interesting hypothesis raised by the specific circuitry that we uncovered is that the balance between direct PN and indirect MBON-α2sc pathways onto PD2a1 and PD2b1 may constrain stimuli that can undergo aversive conditioning. Under our experimental conditions, apple cider vinegar odor responses were not altered by manipulating MBON-α2sc activity (whereas representations of some monomolecular appetitive odors could be modified). This may reflect selection on an evolutionary timescale of PN to LH connectivity to ensure that approach behavior produced by odors very highly predictive of food (and associated social interactions) is hard to reverse. Finally, it will be exciting to see whether a similar learned-to-innate circuit connectivity is involved in appetitive memory recall of other sensory modalities, such as taste and vision (Masek et al., 2015; Vogt et al., 2014).

The olfactory systems of flies and mammals share the same basic blueprint (Su et al., 2009; Wilson, 2008). In mice, the piriform cortex is required for learning and memory (Choi et al., 2011) and responds sparsely to odors (Stettler and Axel, 2009) and samples from the whole olfactory bulb (Miyamichi et al., 2011; Sosulski et al., 2011), similar to the MB. In contrast, the olfactory amygdala is necessary and sufficient to instruct innate olfactory behavior (Root et al., 2014) and receives stereotyped input from the olfactory bulb (Miyamichi et al., 2011; Sosulski et al., 2011), drawing a comparison to the LH. Intriguingly, there are uncharacterized connections between the piriform cortex and olfactory amygdala (Schwabe et al., 2004). A similar model of the piriform cortex modulating hardwired representations has been hypothesized in the mouse (Iurilli and Datta, 2017). We speculate that these connections may play a role in memory retrieval in the mammalian brain by enabling integration of learned and innate olfactory representations within the amygdala.

STAR★METHODS

Detailed methods are provided in the online version of this paper and include the following:

- KEY RESOURCES TABLE
- CONTACT FOR REAGENT AND RESOURCE SHARING
- EXPERIMENTAL MODEL AND SUBJECT DETAILS
- METHOD DETAILS
  - Molecular Biology
  - Immunohistochemistry
  - IHC Image Acquisition
  - Generation of split-GAL4 lines
  - Behavior: Olfactory Assays
  - Calcium Imaging: Functional Connectivity
  - Calcium Imaging: Olfactory Responses
  - Calcium Imaging: Olfactory Responses with MBON-α2sc silencing
  - Electrophysiology and olfactory stimulation
  - Sparse EM Reconstruction and Neuron Identification
- QUANTIFICATION AND STATISTICAL ANALYSIS
  - Image Processing and analysis
  - Neuronal Skeleton Data Analysis
  - Data Presentation
- DATA AND SOFTWARE AVAILABILITY
  - Data Availability
  - Code Availability

SUPPLEMENTAL INFORMATION

Supplemental Information includes eight figures, three tables, and one data file and can be found with this article online at https://doi.org/10.1016/j.neuron.2018.08.037.

A video abstract is available at https://doi.org/10.1016/j.neuron.2018.08.037#mmc7.

ACKNOWLEDGMENTS

This work was supported by MRC LMB graduate studentships and Boehringer Ingelheim Fonds PhD fellowships (to M.-J.D. and A.S.B.) and a Janelia graduate research fellowship (to M.-J.D.), ERC starting (211089) and consolidator (649111) grants and core support from the MRC (MC-U105188491) (to G.S.X.E.J.), Agence Nationale de la Recherche funding of the MemoNetworks and MemoMap projects (to P.-Y.P. and T.P.) and the Labex Memolife PhD fellowship (to G.B.-G.), the Howard Hughes Medical Institute (to A.W. and G.M.R.), a Wellcome Trust collaborative award (203261/Z/16/Z to G.S.X.E.J., D.B., and G.M.R.), and a Cambridge Neuroscience-PSL collaborative grant supported by the Embassy of France in London (to G.S.X.E.J.). This work was also supported by the HHMI Janelia Visiting Scientist Program. The Janelia FlyLight Project Team performed brain dissections, histological preparations, and confocal imaging for the PD2a1 and PD2b1 Split-GAL4 characterization, Polarity, and MCFO data. Heather Dionne cloned the R71D08-LexA construct and created split-GAL4 hemidriver lines. We thank the Bloomington Stock Center (NIH P40OD018537) and Matthias Landgraf for fly lines. We thank Paavo Huoviala for curating the behavioral significances of glomeruli. We thank Fiona Love, Adam Heath, Philipp Ranft, Amelia Edmondson-Stait, Kimberly Meechan, and Mahmoud Elbahnasawi for contributing 8% of

annotated synapses on PNs in the LH and Nadiya Sharifi for 4% of the MBON-α′2 cable. Finally we thank Marta Zlatic, Glenn Turner and his group, and the Jefferis and Preat groups for many insightful comments on the manuscript.

AUTHOR CONTRIBUTIONS

M.-J.D., P.-Y.P., T.P., and G.S.X.E.J. conceived the project and designed experiments. M.-J.D., G.B.-G., P.-Y.P., A.L.-S.-A., A.W., and A.H. performed experiments and analyzed data. S.F. performed electrophysiology and analysis. A.S.B., R.J.V.R., P.S., and G.S.X.E.J. carried out EM tracing and data analysis. S.F., Y.A., D.B., and G.M.R. contributed novel reagents and tools. The manuscript was written by M.-J.D., A.S.B., P.-Y.P., T.P., and G.S.X.E.J.

DECLARATION OF INTERESTS

The authors declare no competing interests.

Received: August 31, 2017
Revised: May 18, 2018
Accepted: August 27, 2018
Published: September 20, 2018

REFERENCES

Aso, Y., Hattori, D., Yu, Y., Johnston, R.M., Iyer, N.A., Ngo, T.-T.B., Dionne, H., Abbott, L.F., Axel, R., Tanimoto, H., and Rubin, G.M. (2014a). The neuronal architecture of the mushroom body provides a logic for associative learning. eLife *3*, e04577.

Aso, Y., Sitaraman, D., Ichinose, T., Kaun, K.R., Vogt, K., Belliart-Guérin, G., Plaçais, P.-Y., Robie, A.A., Yamagata, N., Schnaitmann, C., et al. (2014b). Mushroom body output neurons encode valence and guide memory-based action selection in Drosophila. eLife *3*, e04580.

Badel, L., Ohta, K., Tsuchimoto, Y., and Kazama, H. (2016). Decoding of context-dependent olfactory behavior in Drosophila. Neuron *91*, 155–167.

Bouzaiane, E., Trannoy, S., Scheunemann, L., Plaçais, P.-Y., and Preat, T. (2015). Two independent mushroom body output circuits retrieve the six discrete components of Drosophila aversive memory. Cell Rep. *11*, 1280–1292.

Caron, S.J.C., Ruta, V., Abbott, L.F., and Axel, R. (2013). Random convergence of olfactory inputs in the Drosophila mushroom body. Nature *497*, 113–117.

Chen, T.-W., Wardill, T.J., Sun, Y., Pulver, S.R., Renninger, S.L., Baohan, A., Schreiter, E.R., Kerr, R.A., Orger, M.B., Jayaraman, V., et al. (2013). Ultrasensitive fluorescent proteins for imaging neuronal activity. Nature *499*, 295–300.

Chen, X., Ma, W., Zhang, S., Paluch, J., Guo, W., and Dickman, D.K. (2017). The BLOC-1 subunit Pallidin facilitates activity-dependent synaptic vesicle recycling. eNeuro *4*, ENEURO.0335-16.2017.

Chiang, A.-S., Lin, C.-Y., Chuang, C.-C., Chang, H.-M., Hsieh, C.-H., Yeh, C.-W., Shih, C.-T., Wu, J.-J., Wang, G.-T., Chen, Y.-C., et al. (2011). Three-dimensional reconstruction of brain-wide wiring networks in Drosophila at single-cell resolution. Curr. Biol. *21*, 1–11.

Choi, G.B., Stettler, D.D., Kallman, B.R., Bhaskar, S.T., Fleischmann, A., and Axel, R. (2011). Driving opposing behaviors with ensembles of piriform neurons. Cell *146*, 1004–1015.

Christiansen, F., Zube, C., Andlauer, T.F.M., Wichmann, C., Fouquet, W., Owald, D., Mertel, S., Leiss, F., Tavosanis, G., Luna, A.J.F., et al. (2011). Presynapses in Kenyon cell dendrites in the mushroom body calyx of Drosophila. J. Neurosci. *31*, 9696–9707.

Cohn, R., Morantte, I., and Ruta, V. (2015). Coordinated and compartmentalized neuromodulation shapes sensory processing in Drosophila. Cell *163*, 1742–1755.

Costa, M., Manton, J.D., Ostrovsky, A.D., Prohaska, S., and Jefferis, G.S.X.E. (2016). NBLAST: rapid, sensitive comparison of neuronal structure and construction of neuron family databases. Neuron *91*, 293–311.

Diao, F., Ironfield, H., Luan, H., Diao, F., Shropshire, W.C., Ewer, J., Marr, E., Potter, C.J., Landgraf, M., and White, B.H. (2015). Plug-and-play genetic access to Drosophila cell types using exchangeable exon cassettes. Cell Rep. *10*, 1410–1421.

Fişek, M., and Wilson, R.I. (2014). Stereotyped connectivity and computations in higher-order olfactory neurons. Nat. Neurosci. *17*, 280–288.

Frechter, S., Bates, A.S., Tootoonian, S., Dolan, M.-J., Manton, J.D., Jamasb, A., Kohl, J., Bock, D., and Jefferis, G.S.X.E. (2018). Functional and anatomical specificity in a higher olfactory centre. bioRxiv. https://doi.org/10.1101/336982.

Gohl, D.M., Silies, M.A., Gao, X.J., Bhalerao, S., Luongo, F.J., Lin, C.-C., Potter, C.J., and Clandinin, T.R. (2011). A versatile in vivo system for directed dissection of gene expression patterns. Nat. Methods *8*, 231–237.

Gordon, M.D., and Scott, K. (2009). Motor control in a Drosophila taste circuit. Neuron *61*, 373–384.

Hamada, F.N., Rosenzweig, M., Kang, K., Pulver, S.R., Ghezzi, A., Jegla, T.J., and Garrity, P.A. (2008). An internal thermal sensor controlling temperature preference in Drosophila. Nature *454*, 217–220.

Heimbeck, G., Bugnon, V., Gendre, N., Keller, A., and Stocker, R.F. (2001). A central neural circuit for experience-independent olfactory and courtship behavior in Drosophila melanogaster. Proc. Natl. Acad. Sci. USA *98*, 15336–15341.

Hige, T., Aso, Y., Modi, M.N., Rubin, G.M., and Turner, G.C. (2015a). Heterosynaptic plasticity underlies aversive olfactory learning in Drosophila. Neuron *88*, 985–998.

Hige, T., Aso, Y., Rubin, G.M., and Turner, G.C. (2015b). Plasticity-driven individualization of olfactory coding in mushroom body output neurons. Nature *526*, 258–262.

Honegger, K.S., Campbell, R.A.A., and Turner, G.C. (2011). Cellular-resolution population imaging reveals robust sparse coding in the Drosophila mushroom body. J. Neurosci. *31*, 11772–11785.

Iurilli, G., and Datta, S.R. (2017). Population coding in an innately relevant olfactory area. Neuron *93*, 1180–1197.e7.

Jefferis, G.S.X.E., Potter, C.J., Chan, A.M., Marin, E.C., Rohlfing, T., Maurer, C.R., Jr., and Luo, L. (2007). Comprehensive maps of Drosophila higher olfactory centers: spatially segregated fruit and pheromone representation. Cell *128*, 1187–1203.

Jenett, A., Rubin, G.M., Ngo, T.-T.B., Shepherd, D., Murphy, C., Dionne, H., Pfeiffer, B.D., Cavallaro, A., Hall, D., Jeter, J., et al. (2012). A GAL4-driver line resource for Drosophila neurobiology. Cell Rep. *2*, 991–1001.

Keene, A.C., and Waddell, S. (2007). Drosophila olfactory memory: single genes to complex neural circuits. Nat. Rev. Neurosci. *8*, 341–354.

Keene, A.C., Stratmann, M., Keller, A., Perrat, P.N., Vosshall, L.B., and Waddell, S. (2004). Diverse odor-conditioned memories require uniquely timed dorsal paired medial neuron output. Neuron *44*, 521–533.

Kitamoto, T. (2001). Conditional modification of behavior in Drosophila by targeted expression of a temperature-sensitive shibire allele in defined neurons. J. Neurobiol. *47*, 81–92.

Knaden, M., Strutz, A., Ahsan, J., Sachse, S., and Hansson, B.S. (2012). Spatial representation of odorant valence in an insect brain. Cell Rep. *1*, 392–399.

Kohl, J., Ostrovsky, A.D., Frechter, S., and Jefferis, G.S.X.E. (2013). A bidirectional circuit switch reroutes pheromone signals in male and female brains. Cell *155*, 1610–1623.

Lewis, L.P.C., Siju, K.P., Aso, Y., Friedrich, A.B., Bulteel, A.J.B., Rubin, G.M., and Grunwald Kadow, I.C. (2015). A higher brain circuit for immediate integration of conflicting sensory information in Drosophila. Curr. Biol. *25*, 2203–2214.

Liang, L., Li, Y., Potter, C.J., Yizhar, O., Deisseroth, K., Tsien, R.W., and Luo, L. (2013). GABAergic projection neurons route selective olfactory inputs to specific higher-order neurons. Neuron *79*, 917–931.

Liu, C., Plaçais, P.-Y., Yamagata, N., Pfeiffer, B.D., Aso, Y., Friedrich, A.B., Siwanowicz, I., Rubin, G.M., Preat, T., and Tanimoto, H. (2012). A subset of

dopamine neurons signals reward for odour memory in Drosophila. Nature *488*, 512–516.

Luan, H., Peabody, N.C., Vinson, C.R., and White, B.H. (2006). Refined spatial manipulation of neuronal function by combinatorial restriction of transgene expression. Neuron *52*, 425–436.

Manton, J.D., Ostrovsky, A.D., Goetz, L., Costa, M., Rohlfing, T., and Jefferis, G.S.X.E. (2014). Combining genome-scale Drosophila 3D neuroanatomical data by bridging template brains. bioRxiv. https://doi.org/10.1101/006353.

Marin, E.C., Jefferis, G.S.X.E., Komiyama, T., Zhu, H., and Luo, L. (2002). Representation of the glomerular olfactory map in the Drosophila brain. Cell *109*, 243–255.

Masek, P., Worden, K., Aso, Y., Rubin, G.M., and Keene, A.C. (2015). A dopamine-modulated neural circuit regulating aversive taste memory in Drosophila. Curr. Biol. *25*, 1535–1541.

Masse, N.Y., Turner, G.C., and Jefferis, G.S.X.E. (2009). Olfactory information processing in Drosophila. Curr. Biol. *19*, R700–R713.

Miyamichi, K., Amat, F., Moussavi, F., Wang, C., Wickersham, I., Wall, N.R., Taniguchi, H., Tasic, B., Huang, Z.J., He, Z., et al. (2011). Cortical representations of olfactory input by trans-synaptic tracing. Nature *472*, 191–196.

Nern, A., Pfeiffer, B.D., and Rubin, G.M. (2015). Optimized tools for multicolor stochastic labeling reveal diverse stereotyped cell arrangements in the fly visual system. Proc. Natl. Acad. Sci. USA *112*, E2967–E2976.

Owald, D., Felsenberg, J., Talbot, C.B., Das, G., Perisse, E., Huetteroth, W., and Waddell, S. (2015). Activity of defined mushroom body output neurons underlies learned olfactory behavior in Drosophila. Neuron *86*, 417–427.

Parnas, M., Lin, A.C., Huetteroth, W., and Miesenböck, G. (2013). Odor discrimination in Drosophila: from neural population codes to behavior. Neuron *79*, 932–944.

Pfeiffer, B.D., Ngo, T.-T.B., Hibbard, K.L., Murphy, C., Jenett, A., Truman, J.W., and Rubin, G.M. (2010). Refinement of tools for targeted gene expression in Drosophila. Genetics *186*, 735–755.

Robinson, I.M., Ranjan, R., and Schwarz, T.L. (2002). Synaptotagmins I and IV promote transmitter release independently of Ca(2+) binding in the C(2)A domain. Nature *418*, 336–340.

Root, C.M., Denny, C.A., Hen, R., and Axel, R. (2014). The participation of cortical amygdala in innate, odour-driven behaviour. Nature *515*, 269–273.

Ruta, V., Datta, S.R., Vasconcelos, M.L., Freeland, J., Looger, L.L., and Axel, R. (2010). A dimorphic pheromone circuit in Drosophila from sensory input to descending output. Nature *468*, 686–690.

Saalfeld, S., Cardona, A., Hartenstein, V., and Tomancak, P. (2009). CATMAID: collaborative annotation toolkit for massive amounts of image data. Bioinformatics *25*, 1984–1986.

Schneider-Mizell, C.M., Gerhard, S., Longair, M., Kazimiers, T., Li, F., Zwart, M.F., Champion, A., Midgley, F.M., Fetter, R.D., Saalfeld, S., and Cardona, A. (2016). Quantitative neuroanatomy for connectomics in Drosophila. eLife *5*, e12059.

Schwabe, K., Ebert, U., and Löscher, W. (2004). The central piriform cortex: anatomical connections and anticonvulsant effect of GABA elevation in the kindling model. Neuroscience *126*, 727–741.

Séjourné, J., Plaçais, P.Y., Aso, Y., Siwanowicz, I., Trannoy, S., Thoma, V., Tedjakumala, S.R., Rubin, G.M., Tchénio, P., Ito, K., et al. (2011). Mushroom body efferent neurons responsible for aversive olfactory memory retrieval in Drosophila. Nat. Neurosci. *14*, 903–910.

Semmelhack, J.L., and Wang, J.W. (2009). Select Drosophila glomeruli mediate innate olfactory attraction and aversion. Nature *459*, 218–223.

Sosulski, D.L., Bloom, M.L., Cutforth, T., Axel, R., and Datta, S.R. (2011). Distinct representations of olfactory information in different cortical centres. Nature *472*, 213–216.

Stettler, D.D., and Axel, R. (2009). Representations of odor in the piriform cortex. Neuron *63*, 854–864.

Strutz, A., Soelter, J., Baschwitz, A., Farhan, A., Grabe, V., Rybak, J., Knaden, M., Schmuker, M., Hansson, B.S., and Sachse, S. (2014). Decoding odor quality and intensity in the Drosophila brain. eLife *3*, e04147.

Su, C.-Y., Menuz, K., and Carlson, J.R. (2009). Olfactory perception: receptors, cells, and circuits. Cell *139*, 45–59.

Tanaka, N.K., Endo, K., and Ito, K. (2012). Organization of antennal lobe-associated neurons in adult Drosophila melanogaster brain. J. Comp. Neurol. *520*, 4067–4130.

Tian, L., Hires, S.A., Mao, T., Huber, D., Chiappe, M.E., Chalasani, S.H., Petreanu, L., Akerboom, J., McKinney, S.A., Schreiter, E.R., et al. (2009). Imaging neural activity in worms, flies and mice with improved GCaMP calcium indicators. Nat. Methods *6*, 875–881.

Tully, T., Preat, T., Boynton, S.C., and Del Vecchio, M. (1994). Genetic dissection of consolidated memory in Drosophila. Cell *79*, 35–47.

Vogt, K., Schnaitmann, C., Dylla, K.V., Knapek, S., Aso, Y., Rubin, G.M., and Tanimoto, H. (2014). Shared mushroom body circuits underlie visual and olfactory memories in Drosophila. eLife *3*, e02395.

Wilson, R.I. (2008). Neural and behavioral mechanisms of olfactory perception. Curr. Opin. Neurobiol. *18*, 408–412.

Wong, A.M., Wang, J.W., and Axel, R. (2002). Spatial representation of the glomerular map in the Drosophila protocerebrum. Cell *109*, 229–241.

Zheng, Z., Lauritzen, J.S., Perlman, E., Robinson, C.G., Nichols, M., Milkie, D., Torrens, O., Price, J., Fisher, C.B., Sharifi, N., et al. (2018). A complete electron microscopy volume of the brain of adult Drosophila melanogaster. Cell *174*, 730–743.e22.

STAR★METHODS

KEY RESOURCES TABLE

REAGENT or RESOURCE	SOURCE	IDENTIFIER
Antibodies		
Chicken anti-GFP, 1/1600	Abcam	Catalog #: ab13970; RRID: AB_300798
Rabbit anti-RFP	Rockland	Catalog #: 600-401-379; RRID: AB_2209751
Mouse anti-Brp	DSHB, University of Iowa.	Catalog #: Nc82; RRID: AB_2314866
Mouse anti-GFP	Roche	Catalog #: 11814460001; RRID: AB_390913
Rabbit anti-GABA	Sigma-Aldrich	Catalog #: A2052; RRID: AB_477652
Mouse anti-ChaT	DSHB, University of Iowa.	Catalog #: 4B1; RRID: AB_528122
Rabbit anti-DvGlut	Gift from Dion Dickman, University of Southern California, USA (Chen et al., 2017)	N/A
Alexa 488 Goat anti-mouse IgG1	Thermo Fisher	Catalog #: A-21121; RRID: AB_141514
Alexa 488 Goat anti-chicken	Thermo Fisher	Catalog #: A-11039; RRID: AB_142924
Alexa-568 Goat anti-rabbit	Thermo Fisher	Catalog #: A-21069; RRID: AB_2535730
Alexa-568 Goat anti-mouse	Thermo Fisher	Catalog #: A-11004; RRID: AB_2534072
Alexa-633 Goat anti-mouse	Thermo Fisher	Catalog #: A-21126; RRID: AB_2535768
Rat anti-FLAG tag	Novus Biologicals	Catalog #: NBP1-06712SS; RRID: AB_1625982
Rabbit anti-HA tag	Cell Signaling Technologies	Catalog #: C29F4; RRID: AB_1549585
Mouse anti-V5	Biorad	Catalog #: MCA1360; RRID: AB_322378
Cy5 Donkey anti-Rat	Jackson Immuno Research	Catalog #: 712-175-150; RRID: AB_2340671
Cy3 Goat anti-Rabbit,	Jackson Immuno Research	Catalog #: 111-165-144; RRID: AB_2338006
Cy2 Goat anti-Mouse	Jackson Immuno Research	Catalog #: 610-611-002; RRID: AB_828261
Alexa-647 Donkey anti-Rat	Jackson Immuno Research	Catalog #: 712-605-153; RRID: AB_2340694
Streptavidin Alexa-647	Thermo Fisher	Catalog #: S-21374; RRID: AB_2336066
Experimental Models: Organisms/Strains		
w; +; 10XUAS-IVS-mCD8::GFP (attP2)	Bloomington Stock Center	Stock #: 32185; RRID: BDSC_32185
LexAop2-Brp(d3)::mCherry (su(hw)attP5)	M. Landgraf, University of Cambridge, UK	N/A
ChaMI^{04508}-LexA::QFAD	Diao et al. (2015) B. White, NIH, US	N/A
Insite0089-GAL4	Gohl et al. (2011) T. Clandinin, Stanford University, US	N/A
R58G03-GAL4	Bloomington Stock Center	Stock #: 39193; RRID: BDSC_39193
R37G11-GAL4	Bloomington Stock Center	Stock #: 49539; RRID: BDSC_49539
13xLexAop2-mCD8::GFP(su(Hw)attP8)	Bloomington Stock Center	Stock #: 32204; RRID: BDSC_39193
20XUAS-IVS-mCD8::GFP (attP2)	Bloomington Stock Center	Stock #: 32194; RRID: BDSC_32194
20xUAS-IVS-csChrimson::mVenus (attP18)	Bloomington Stock Center	Stock #: 55134; RRID: BDSC_55134
UAS-Shi^{ts1}	Bloomington Stock Center	Stock #: 44222; RRID: BDSC_44222
w; +; LexAop2-dTRPA1 (VK5)	Janelia Research Campus, USA	N/A
R37G11-LexA	Bloomington Stock Center	Stock #: 54238; RRID: BDSC_54238
R71D08-LexA (attp40)	Bloomington Stock Center	Stock #: 52841; RRID: BDSC_52841
w; +; UAS-GCaMP3 (VK5)	Bloomington Stock Center	Stock #: 32237; RRID: BDSC_32237
w, UAS-GCaMP6f (attP18)	Chen et al. (2013), Janelia Research Campus, USA	N/A
w; +; 3xUAS-Syt::smGFP-HA (su(Hw)attP1), 5xUAS-IVS-myr::smGFP-FLAG (VK5)	Aso et al. (2014a), Janelia Research Campus, USA	N/A

(*Continued on next page*)

Continued		
REAGENT or RESOURCE	SOURCE	IDENTIFIER
hsFlp2::PEST (attP3);+; 10XUAS-FRT > STOP > FRT-myr::smGFP-HA (VK00005), 10XUAS-FRT > STOP > FRT-myr::smGFP-V5-THS-10XUAS-FRT > STOP > FRT-myr::smGFP-FLAG (su(Hw)attP1)/ TM3, Sb	Bloomington Stock Center	Stock #: 64085; RRID: BDSC_64085
w, UAS-mCD8-GFP; UAS-mCD8-GFP	MRC Laboratory of Molecular Biology	N/A
w, LexAop2-CD8::GFP (su(Hw)attp8), 10xUAS-CD8::RFP (attp18)	Bloomington Stock Center	Stock #: 32229; RRID: BDSC_32229
MB025B	Bloomington Stock Center	Stock #: 68299; RRID: BDSC_68299
MB032B	Bloomington Stock Center	Stock #: 68302; RRID: BDSC_68302
MB018B	Bloomington Stock Center	Stock #: 68296; RRID: BDSC_68296
MB077B	Bloomington Stock Center	Stock #: 68283; RRID: BDSC_68283
MB002B	Bloomington Stock Center	Stock #: 68305; RRID: BDSC_68305
w;; LexAop2-Shi^{ts1} (VK00005)	Janelia Research Campus, USA	N/A
w; *20xUAS-GCaMP3 (attP40)*	Bloomington Stock Center	Stock #: 32116; RRID: BDSC_32116
Software and Algorithms		
NBLAST algorithm and R package	Costa et. al. (2016)	Website: http://jefferislab.org/si/nblast or https://github.com/jefferislab/nat.nblast
R neuroanatomy toolbox (nat) package	Jefferis et al. (2007); Costa et al. (2016)	Website: https://github.com/jefferis/nat
elmr	Zheng et al. (2018)	Website: https://github.com/jefferis/elmr
R flycircuit package	Costa et. al. (2016)	Website: https://github.com/jefferis/flycircuit
R catmaid package	Zheng et al. (2018)	Website: https://github.com/jefferis/rcatmaid
CATMAID	Saalfeld et al. (2009); Schneider-Mizell et al. (2016)	Website: https://github.com/catmaid/CATMAID
CMTK	N/A	Website: https://www.nitrc.org/projects/cmtk

CONTACT FOR REAGENT AND RESOURCE SHARING

Further information and requests for resources and reagents should be directed to and will be fulfilled by the Lead Contact, Gregory Jefferis (jefferis@mrc-lmb.cam.ac.uk).

EXPERIMENTAL MODEL AND SUBJECT DETAILS

Standard techniques were used for fly stock maintenance and construction. For imaging and immunohistochemistry flies were raised at 25°C on standard *Drosophila* food. For MultiColor FlpOut (MCFO) experiments (Nern et al., 2015), the MCFO stock (see below) was crossed to either R37G11-GAL4, LH989 or LH991. Flies were collected after eclosion, transferred to a new food vial and incubated in a 37°C water bath for 20-25 minutes.

Transgenic lines used for behavior were outcrossed for five generations to a *w1118* strain in a wild-type Canton-Special (CS) background. For behavioral experiments flies were raised at 18°C and 60% humidity under a 12-hr:12-hr light-dark cycle.

For a list of all genotypes used in each figure of the paper, see Table S1.

METHOD DETAILS

In all cases, sample size was based on previous studies (Bouzaiane et al., 2015; Gordon and Scott, 2009; Hige et al., 2015b). Experimenter blinding was not performed for experiments. No data was excluded from further analysis.

Molecular Biology

The pBP-R71D08 gateway entry construct was a kind gift from Heather Dionne. The insert was transferred to the pBPLexA::p65Uw destination vector (Addgene) via a Gateway LR recombination (Invitrogen).

The enhancers used to create split-GAL4 hemidrivers were created based on annotations for PD2a1 and PD2b1 in a GAL4 expression pattern database (Jenett et al., 2012). The enhancer hemidriver lines were created using Gateway cloning. All transgenic fly lines were generated by either Bestgene or Genetic Services.

Immunohistochemistry

Throughout this study we used two different immunohistochemistry (IHC) protocols. Figures 1F, 2A, 2B, and S5A used Protocol 2 while all other IHC data was processed using Protocol 1. For neurons filled during electrophysiology, see protocol for electrophysiological recording below. See Key Resources Table for antibodies used.

Protocol 1

IHCs were performed as described (Kohl et al., 2013). Fixation was in 4% paraformaldehyde for 20 minutes. Blocking was performed with normal goat serum overnight at 4°C. Primary and secondary antibody stains were incubated at 4% for 48 hours each. After incubation with both primary and secondary antibodies, the brains were washed with 0.5% Triton X-100 at room temperature. All specimens were mounted in Vectashield (H-1000) (Vector Laboratories, Burlingame, CA, USA).

Protocol 2

These IHCs were performed as described (Aso et al., 2014a). Dissected brains were fixed in 2% paraformaldehyde for 55 minutes at room temperature. Fix was removed and washed with 5% Triton X-100 at room temperature. Primary antibodies were incubated for 48 hours and secondary antibodies were incubated for 72 hours. A full step-by-step protocol can be found at https://www.janelia.org/project-team/flylight/protocols. Following the IHC protocol the brains were fixed again in 4% paraformaldehyde for four hours at room temperature. The brains were mounted on poly-L-lysine-coated coverslips and dehydrated through a series of ethanol baths (30%, 50%, 75%, 95%, and 3 × 100%) each for 10 min. Following dehydration they were submerged in 100% Xylene three times for 5 minutes each. Samples were embedded in DPX (DPX; Electron Microscopy Sciences, Hatfield, PA).

IHC Image Acquisition

All images for IHC were acquired using a Zeiss 710 Confocal Microscope (Aso et al., 2014a; Kohl et al., 2013). We used three modes of imaging: 20x, 40x and 63x.

For 20x imaging,

whole mount brain and VNCs were imaged using a Plan-Apochromat 20x/0.8 M27 objective (voxel size = 0.56 × 0.56 × 1.0 μm; 1024 × 1024 pixels per image plane).

20x imaging was used for Figures 2A and 2B.

For 40x imaging,

whole mount brains were imaged using an EC Plan-Neofluar 403/1.30 oil objective with 768 × 768 pixel resolution at each 1 μm, 0.6-0.7 zoom factor.

40x imaging was used for Figures 3A–3C, S1A, S1B, S1D, S2E, and S6.

For 63x imaging,

whole mount brains were imaged using a Plan-Apochromat 63x/1.40 oil immersion objective (voxel size = 0.19 × 0.19 × 0.38 μm; 1024 × 1024 pixels). For certain images, tiles of regions of interest were stitched together into the final image. 63x imaging was used for Figures 1C, 1F, 1G'', 8B, 8B', 8D–8D'', S1C, S2C, S2D, and S5A.

Generation of split-GAL4 lines

Each split-GAL4 line consists of two hemidrivers, the p65ADZp in attP40 and the ZpGAL4DBD in attP2 (Pfeiffer et al., 2010). The lines were screened by combining these two hemidrivers with a copy of *20xUAS-IVS-csChrimson::mVenus (attP18).* The brains of females from each line were dissected and screened with an epifluorescence microscope. Split-GAL4 combinations with favorable expression patterns (sparse expression of PD2a1 and PD2b1) were double balanced to make a stable stock.

Behavior: Olfactory Assays

See Table S3 for details of all olfactory stimuli used in behavior. For all behavior experiments, 0–2 day-old flies were transferred to fresh food vials the day before conditioning. Conditioning and tests of memory performance and of olfactory acuity were performed as described previously (Bouzaiane et al., 2015). Groups of 40-50 flies were trained with either one cycle of aversive training (single-cycle training), or five cycles spaced by 15 min inter-trial intervals (spaced training). During one cycle of training, flies were first exposed to an odorant (the CS+) for 1 min while 12 pulses of 1.5 s-long 60V electric shocks were delivered every 5 s; flies were then exposed 45 s later to a second odorant without shocks (the CS–) for 1 min. The odorants 3-octanol (Oct) and 4-methylcyclohexanol (Mch), diluted in paraffin oil at 0.360mM and 0.325mM respectively, were alternately used as CS. The test of memory performance was performed in a T-maze. Flies were placed at the convergence point of two airflows interlaced with Oct or Mch from either arm of the T-maze. After 1 min in the dark, flies were collected from both arms of the T-maze for subsequent counting, yielding a score calculated as $(N_{CS+} - N_{CS-}) / (N_{CS+} + N_{CS-})$. A single value of the performance index is the average of two scores obtained from two groups of genetically identical flies conditioned in two reciprocal experiments, using either odorant as CS+, and tested consecutively in the T-maze. Flies were maintained on food at all times, with the exception of during conditioning and memory test. Memory test occurred 10 ± 5 minutes after conditioning, 3h ± 30 minutes after conditioning, and 24 ± 1.5 h after conditioning to assay

immediate memory, 3-h memory and long-term memory, respectively. For long-term memory, flies were stored at 18°C after training which maximizes memory scores (Séjourné et al., 2011). For experiments involving neuronal blockade with Shi^{ts}, the time courses of the temperature shifts are provided alongside each graph of memory performance, and periods of neurotransmission blockade are highlighted in red. Flies were transferred to the restrictive temperature (33°C) 30 min before the targeted time, to allow for acclimatization to the new temperature.

To measure innate odor avoidance toward Oct or Mch, naive flies were placed at the convergence point of two airflows, one interlaced with Oct or Mch and the other from a bottle with paraffin oil only. The odor-interlaced side was alternated for successive groups that were tested. Odor concentrations used in this assay were the same as for memory assays. At these concentrations, both odorants are innately repulsive. The avoidance index was calculated the same way as the performance index in memory assays.

To measure innate odor approach, we used the avoidance assay with the same flow rate to deliver attractive odors. For apple cider vinegar experiments, the olfactory stimulus choice was between apple cider vinegar or water alone. Flies were starved on mineral water for 29h prior to experiments. The odor concentrations used were:

- Ethyl acetate: 10^{-7} in paraffin oil
- Isoamyl acetate: 10^{-6} in paraffin oil
- Apple cider vinegar: $6.1\text{x}10^{-5}$ in Evian mineral water

Starvation time and odor concentrations were determined beforehand using wild-type flies (data not shown) to obtain robust attractive behavior. The attraction index was calculated as the performance index multiplied by −1.

Performance, aversion and attraction indices are displayed as means ± SEM. A single value of the performance index is the average of two scores obtained from two groups of genetically identical flies conditioned in two reciprocal experiments, using either odorant as CS+, and tested consecutively in the T-maze. The indicated ‘n’ is the number of independent values of the performance index or avoidance index for each genotype. Memory graphs were subjected to statistical analysis using 1-way ANOVA followed by Newman-Keuls pairwise comparisons between the experimental group and its controls. ANOVA is robust against slight deviations from normal distributions or the inequality of variances if the sample sizes are similar between groups which was the case in our experiments. Therefore, we did not systematically test our data for normality or verify variance homogeneity prior to statistical tests, but we rather adopted a uniform analysis strategy for all our data ANOVA results are given as the value of the Fisher distribution F(x,y) obtained from the data, where x is the number of degrees of freedom between groups and y is the total number of degrees of freedom of the distribution. Statistical tests were performed using the GraphPad Prism 5 software. In the figures, asterisks illustrate the significance level of the t test, or of the least significant pairwise comparison following an ANOVA, with the following nomenclature: *$p < 0.05$; **$p < 0.01$; ***$p < 0.001$; NS: not significant, $p > 0.05$). See Table S2 for a detailed list of all odors used for behavioral and calcium imaging experiments.

Calcium Imaging: Functional Connectivity

The genetically encoded GCaMP6f calcium reporter (Chen et al., 2013) (*UAS-GCaMP6f* in *attp18*) was driven by *R37G11-GAL4 (attP2)*. The thermosensitive cation channel dTrpA1 (Hamada et al., 2008) (*LexAop2-dTrpA1 VK00005*) was expressed in the V2 neurons by the 71D08-LexA driver (attP40). Female flies of the indicated genotypes were prepared for *in vivo* imaging as described above, and mounted on a custom-made chamber with controlled temperature through a Peltier cell and an analog electronic PID circuit. The baseline setpoint for the temperature was 20°C. Imaging was performed on the same setup as for olfactory responses, images were acquired at a rate of one image every 640 ms. During an acquisition with thermal activation, the setpoint of the temperature control circuit was shifted to 31°C for 30 s after a baseline recording of 10 s, and then back to 20°C. The measured risetime of the temperature from 20°C to 29°C was ~8 s, and temperature reached 31°C within ~11 s. The temperature decrease was slower, taking ~15 s from 31°C to 22°C and ~25 s in total to decrease down to 20°C. For each fly, three such acquisitions were recorded, and the resulting time traces from visible hemispheres and from all these recordings were pooled and averaged. In R71D08LexA > LexAop2-TrpA1 flies, acquisitions with activation were alternated with acquisitions without activation as a permissive temperature control. The magnitude of activation was calculated as the mean of the time trace over a 20 s-time windows starting 5 s after the change in temperature setpoint.

Calcium Imaging: Olfactory Responses

To monitor the olfactory responses in PD2a1 and PD2b1 neurons, the genetically encoded GCaMP3 calcium reporter (Tian et al., 2009) was driven by R37G11 GAL4 driver. We used a transgenic line carrying the UAS-IVS-GCaMP3-p10 construct inserted on the third chromosome in VK00005 (Bouzaiane et al., 2015; Tian et al., 2009). For *in-vivo* imaging, one female fly was prepared for each n (Bouzaiane et al., 2015; Hige et al., 2015b; Séjourné et al., 2011). A cuticle window was removed in the back of the fly head. The fly was then placed under the objective lens (25x, 0.95 NA) of a confocal microscope under a constant airflow of 1.5 L·min-1. Images were acquired at a rate of one image every 128 ms. The emitted light was collected from transverse sections of the brain showing presynaptic terminals of PD2a1 and PD2b1 neurons. In general, both hemispheres could be recorded simultaneously. Olfactory stimuli were triggered by switching a valve to direct 30% of the total flow for 2 s through bottles containing odorants diluted in paraffin oil. Final dilution in the airflow was 1:2000. We recorded two series of responses to octanol and

methylcyclohexanol, in alternating order, each separated by a 2 min interval, but only the first response to each odorant was kept for analysis. Data analysis was performed with MATLAB software. For each recording, a $\Delta F/F0$ time trace trace was calculated from an ROI surrounding the PD2a1 and PD2b1 projections. The baseline F0 value was calculated from the 2 s period preceding the switch of the valve. The response integral was calculated as the integral of the time trace during 10 consecutive time points following the onset of odor response (approx. 2 s). The comparison of the response to a given odor between two groups, and of the response difference (Oct–Mch or Mch–Oct) between two groups, was performed using unpaired t test. The sample size was chosen according to the experiment, with olfactory response experiments having n = 6, similar to other naive imaging experiments (Hige et al., 2015a; Séjourné et al., 2011) For training and imaging experiments we chose a higher n, n = 19-22 or n = 9-11 for MTM and LTM respectively. As MTM is only partially abolished with PD2a1 and PD2b1 silencing we chose a higher n compared to LTM, which is entirely dependent on PD2a1 and PD2b1 (see Figures 1 and 2).

Calcium Imaging: Olfactory Responses with MBON-α2sc silencing

Flies were prepared for imaging as described above and imaging was performed within the same imaging cell as described above. Flies expressed GCaMP3 (attP40) through 37G11-GAL4 and LexAop2-Shi^{ts1} (VK00005) through 71D08-LexA (genotype controls had no LexA driver).

The concentrations used for imaging were:

- Ethyl acetate: 10μL in 100mL paraffin oil;
- Isoamyl acetate: 50μL in 100mL paraffin oil;
- Oct: 30 μL in 100mL oil;
- Mch: 100μL in 100mL oil;
- Apple Cider Vinegar: 5mL in 100mL mineral water.

See Table S3 for more information on these odors.

To avoid interactions between odorants, each fly received only one odor, 3 trials at low (23°C), 3 trials at high (33°) temperature. Each trial consisted of 2 s of odor stimulation. For each odor, half of the flies started at high temperature and the other half at low temperature. Trials were separated by 3 minutes, and after temperature shift, 8-10 minutes were left to get used to the new temperature. All trials at a given temperature were averaged, to give a single trace (DeltaF/F) and a single value of response integral per fly per temperature. For each fly both traces were normalized to the maximum value of the low temperature trace. The calcium traces displayed are the normalized time traces across all flies at each temperature. This normalization procedure better highlighted the effect of temperature shift independently of the absolute magnitude of the response. A paired t test was used to compare the response integral between the permissive and restrictive temperature.

Electrophysiology and olfactory stimulation

Recordings were carried out from PD2a1 and PD2b1 neurons in LH989 and LH991 split-GAL4 animals crossed to a UAS-CD8::GFP reporter. We recorded from n = 7 cells in total, 5 PD2a1 neurons, 1 PD2b1 neuron and one cell which which had an inconclusive dye-fill. Some odour concentrations that we eventually presented were not tested for all cells, hence n = 2-7 in total.

Female flies were sorted for correct genotype on day of eclosion using CO_2 anesthesia. One or two days later, the fly was cold-anesthetized and placed in a custom recording chamber for dissection as described previously (Kohl et al., 2013). The setup used for these experiments had a total of 64 channels. A full list of odors, solvents and dilutions used is provided in Table S3 below. The length of the valve opening stimulus was 2 s. The recording electrodes were 5 to 8 MΩ.

Odor stimuli were diluted in either mineral oil or water and were delivered via a custom odor delivery system (Kohl et al., 2013) (see jefferislab.org/resources). Unless otherwise indicated, liquid odors were diluted 1:500 (2 μl in 1ml) in either mineral oil or water. Solid odors were dissolved at 2mg in 1ml of solvent. During stimulus presentation, a portion of the airstream was switched from a solvent control to a selected odorant. The odorized air stream was then mixed with a clean carrier air stream at a 1:8 ratio to give a notional final dilution of 2.5×10^{-4} for most odors.

For labeling filled and recorded neurons, we used Alexa Fluor 568 (A11031, 1/1000) for the detection of mouse anti-nc82 and streptavidin Alexa fluor 647 (Thermo Fisher S-21374 1/4000) for detection of filled neurons. See Table S3 for list of odors used for electrophysiology.

Sparse EM Reconstruction and Neuron Identification

Neurons were reconstructed by 'tracing' in a full female adult *Drosophila melanogaster* brain volume (x,y,z resolution 4 nm x 4nm x 40 nm) that had been acquired by serial section transmission EM (Zheng et al., 2018), wherein the authors provide detailed sample preparation, EM acquisition and volume reconstruction protocols. Tracing aimed to generate a neuronal skeleton that represents the branching of neurons and the locations of their synapses, rather than a volumetric reconstruction. Manual neuronal tracing through EM serial sections was performed in CATMAID (http://www.catmaid.org) (Saalfeld et al., 2009), a Web-based environment for working on large image datasets that has been optimized for tracing and online analysis of neuronal skeletons (Schneider-Mizell et al., 2016). Neuronal skeleton reconstruction was performed consistent with Schneider-Mizell et al. (2016). Presynapses and

postsynapses were annotated for all neurons traced in this study. Polyadic synapses were marked consistent with the criterion of other CATMAID-based *Drosophila* connectomic studies (e.g., Zheng et al., 2018). Briefly, synapses must have a clear presynaptic density, multiple vesicles in the vicinity of the density and a cleft between the pre- and postsynaptic membranes. Postsynapses were marked if they had a (though often unclear or faint) postsynaptic density or otherwise distinctive morphology in apposition to the synaptic cleft. Additionally, for PD2a1/1 neurons, the point at which microtubules ceased to be apparent in a branch was also annotated. Microtubules appear as thin dark filaments that flow contiguously from the cell body and terminate before the lowest order branches. Ambiguities and uncertainties in each neuron were flagged as it was traced, all neurons were subsequently and iteratively proofread and edited by an expert tracer until completion at least in the region of interest (see below). Gap junctions could not reliably be identified in this dataset.

MBONs-α2sc and MBON-α′2 were found by tracing downstream of extant reconstructed Kenyon cells (Zheng et al., 2018) within the appropriate MB compartment (Aso et al., 2014a). Identity was verified with visual comparison to confocal stacks collected in Aso et al. (2014a). Identification of PD2a1 and PD2b1 cell types began with tracing downstream of right-side MBON-α2sc. 23.95% of 1837 total outgoing connections from the right-side MBON-α2sc axon in the LH were traced into 70 substantial neuronal arbors (> 300 μm of cable; data not shown). Visual inspection identified candidates for two PD2a1 neurons, which were traced to identification. This provided the location of the PD2 primary neurite tract (see Figure S7; nomenclature from Frechter et al., 2018). In insect brains, the majority neuronal cell bodies are positioned outside of the neuropil proper, in the cortex, and invaginate the neuropil via a primary neurite before branching. The primary neurite tract that a neuronal cell type takes is consistent between members of the type and between brains (S.F. and G.S.X.E.J., unpublished data). No similar tract that might have also contained our neurons of interest could be found after thorough visual scanning through the EM data, nor was there any indication from NBLAST clustering of LH neurons in the FlyCirciuit database (Chiang et al., 2011) or MCFO data that neurons similar to PD2a1 and PD2b1 could take multiple primary neurite tracts (data not shown). 185 neuronal profiles fasciculated within the PD2 tract, all of which were traced until their morphology made them an apparent PD2a1 and PD2b1 candidate or evidently not. Neurites for all candidates (34) were traced to or near 'completion' (see below). PD2a1 neurons must have 1) dendrite largely confined the the dorsal LH, 2) primary neurite tract in the PD2 bundle, 3) an axon that circumvents around the MB vertical lobe. Additionally PD2b1 neurons must have a process in the calyx. Two neurons met criterion 2 and 3, but were borderline on 1 and failed to receive similar projection neuron input to the 7 convincing members of the group, and were dropped from analysis. Identity was further verified by NBLAST (Costa et al., 2016) of reconstructed skeletons against MCFO data from this study and the FlyCircuit database (Chiang et al., 2011). Scores for our 7 putative PD2a1 and PD2b1 neurons were higher than for other candidate neurons in the PD2 tract and other MBON-α2sc targets (data not shown).

All 7 PD2a1 and PD2b1 neurons and the ipsilateral MBONs-α2sc and MBON-α′2 were fully traced 'to completion' *ex nihilo*, with synapse annotation. The contralateral MBONs-α2sc was traced to identification, but completed within the LH. 'Completion' does not necessarily mean that absolutely all cable has been reconstructed and postsynapses and presynapses annotated, as a small minority of processes and connections may not have been resolved due to ambiguities in the image data. Many uniglomerular, excitatory projection neurons of the medial antennal lobe tract had been identified in the present EM volume, and traced outside the MB calyx only to identification, not completion (Zheng et al., 2018). These PNs have since been reconstructed to completion in the LH (P.S. and A.S.B., unpublished data). For this study, we proofread, edited and annotated synapses for PN arbor in the right-side LH for all 20 uniglomerular PN types in the vicinity of PD2a1 and PD2b1 dendrite and those determined to have significant overlap at a light level (data not shown). At first, one representative PN was chosen for each glomerulus that produced more than one uniglomerular, excitatory PN. If this PN was found to synapse onto PD2a1 and PD2b1 neurons, its sister cells were also completed within the LH, as the morphology of sister PNs in the LH are extremely similar (Jefferis et al., 2007).

To identify neurons innervating PD2a1#1, we traced upstream of all of its dendritic postsynapses (i.e., postsynapses within the LH). Each upstream skeleton was traced to identification, i.e., the inclusion of a soma tract and main arbours, so as to ascertain whether it was a type of PN (axonic arbor in the LH, dendritic arbour in known second-order sensory neuropils), a LHON (axonic arbor leaving the LH), and LHLN (no significant arbor outside the LH), centrifugal neuron (axonic arbor within the LH, dendrites elsewhere in the superior protocerebrum) or MBON.

QUANTIFICATION AND STATISTICAL ANALYSIS

For all double labeling and imaging experiments, each n represents either a single slice or a volume from a single brain. For behavioral experiments, each n represents a group of 40-50 flies analyzed together in an olfactory assay. For functional connectivity and calcium imaging experiments, each n represents the response of a single recorded fly. For electrophysiology data, each n represents a recorded neuron from an individual fly (one neuron was recorded per fly).

Image Processing and analysis

To accurately label the presynapses of the LH-projecting MBONs, the 71D08-LexA driver was crossed to LexAop2-Brp(d3)::mCherry resulting in axon-specific labeling. For the region of the MBON under investigation (MBON-a2sc axons in the dorsal LH) a mask was created. Eight 71D08 > Brp(d3)::mCherry brains were immunostained and registered onto a common template brain (JFRC2, http://www.virtualflybrain.org) using the nc82 counterstain. Image registration was carried out as described (Kohl et al., 2013) using the

CMTK registration suite (https://www.nitrc.org/projects/cmtk). The boundary of the overlaid neurites for each region of interest from each brain was segmented manually as a mask in Fiji (https://fiji.sc/), using the Segmentation Editor function. The overlap was calculated against a large database of GAL4 expression patterns (Gohl et al., 2011; Jenett et al., 2012) also registered against JFRC2 (Manton et al., 2014) using the cmtk.statistics function in the open source nat (NeuroAnatomy Toolbox) package (https://github.com/jefferis/nat) for R (https://cran.r-project.org/). To control for the background signal of the brain we created a mask of the peduncle and performed the same overlap calculation for each GAL4 line. This peduncle overlap score was used normalize the MBON axon masks to produce the final overlap score for each GAL4 line. This allowed us to select lines with high signal-to-noise within the MBON masks and excluded expression patterns with Kenyon Cell expression which would confound behavioral analysis. The stacks GAL4 line expression patterns in the top 0.97 quartile were further analyzed manually to identify LH neurons.

For counting the number of cells in each line, images of R37G11-GAL4, LH989 and LH991 crossed to 20xUAS-csChrimson::mVenus (attp18) were used and cells manually counted.

MCFO brains were imaged in 63x mode (see above) and the stitched final image registered to the JFRC2013 template brain. Single neurons were manually annotated and segmented in 3D using Fluorender. For comparison with the data reconstructed from EM we automatically skeletonized MCFO image data using the filament editor tool provided by the image analysis software Amira 6.2.0, followed by manual editing. Morphological analysis was performed using NBLAST (see below). For analysis, neurons were segregated into soma, primary neurite (the neurite that leads to the cell body), dendrite, primary dendrite (the neurite connecting the dendritic and axonal arbors) and axon by visual inspection using insight from our EM data. We isolated 5 cells from R37G11-GAL4, 13 cells from LH989 and 5 cells from LH991. All lines contained neurons which projected to the MB calyx.

To examine overlap between PD2a1 and PD2b1 axons and MB neurons or PD2a1 and PD2b1 dendrites and PN axons, high resolution images (63x) of PD2a1 and PD2b1 split-GAL4 lines driving both a membrane and presynapse markers were segmented using Fluorender (http://www.sci.utah.edu/software/fluorender.html). We compared this to published segmentations of the DANs and MBONs (Aso et al., 2014a). All data were registered to the JFRC2013 template brain (Aso et al., 2014a). For all cell-types in addition to the entire membrane stain, the axons and dendrites were segmented separately for at least n = 2 well-registered brains. For each category of segmentation (dendrite-only, axon-only) we created a mask from their different samples by overlaying all the examples of each line. This was followed by contrast enhancement, Gaussian blurring and auto-thresholding to create a mask. All image processing was performed using Fiji. Overlap comparisons for pairs of masks were compared in R using the cmtk.statistics function in the "nat" package.

Double labeling images performed with R37G11-LexA and different MBON and DAN Split-GAL4 lines were processed with a median filter using the despeckle command in Fiji. This was necessary to remove background due to the weak expression levels of the R37G11-LexA line.

Neuronal Skeleton Data Analysis

Neuronal skeleton data from CATMAID were analyzed in R. Open source R packages for NBLAST (Costa et al., 2016), and R tools for accessing the CATMAID API are available on github by following links from jefferislab.org/resources. The catmaid and elmr R packages provide a bridge between a CATMAID server and the R statistical environment and bridging registration tools respectively. They include several add-on packages from the NeuroAnatomy Toolbox (nat see http://jefferis.github.io/nat/) suite enabling statistical analysis and geometric transformation of neuronal morphology. Further analysis relied on unreleased custom R code developed by A.S.B and G.S.X.E.J.

The elmr package provides tools for transforming data from the present EM whole female *Drosophila melanogaster* brain volume into different light level template brains for inspection of co-registered data. Neuronal skeleton reconstructions were brought from the EM brain space into the virtual flybrain template (http://www.virtualflybrain.org; dubbed JFRC2, the brain is divided into neuropils via the methods employed in Zheng et al., 2018). FlyCircuit PD2a1 and PD2b1 neurons, identified through NBLAST clustering, were brought into the JFRC2 brain space using the Computational Morphometry Toolkit (https://www.nitrc.org/projects/cmtk/).

MBONs-α2sc, MBON-α′2 and PD2a1 and PD2b1 neurons were segregated into axon and dendrites using a tcentrifugal synapse flow centrality algorithm (Schneider-Mizell et al., 2016)counting polyadic presynapses once). We verified that neurons were suitably polarized by calculating their axon-dendrite segregation index (Schneider-Mizell et al., 2016), which is a quantification for the degree of segregation of postsynapses and presynapses (0, totally unsegregated, 1, completely polarized). The mean ± SD segregation index for PD2a1 and PD2b1 neurons was 0.27 ± 0.09 indicating that these neurons are polarized but receive heavy axo-axonic modulation as well as outputting significantly in the LH. MBON were highly polarized, for example right-side MBONs-α2sc had a segregation index of 0.72. Again we counted polyadic presynapses once, rather than using the number of outgoing connections these make, which would have been more expensive in terms of tracing time.

For morphological analysis of PD2a1 and PD2b1 neurons NBLAST (Costa et al., 2016) was performed on either the dendritic and/or the axonal arbors of neuronal skeletons. Primary neurite tracts and the primary dendrites connecting dendritic and axonal arbors were removed because their fasciculation, especially in the single EM brain space, made NBLAST less sensitive to dendritic and axonal differences. Clustering was performed using functions for hierarchical clustering in base R on euclidean distance matrices of NBLAST scores, employing Ward's clustering criterion.

Data Presentation

All images of neuronal skeletons are shown in the JFRC2 brain space used by Virtual Fly Brain. Graphs were generated using the open source R package ggplot2 and related packages or GraphPad Prism 5 software.

DATA AND SOFTWARE AVAILABILITY

Data Availability

SWC files for the skeletonized multi-color flip-out data, and EM reconstructions for PD2a1 and PD2b1 neurons, and right-side MBON-α2sc and MBON-α′2 are available as a supplement to this paper (Data S1). Other data supporting the findings in this study are available upon request. A spreadsheet of glomeruli and published behavioral significance/functions is available upon request.

Code Availability

All R packages described above are available by following links from jefferislab.org/resources. Packages include full documentation and sample code. Custom scripts used to to generate figures can be made available upon request.

Neuron, Volume *100*

Supplemental Information

Communication from Learned to Innate Olfactory Processing Centers Is Required for Memory Retrieval in *Drosophila*

Michael-John Dolan, Ghislain Belliart-Guérin, Alexander Shakeel Bates, Shahar Frechter, Aurélie Lampin-Saint-Amaux, Yoshinori Aso, Ruairí J.V. Roberts, Philipp Schlegel, Allan Wong, Adnan Hammad, Davi Bock, Gerald M. Rubin, Thomas Preat, Pierre-Yves Plaçais, and Gregory S.X.E. Jefferis

Figure S1: Further anatomical analysis of 71D08-LexA and 37G11-GAL4. Related to Figure 1.

(A) Partial Z-projection of R71D08-LexA driving GFP. Expression of MBON-ɑ2sc is observed in the dorsal LH and along the vertical lobe of the MB. Representative image of n=3 stacks. (B-C) Immunohistochemistry of PD2a1/b1 to determine major neurotransmitter. (B) Colocalization of GABA (magenta) and R37G11-GAL driving GFP (green) expression indicates that PD2a1/b1 neurons are not GABAergic. (C) Colocalization of VGlut (magenta) and split-GAL4 line LH989 driving mVenus (green) expression indicates that PD2a1/b1 neurons are not glutamatergic (yellow arrows label sample bouton). For B-C the left panel is a composite while the middle and right panels represent single channels. (D) Immunohistochemistry of LH989 driving RFP in PD2a1/b1 neurons (green) and GFP genetically driven in cholinergic neurons by $Cha^{MI04508}$-LexA::QFAD. The cluster of PD2a1/b1 cell bodies is labelled with an orange circle. The scale bar for A and D is 30µm and 5µm for B-C. For B-D, each image is a representative slice from n=4 brains.

Figure S2: Anatomical and behavioural analysis of two other cell-types identified in the overlap screen. Related to Figure 1.

(A) A segmented image of AV6a1. (B) A segmented image of the identified SIP cell-type. (C) Z-projection of double labelling between AV6a1 dendrites and MBON-ɑ2sc. MBON-ɑ2sc presynapses are labelled with Brp(d3)::mCherry (magenta) while AV6a1 is labelled with membrane-bound GFP (in green). (D) Z-projection of double labelling between AV6a1 dendrites and MBON-ɑ2sc. The MBON-ɑ2sc presynapses are labelled with Brp(d3)::mCherry (magenta) while the SIP-1 neuron is labelled with membrane-bound GFP (in green). Note that this LexA line contains both MBON-ɑ2sc (dorsal) and MBON-α′3ap (ventral) axons (E) Unsegmented Z-projection of identified AV6a1 GAL4 line, Insite0089-GAL4, driving CD8::GFP (Green) with an nc82 counterstain (magenta). Representative stack from n=3 brains. This brain is registered to the JFRC2 template brain. (F) Unsegmented Z-projection of identified SIP-1 GAL4 line, R58G03-GAL4, driving CD8::GFP (Green) with an nc82 counterstain (magenta). This brain is registered to the JFRC2 template brain. Data from Virtual Fly Brain (http://www.virtualflybrain.org/site/vfb_site/home.htm) (G) Flies expressing Shi^{ts} driven by Insite0089-GAL4, and their genotypic controls, were trained with a single-cycle protocol at permissive temperature and tested 3h later at restrictive temperature. There was no significant difference in memory performance between the three groups ($n = 17$, $F_{(2,50)} = 2.29$, $p = 0.11$). (H)

Flies expressing Shi^{ts} driven by R58G03-GAL4, and their genotypic controls, were trained with a single-cycle protocol at permissive temperature and tested 3h later at restrictive temperature. There was no significant difference in memory performance between the three groups (n = 14, $F_{(2,41)} = 1.61$, p = 0.21). Data are presented as mean±SEM. Scale bar is 30µm for A-B and E-F, 5µm for C-D.

Figure S3: Permissive temperature controls for 37G11-GAL4 memory retrieval experiments. Related to Figure 1.
When assayed at the permissive temperature, flies expressing Shi^{ts} through R37G11-GAL4 driver performed similarly to their controls for immediate memory (A: n = 8, $F_{(2,23)} = 3.52$, p = 0.048, no difference between groups in pairwise comparisons), 3h-memory (B: n = 16, $F_{(2,47)} = 4.23$, p = 0.02, no difference between +/UAS-Shi^{ts} and 37G11>UAS-Shi^{ts} groups in pairwise comparisons), and long-term memory (C: n = 9–10, $F_{(2,28)} = 0.53$, p = 0.59). Data are presented as mean±SEM.

Figure S4: Permissive temperature controls for split-GAL4 memory retrieval experiments. Related to Figure 2.
When assayed at the permissive temperature, flies expressing Shi^{ts} through LH989 and LH991 performed similarly to their controls for immediate memory after 1x training (A: LH991, n = 12, $F_{(2,35)} = 0.14$, p = 0.87; B: LH989, n = 12–13, $F_{(2,36)} = 3.66$, p = 0.037, no difference between 37G11/+ and 37G11>UAS-Shi^{ts} groups in pairwise comparisons), for 3h-memory after 1x training (C: LH991, n = 15, $F_{(2,44)} = 0.28$, p = 0.76; D: LH989, n = 10, $F_{(2,29)} = 1.79$, p = 0.19), and for long-term memory after spaced training (E: LH991, n = 17, $F_{(2,47)} = 0.27$, p = 0.76; F: LH989, n = 7–9, $F_{(2,24)} = 2.10$, p = 0.15). Data are presented as mean±SEM.

Figure S5: Single-cell analysis of PD2a1/b1 GAL4 and split-GAL4 lines. Effectors in landing site VK5 and Electron microscopy reconstruction strategy. Related to Figure 2-4.
(A) An example a MultiColor FlipOut (MCFO) experiment. A single neuron is labelled in green while the counterstain is nc82 (magenta). The counterstain is registered to the JFRC2013 template brain (B) Skeletonized representations of all 22 single-neurons derived from MCFO registered to JFRC2013 (grey). (C-D) More detailed view of all 22 skeletons from an anterior (C) and dorsal (D) view. For C-D, only the LH (red) and calyx (blue) neuropil regions are labelled. Scale bar is 30µm. (E) Confocal z-projection of R71D08-LexA driving expression of

TdTomato (green) in landing site VK5. Expression recapitulates that of the R71D08 enhancer including MBON-ɑ2sc and MBON-α′3ap. Counterstain is nc82 (magenta). Representative stack of n=3 brains. (F) Cross-section of the PD2a1/b1 tract in the electron microscopy dataset. Neurons confirmed to be PD2a1/b1 are labelled in light (PD2a1) and dark green (PD2b1). Possible primary neurites in the PD2 tract are labelled with a purple dot (178). The x,y,z position of the tract in the ssTEM whole brain volume is 482684, 206960, 151200 nm, section 4320. (G) Dorsal view of PD2a1 (top) and PD2b1 (bottom) skeletons from MCFO data (blue) and EM reconstructions (green), shown with the LH (green), CA (purple), SIP (yellow) and SMP (orange). Black line bisecting PD2 primary neurite tract indicate position of EM section in (F). Slight offset between light and EM is thought to be a product of registration error (H'-H'') NBLAST clustering PD2a1 and PD2b1 arbors from EM reconstructed, FlyCircuit and MCFO data using only their axonal compartments (H') or dendritic compartments (H'') Skeletons are colored by the group to which they belong in the dendrogram. In (H) a1 and b1 neurons cluster apart. EM neurons are more similar to each other likely because they are from the same brain whereas the MCFO and FlyCircuit data are from different brains, and have been co-registered. In (H) the main two subdivisions denote the two routes PD2a1/b1 axons take around the MB (purple) vertical lobe. Labels name include the split-Gal4 line that generated the MCFO, or the Gal4 line driven by a neurotransmitter synthesis/processing protein used to generate the FlyCircuit flip-out data, or indicate an EM neuron in Figure 4B''.

Figure S6: Summary Electron Microscopy data for MBON-ɑ2sc and PD2a1/b1. Related to Figure 4.

(A) Bar chart showing the neuropils targeted by the ipsilateral 'fly's right' MBON-ɑ2sc axon, showing the postsynapses, presynapses and cable that the MBON has in each neuropil. Inset, presynapses across the MBON axon shown coloured by neuropil location. (B) The neuropils targeted by whole 'fly's right' PD2a1/b1 neurons and the PD2a1/b1 postsynapses, presynapses and cable length in each. Inset, postsynapses and presynapses across the PD2a1/b1 neurons shown coloured by neuropil location. (C) Stripchart shows the cable length in µms for PD2a1/b1 dendrites, PD2b1 dendrites and PDa1/b1 axons. Stacked bar chart shows the proportion of cable and postsynapses that were found on microtubule-containing backbone and microtubule free 'twigs'. Inset, visualisation of backbone (red) and twigs (green) in PD2a1#1. (D) Number of presynapses and postsynapses plotted for the presumptive axon and dendrite of individual PD2a1 and PD2b1 neurons. The different categories and region of the cell are represented by distinct

colors (PD2a1 or PD2b1) and shapes (Calyx, LH or whole compartments). Grey crosses indicate the mean. LH=lateral horn, MB_CA_R = mushroom body calyx, SIP=superior intermediate protocerebrum, SLP=superior lateral protocerebrum, SMP=superior medial protocerebrum, CRE = crepine, SCL = superior clamp, _R = fly's right (ipsilateral), _L = fly's left (contralateral).

Figure S7: Schematics for live calcium imaging training paradigms. Related to Figure 5. Schematics of the 3 hour conditioning protocols used for *in vivo* imaging of odor responses after training with Oct (red) as CS+ (A) or Mch (blue) as CS+ (B). Left represents the actual training paradigm where electric shock (orange spikes) is presented alongside the CS+. Right panel represents the control unpaired protocol where electric shock is presented two minutes before olfactory stimulation. (C) Each of the training paradigm in A-B is considered a single unit which is used to space train flies to generate LTM for imaging 24 hours after training (left panel). The training paradigm described in A or B (depending on CS+) is repeated 5 times with an intertrial interval (ITI) of 15 minutes. For the unpaired control at 24 hours (right panel), the unpaired protocols from A or B (depending on which odor is CS+) is repeated 5 times with an intertrial interval (ITI) of 15 minutes.

Figure S8: Permissive experiments for innate attraction experiments and genotype controls for MBON-ɑ2sc silencing during calcium imaging. Related to Figure 6 and 7. (A-D) Control experiments where animals did not have the LexAop-Shibire^{ts1} transgene to control for effects of the temperature shifts on olfactory coding. (A) Response of PD2a1/b1 axons to Oct, recorded by calcium imaging without MBON-ɑ2sc silencing. On left, time traces of normalized GCaMP3 fluorescence (see methods) are shown at permissive (blue) and restrictive (red) temperature in response to Oct stimulation (light blue bar). On right, the integral of the absolute odor responses for each individual fly at the permissive (blue) and restrictive (red) temperatures are plotted, which no change in response to Oct after MBON-ɑ2sc silencing (n=6, paired t-test=0.1). (B) Response of PD2a1/b1 axons to Mch, recorded by calcium imaging without MBON-ɑ2sc silencing. On left, time traces of normalized GCaMP3 fluorescence (see methods) are shown at permissive (blue) and restrictive (red) temperature in response to Mch stimulation (light blue bar). On right, the integral of the absolute odor responses for each individual fly at the permissive (blue) and restrictive (red) temperatures are plotted, which no change in response to Mch after MBON-ɑ2sc silencing (n=6, paired t-test=0.25). (C) Response of PD2a1/b1 axons to ethyl acetate, recorded by calcium imaging without MBON-ɑ2sc silencing.

On left, time traces of normalized GCaMP3 fluorescence (see methods) are shown at permissive (blue) and restrictive (red) temperature in response to ethyl acetate stimulation (light blue bar). On right, the integral of the absolute odor responses for each individual fly at the permissive (blue) and restrictive (red) temperatures are plotted, which no change in response to ethyl acetate after MBON-ɑ2sc silencing (n=6, paired t-test=0.98). (D) Response of PD2a1/b1 axons to vinegar, recorded by calcium imaging without MBON-ɑ2sc silencing. On left, time traces of normalized GCaMP3 fluorescence (see methods) are shown at permissive (blue) and restrictive (red) temperature in response to vinegar stimulation (light blue bar). On right, the integral of the absolute odor responses for each individual fly at the permissive (blue) and restrictive (red) temperatures are plotted, which no change in response to vinegar after MBON-ɑ2sc silencing (n=6, paired t-test=0.53). (E) Flies expressing Shi^{ts} driven by either LH989 or LH991 showed no impairment to apple cider vinegar relative to their genotype controls at the permissive temperature (n=9-10, $F_{(4,47)}$=0.42, p=0.79). (F) Flies expressing Shi^{ts} driven by either LH989 or LH991 showed no impairment to ethyl acetate relative to their genotype controls at the permissive temperature (n=8-11, $F_{(4,45)}$=0.41, p=0.80). Legend shows the shading representing each genotype.

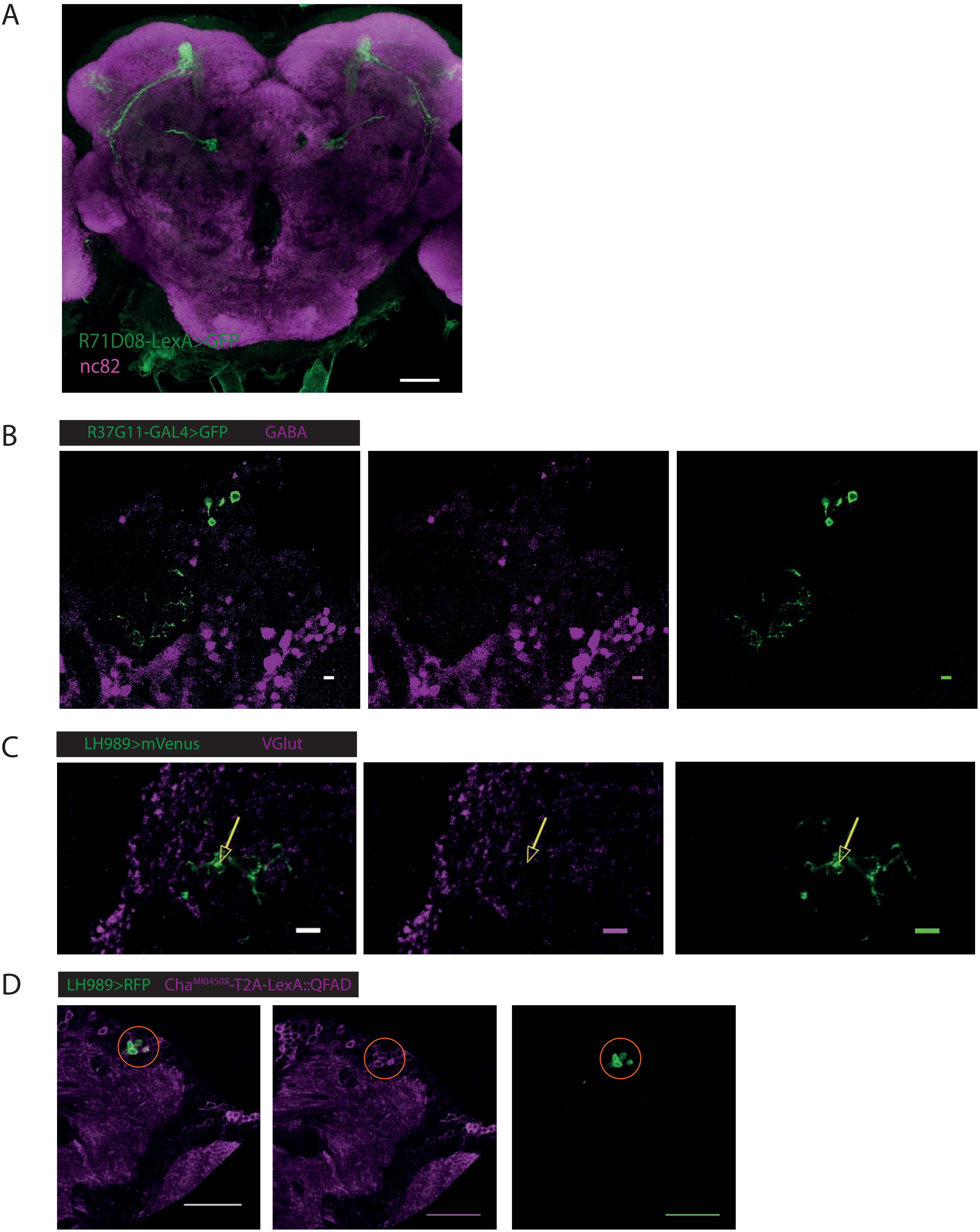
A
R71D08-LexA>GFP
nc82
B
R37G11-GAL4>GFP
GABA
C
LH989>mVenus
VGlut
D
LH989>RFP
Cha^{MI04508}-T2A-LexA::QFAD

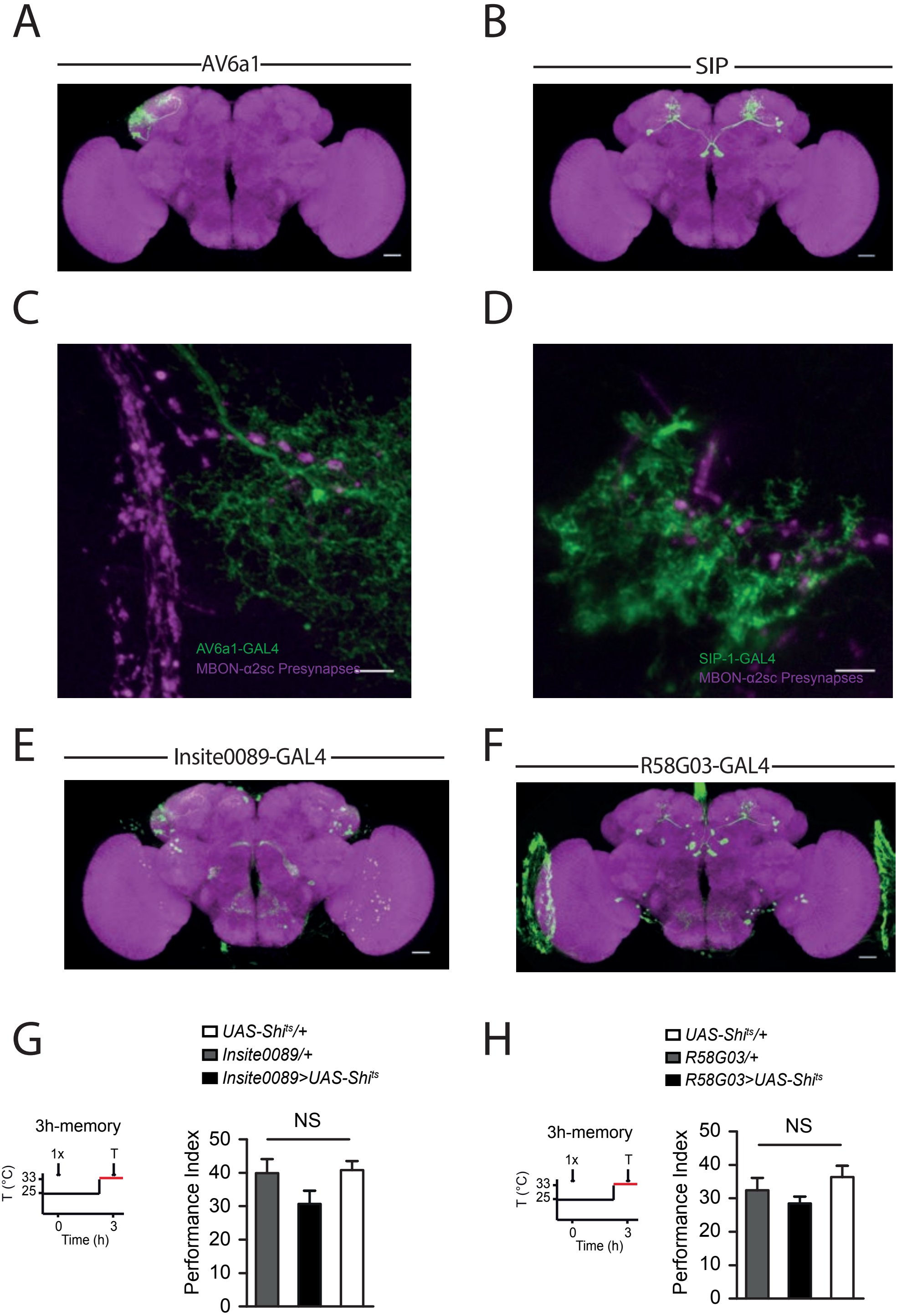

A
AV6a1
B
SIP
C
AV6a1-GAL4
MBON-α2sc Presynapses
D
SIP-1-GAL4
MBON-α2sc Presynapses
E
Insite0089-GAL4
F
R58G03-GAL4
G
UAS-Shi^ts/+
Insite0089/+
Insite0089>UAS-Shi^ts
3h-memory
1x
T
T (°C)
33
25
0
3
Time (h)
NS
Performance Index
50
40
30
20
10
0
H
UAS-Shi^ts/+
R58G03/+
R58G03>UAS-Shi^ts
3h-memory
1x
T
T (°C)
33
25
0
3
Time (h)
NS
Performance Index
50
40
30
20
10
0

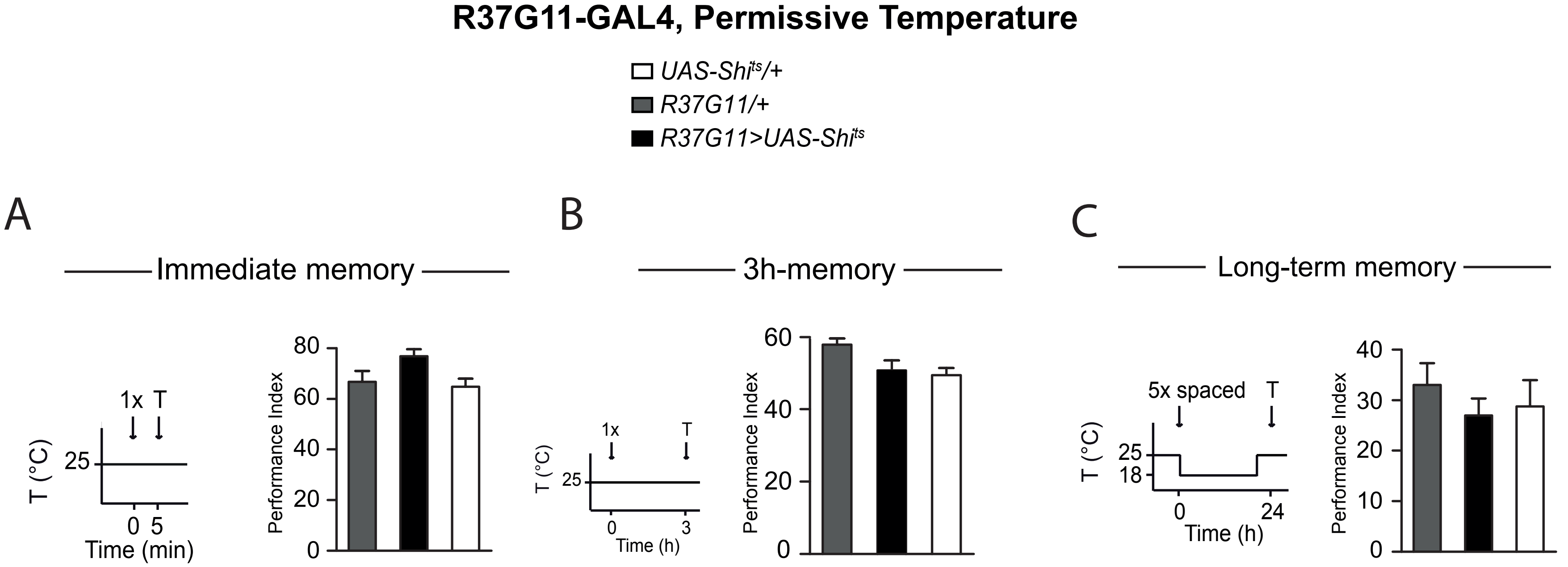

R37G11-GAL4, Permissive Temperature
UAS-Shi^ts/+
R37G11/+
R37G11>UAS-Shi^ts
A
Immediate memory
1x T
T (°C)
25
0 5
Time (min)
Performance Index
80
60
40
20
0
B
3h-memory
1x T
T (°C)
25
0 3
Time (h)
Performance Index
60
40
20
0
C
Long-term memory
5x spaced T
T (°C)
25
18
0 24
Time (h)
Performance Index
40
30
20
10
0

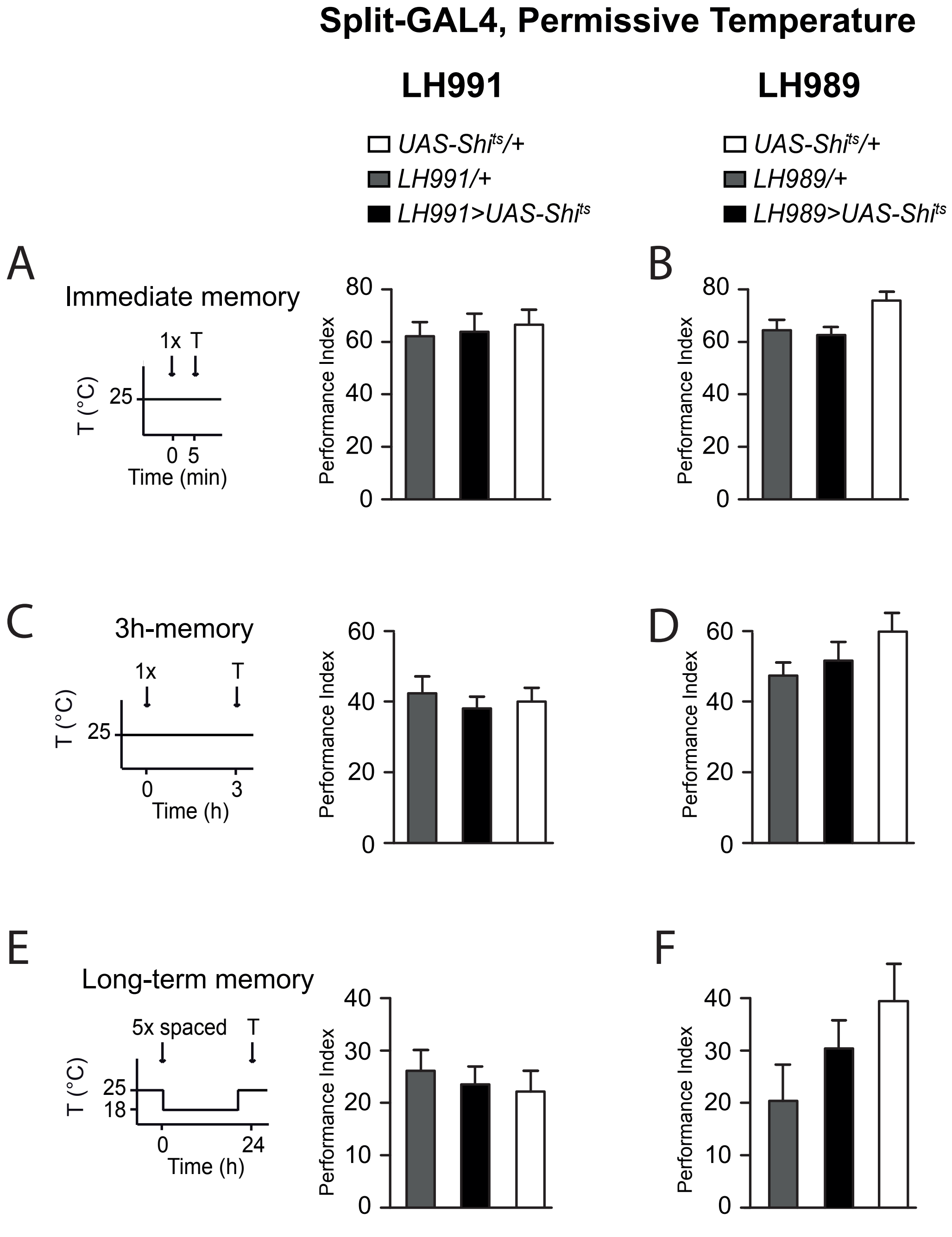
Split-GAL4, Permissive Temperature
LH991
LH989
UAS-Shits/+
LH991/+
LH991>UAS-Shits
UAS-Shits/+
LH989/+
LH989>UAS-Shits
A
Immediate memory
1x
T
25
T (°C)
0
5
Time (min)
Performance Index
80
60
40
20
0
B
C
3h-memory
1x
T
25
T (°C)
0
3
Time (h)
Performance Index
60
40
20
0
D
E
Long-term memory
5x spaced
T
25
18
T (°C)
0
24
Time (h)
Performance Index
40
30
20
10
0
F

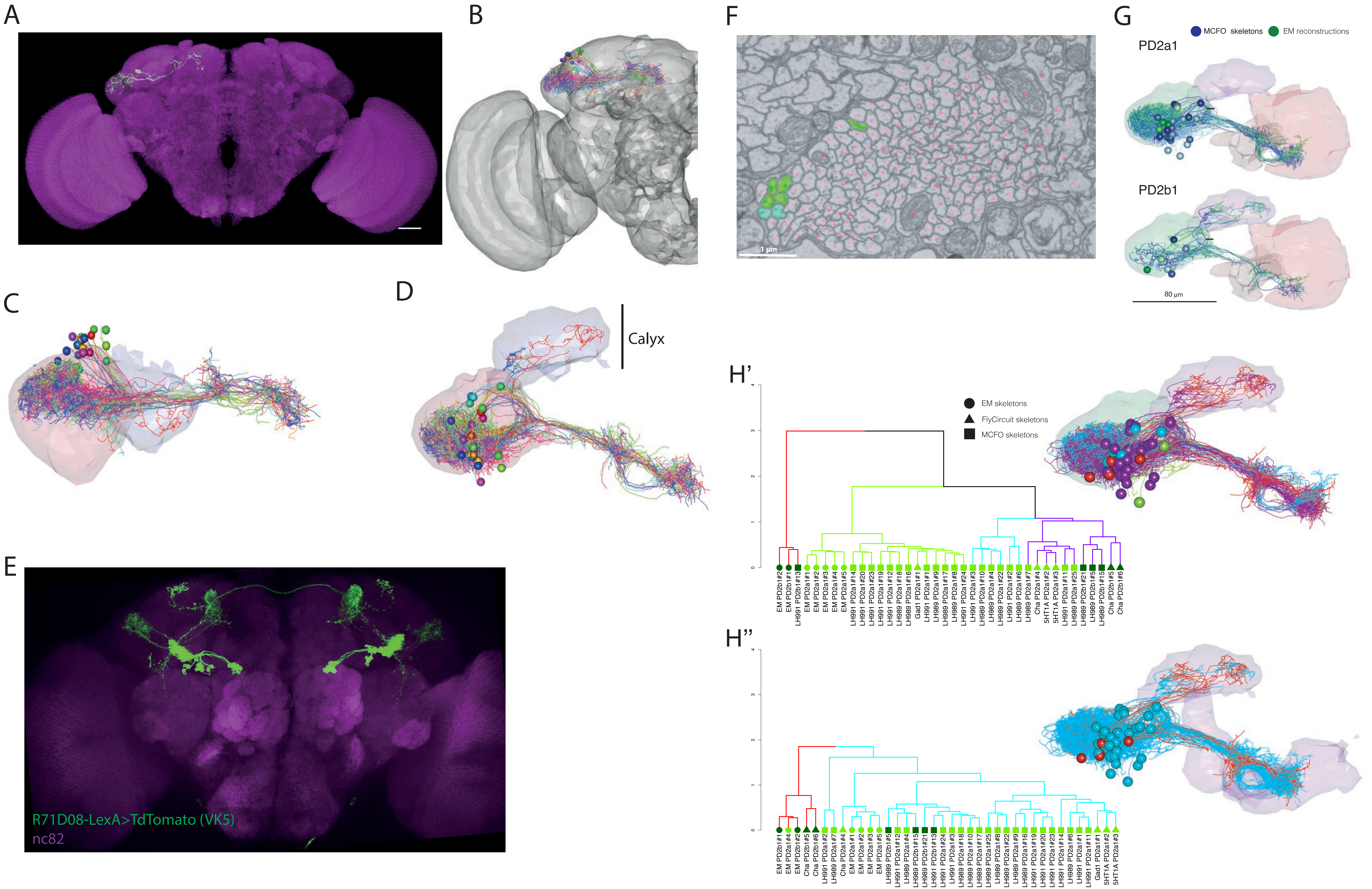
A
B
F
1 µm
G
MCFO skeletons
EM reconstructions
PD2a1
PD2b1
80 µm
C
D
Calyx
E
R71D08-LexA>TdTomato (VK5)
nc82
H'
EM skeletons
FlyCircuit skeletons
MCFO skeletons
0
1
2
3
4
EM PD2b1#2
EM PD2b1#1
LH991 PD2b1#13
EM PD2a1#1
EM PD2a1#2
EM PD2a1#3
EM PD2a1#4
EM PD2a1#5
LH991 PD2a1#14
LH991 PD2a1#20
LH991 PD2a1#23
LH991 PD2a1#19
LH991 PD2a1#12
LH989 PD2a1#18
LH989 PD2a1#16
Gad1 PD2a1#1
LH991 PD2a1#1
LH989 PD2a1#9
LH989 PD2a1#17
LH989 PD2a1#8
LH991 PD2a1#24
LH991 PD2a1#3
LH989 PD2a1#10
LH989 PD2a1#4
LH989 PD2a1#22
LH991 PD2a1#2
LH989 PD2a1#6
LH989 PD2a1#7
Cha PD2a1#4
5HT1A PD2a1#2
5HT1A PD2a1#3
LH991 PD2a1#11
LH989 PD2a1#25
LH989 PD2b1#21
LH989 PD2b1#5
LH989 PD2b1#15
Cha PD2b1#5
Cha PD2b1#6
H''
0
1
2
3
4
EM PD2b1#1
EM PD2a1#4
EM PD2b1#2
Cha PD2b1#5
Cha PD2b1#6
LH991 PD2a1#2
LH989 PD2a1#7
Cha PD2a1#4
EM PD2a1#1
EM PD2a1#2
EM PD2a1#3
EM PD2a1#5
LH989 PD2b1#5
LH991 PD2a1#12
LH989 PD2a1#4
LH989 PD2b1#15
LH989 PD2b1#21
LH991 PD2b1#13
LH991 PD2a1#24
LH991 PD2a1#3
LH989 PD2a1#18
LH989 PD2a1#10
LH989 PD2a1#17
LH989 PD2a1#25
LH989 PD2a1#8
LH989 PD2a1#22
LH989 PD2a1#9
LH989 PD2a1#16
LH991 PD2a1#19
LH991 PD2a1#20
LH991 PD2a1#23
LH991 PD2a1#14
LH989 PD2a1#6
LH991 PD2a1#1
LH991 PD2a1#11
Gad1 PD2a1#1
5HT1A PD2a1#2
5HT1A PD2a1#3

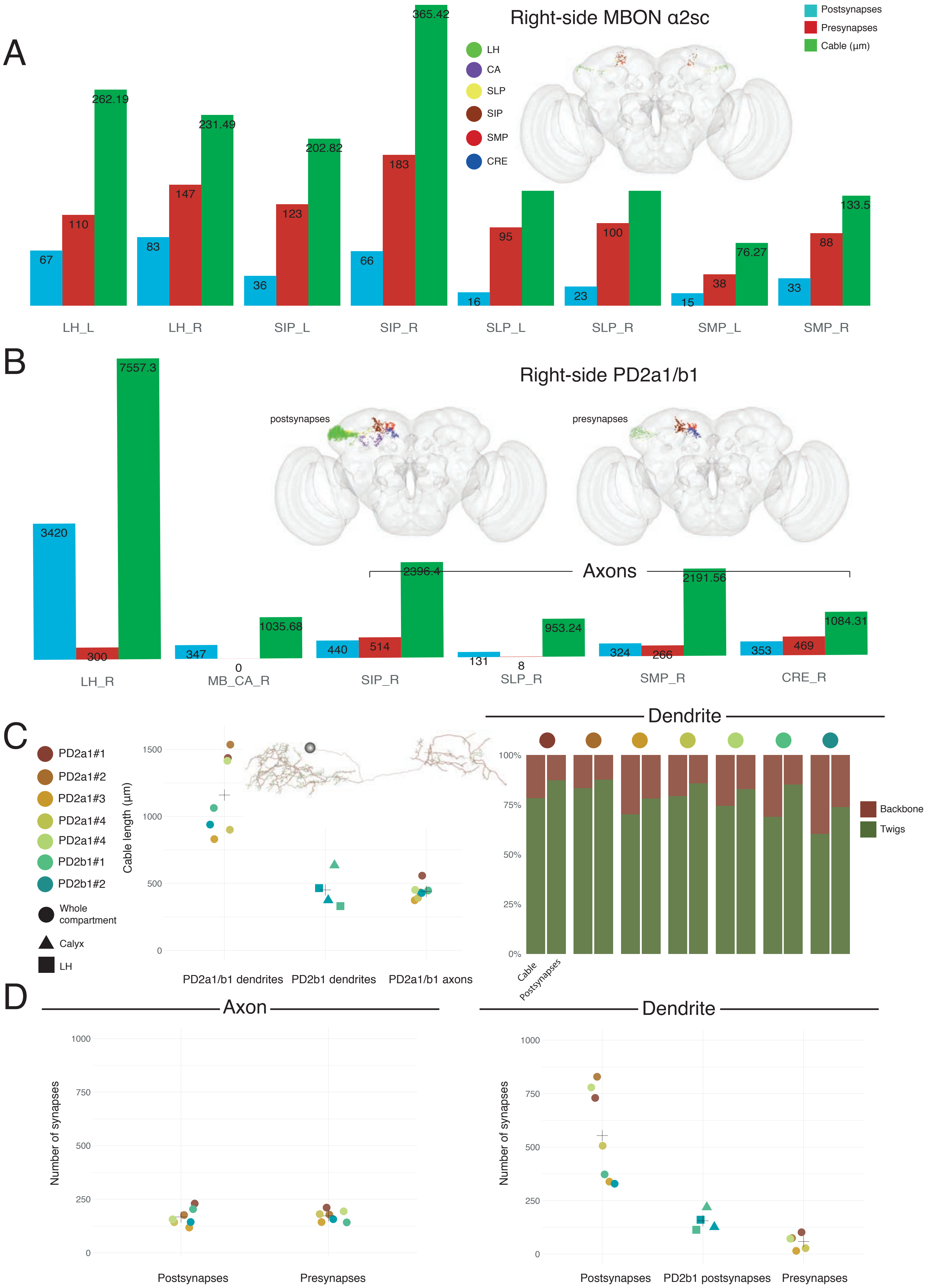
A
Right-side MBON α2sc
Postsynapses
Presynapses
Cable (μm)
LH
CA
SLP
SIP
SMP
CRE
67
110
262.19
83
147
231.49
36
123
202.82
66
183
365.42
16
95
100
23
15
38
76.27
33
88
133.5
LH_L
LH_R
SIP_L
SIP_R
SLP_L
SLP_R
SMP_L
SMP_R
B
Right-side PD2a1/b1
postsynapses
presynapses
3420
300
7557.3
347
0
1035.68
440
514
2396.4
Axons
131
8
953.24
324
266
2191.56
353
469
1084.31
LH_R
MB_CA_R
SIP_R
SLP_R
SMP_R
CRE_R
C
PD2a1#1
PD2a1#2
PD2a1#3
PD2a1#4
PD2a1#4
PD2b1#1
PD2b1#2
Whole compartment
Calyx
LH
Cable length (μm)
1500
1000
500
0
PD2a1/b1 dendrites
PD2b1 dendrites
PD2a1/b1 axons
Dendrite
100%
75%
50%
25%
0%
Backbone
Twigs
Cable
Postsynapses
D
Axon
Number of synapses
1000
750
500
250
0
Postsynapses
Presynapses
Dendrite
Number of synapses
1000
750
500
250
0
Postsynapses
PD2b1 postsynapses
Presynapses

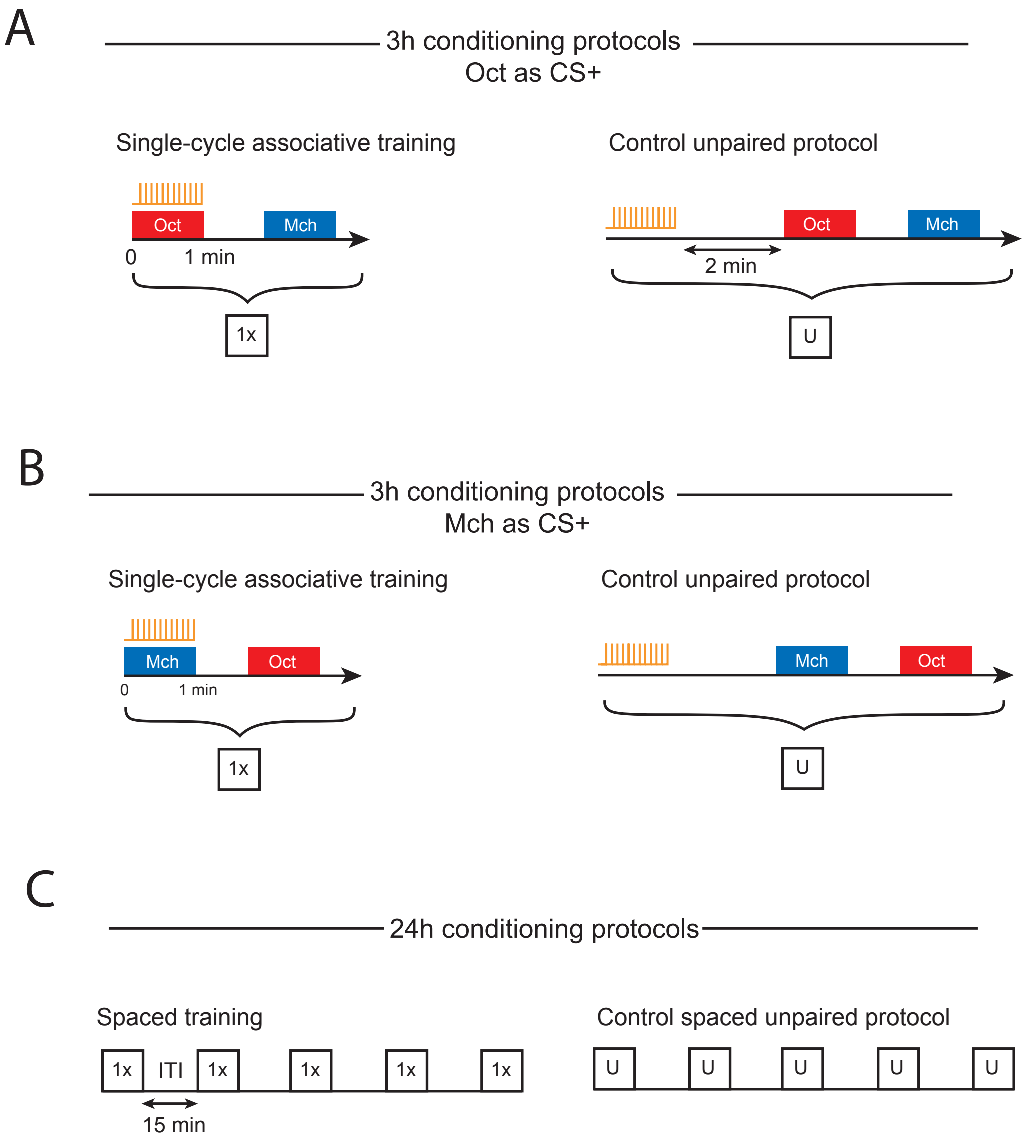
A
3h conditioning protocols
Oct as CS+
Single-cycle associative training
Oct
Mch
0
1 min
1x
Control unpaired protocol
Oct
Mch
2 min
U
B
3h conditioning protocols
Mch as CS+
Single-cycle associative training
Mch
Oct
0
1 min
1x
Control unpaired protocol
Mch
Oct
U
C
24h conditioning protocols
Spaced training
1x
ITI
1x
1x
1x
1x
15 min
Control spaced unpaired protocol
U
U
U
U
U

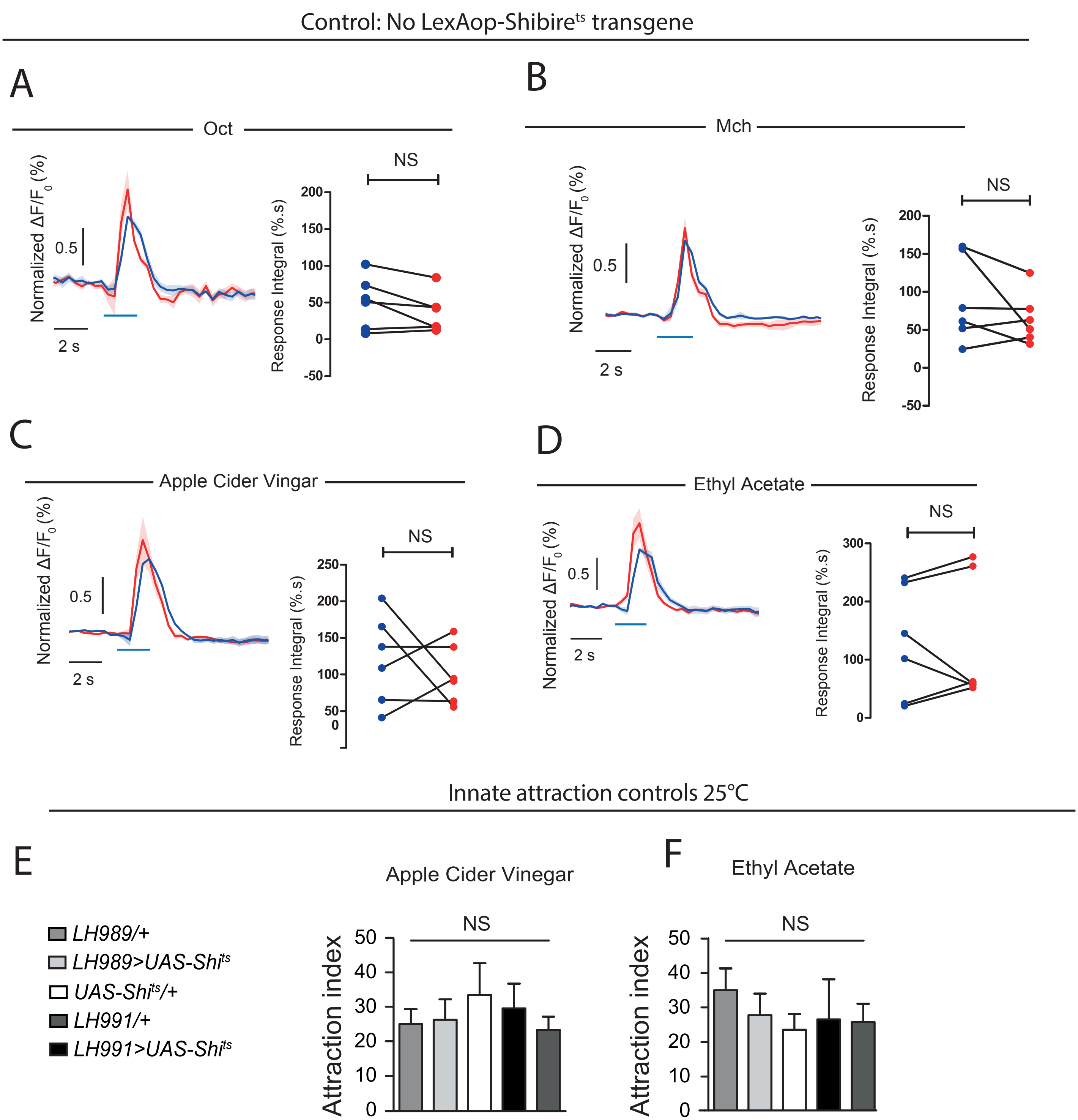

Control: No LexAop-Shibire ts transgene
A
Oct
Normalized ΔF/F0 (%)
0.5
2 s
NS
Response Integral (%.s)
200
150
100
50
0
-50
B
Mch
Normalized ΔF/F0 (%)
0.5
2 s
NS
Response Integral (%.s)
200
150
100
50
0
-50
C
Apple Cider Vingar
Normalized ΔF/F0 (%)
0.5
2 s
NS
Response Integral (%.s)
250
200
150
100
50
0
D
Ethyl Acetate
Normalized ΔF/F0 (%)
0.5
2 s
NS
Response Integral (%.s)
300
200
100
0
Innate attraction controls 25°C
E
LH989/+
LH989>UAS-Shits
UAS-Shits/+
LH991/+
LH991>UAS-Shits
Apple Cider Vinegar
NS
Attraction index
50
40
30
20
10
0
F
Ethyl Acetate
NS
Attraction index
50
40
30
20
10
0
